# Impact of rescanning and repositioning on radiomic features employing a multi-object phantom in magnetic resonance imaging

**DOI:** 10.1038/s41598-021-93756-x

**Published:** 2021-07-09

**Authors:** Simon Bernatz, Yauheniya Zhdanovich, Jörg Ackermann, Ina Koch, Peter J. Wild, Daniel Pinto dos Santos, Thomas J. Vogl, Benjamin Kaltenbach, Nicolas Rosbach

**Affiliations:** 1 Department of Diagnostic and Interventional Radiology, Institute for Diagnostic and Interventional Radiology, University Hospital Frankfurt, Goethe University Frankfurt am Main, Theodor-Stern-Kai 7, 60590 Frankfurt am Main, Germany; 2 Dr. Senckenberg Institute for Pathology, University Hospital Frankfurt, Goethe University Frankfurt am Main, 60590 Frankfurt am Main, Germany; 3 Frankfurt Cancer Institute (FCI), 60590 Frankfurt am Main, Germany; 4 Department of Molecular Bioinformatics, Institute of Computer Science, Johann Wolfgang Goethe-University, 60325 Frankfurt am Main, Germany; 5 Frankfurt Institute for Advanced Studies (FIAS), 60438 Frankfurt am Main, Germany; 6 Department of Radiology, University Hospital of Cologne, Kerpener Str. 62, 50937 Cologne, Germany

**Keywords:** Diagnostic markers, Predictive markers, Prognostic markers, Preclinical research, Translational research

## Abstract

Our purpose was to analyze the robustness and reproducibility of magnetic resonance imaging (MRI) radiomic features. We constructed a multi-object fruit phantom to perform MRI acquisition as scan-rescan using a 3 Tesla MRI scanner. We applied T2-weighted (T2w) half-Fourier acquisition single-shot turbo spin-echo (HASTE), T2w turbo spin-echo (TSE), T2w fluid-attenuated inversion recovery (FLAIR), T2 map and T1-weighted (T1w) TSE. Images were resampled to isotropic voxels. Fruits were segmented. The workflow was repeated by a second reader and the first reader after a pause of one month. We applied PyRadiomics to extract 107 radiomic features per fruit and sequence from seven feature classes. We calculated concordance correlation coefficients (CCC) and dynamic range (DR) to obtain measurements of feature robustness. Intraclass correlation coefficient (ICC) was calculated to assess intra- and inter-observer reproducibility. We calculated Gini scores to test the pairwise discriminative power specific for the features and MRI sequences. We depict Bland Altmann plots of features with top discriminative power (Mann–Whitney U test). Shape features were the most robust feature class. T2 map was the most robust imaging technique (robust features (rf), n = 84). HASTE sequence led to the least amount of rf (n = 20). Intra-observer ICC was excellent (≥ 0.75) for nearly all features (max–min; 99.1–97.2%). Deterioration of ICC values was seen in the inter-observer analyses (max–min; 88.7–81.1%). Complete robustness across all sequences was found for 8 features. Shape features and T2 map yielded the highest pairwise discriminative performance. Radiomics validity depends on the MRI sequence and feature class. T2 map seems to be the most promising imaging technique with the highest feature robustness, high intra-/inter-observer reproducibility and most promising discriminative power.

## Introduction

Diagnostic radiology is based on visual-semantic reporting^1^. Radiomics describes quantitative computational data analysis by transforming images into mineable data^1^. It is hypothesized that imaging data exists beyond visual perception which can be extracted to build imaging phenotypes, leading the way to non-invasive precision medicine^1–3^. A radiomics pipeline consists of specific steps^1,2,4^: (I) image acquisition and reconstruction, (II) preprocessing and segmentation of volumes of interest (VOI), (III) radiomic features extraction, (IV) statistical analysis with clinical and biological data, and (V) model development applying machine learning algorithms. Each step is prone to bias^1,5–7^. Currently there are increasing concerns about the robustness, validity and interpretability of radiomics research^5,6,8–10^. Multicenter studies deal with multiple imaging scanners and vendors with various protocols of acquisition and reconstruction^4,7,10^. There is no uniform recommendation for image pre-processing^8,11^. Image segmentation is prone to inter-observer variance^12^. Feature extraction and definition can be highly variable as research groups may use house-build software, making reproducibility and comparability of data nearly impossible^5,6,10^. Therefore, application of open-source implementations like PyRadiomics is highly recommended^5,6,8,13^. Following features extraction, numerous ways exist to reduce feature dimensionality and to build predictive models^13,14^. The image biomarker standardization initiative (IBSI) works towards standardizing the methodology^11^. Furthermore, radiomic features may not provide unique and independent information but are prone to redundancy^15^. An increasing number of studies addresses potential weaknesses of radiomics research^5,6,8,9,14^. Welch et al.^6^ have demonstrated that the signature features studied in a groundbreaking work of Aerts et al.^3^ might have been surrogates of tumor volume. Schwier et al. have emphasized the need of highly transparent reporting of methodology^8^. Schwier et al. have shown that the methods of image preprocessing and feature extraction highly influence the repeatability of radiomic features^8^. They have urged caution in the interpretation of radiomics studies^8^. There is ongoing debate concerning the repeatability and robustness of radiomic features^5,9,16–18^. Baeßler et al. have constructed a multi-object phantom to acquire test–retest data using three sequences and two matrix sizes to investigate the repeatability and robustness of MRI radiomic features^9^. Matrix size has not impacted repeatability and fluid-attenuated inversion recovery (FLAIR) provided the highest amount of robust features^9^. In total, 45 features have been extracted with one third having been robust across all sequences^9^. Those features have been proposed to be reliably used in future clinical studies^9^. The aim of our study was to replicate parts of the study design of Baeßler et al.^9^ with novelty given by a different selection of sequences, inclusion of T2 mapping, extraction of more radiomic features and we performed discriminatory analyses of phantom-components. We aimed to tackle the analyzes of robustness and reproducibility of radiomic MRI features of Baeßler et al.^9^ in another institute and with a different MR scanner to obtain temporal and geographical external validation. We applied the supposed reference software package PyRadiomics^19^ to extract the quantitative imaging features.

## Results

### Robustness of features depends on the feature class

**Figure 1:**
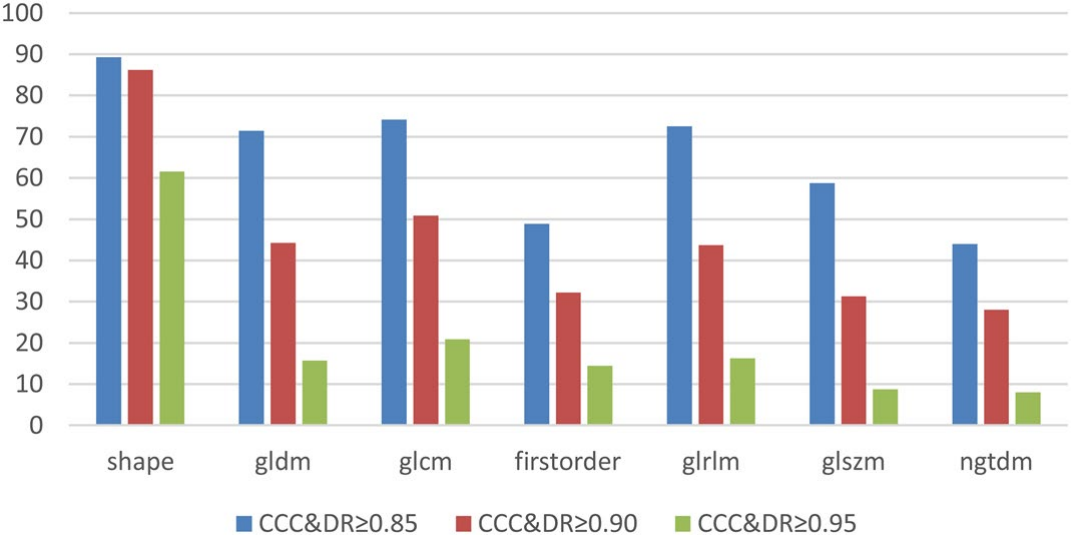
Feature class impacts the amount of robust features. Concordance correlation coefficient (CCC) and dynamic range (DR) values were computed for each feature. Results depict the combined mean values of dedicated CCC and DR analysis for each acquired MRI sequence plotted for each feature class. Excellent robustness was defined as CCC & DR ≥ 0.90 (red).

Figure 1 shows the fractions of robust features (CCC & DR ≥ 0.90, red) specific for the classes of features for the combined MRI sequences. Among the seven classes, shape has the highest fraction of 86.15% robust features. A fraction of 32.22% first-order features is robust. The least fraction of 28% robust features has class ngtdm. For all feature classes, the fraction of robust features rapidly decreases for increasing levels of robustness from relaxed (CCC & DR ≥ 0.85) to strict (CCC & DR ≥ 0.95).

### T2 map yields the highest fraction of robust features

**Figure 2:**
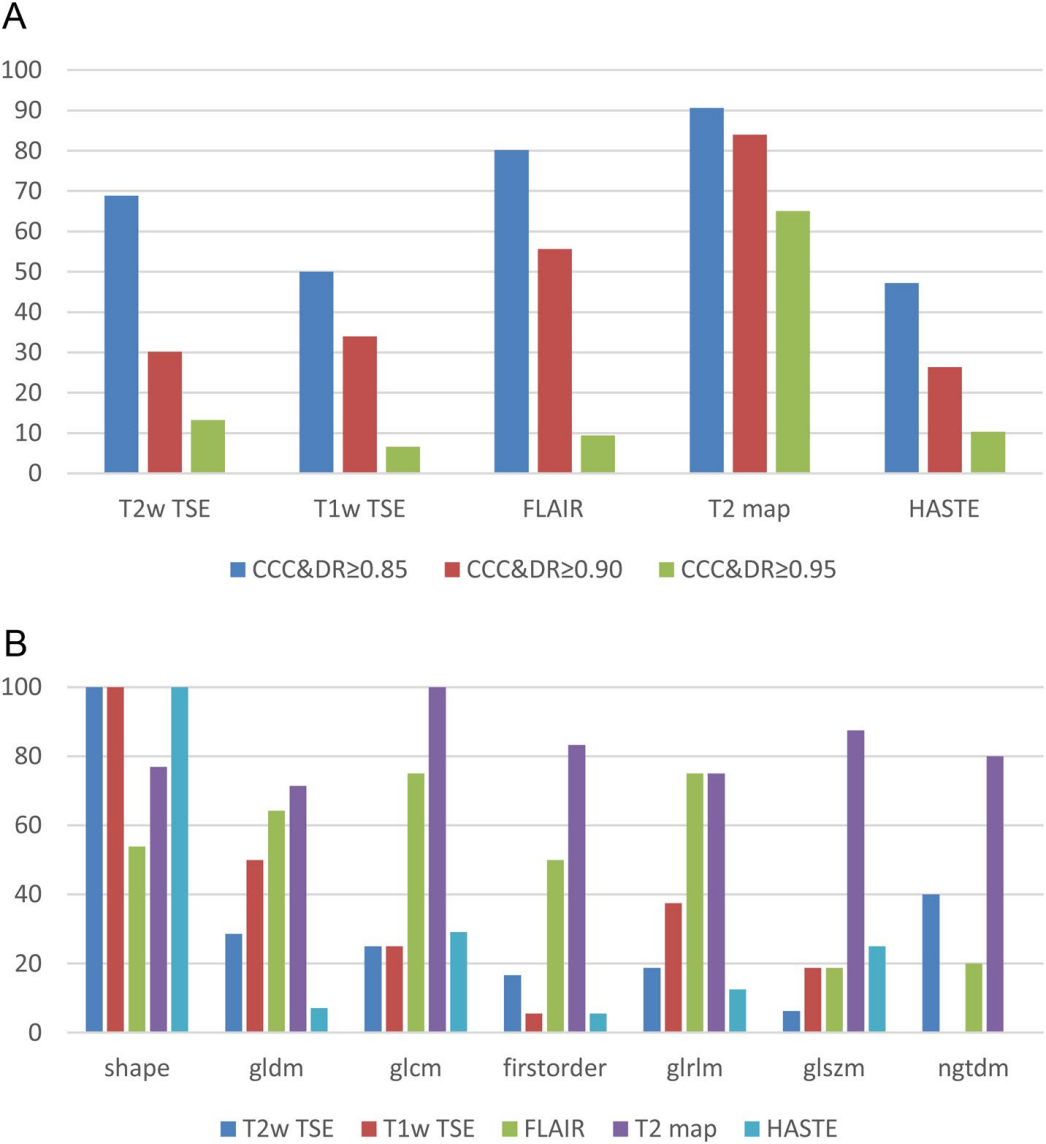
Impact of MRI sequences on the amount of robust features. Concordance correlation coefficient (CCC) and dynamic range (DR) values were computed for each feature and depicted for each MRI sequence (**A**) and feature class (**B**). The fraction of features decreases reciprocally to higher levels of robustness (CCC & DR ≥ 0.85; ≥ 0.90; ≥ 0.95) with T2 map revealing highest stability (**A**). We depict the distribution of excellently robust (CCC & DR ≥ 0.90) features in B. T2 map yields the highest fraction of robust features (**A**, **B**).

We stratified the fraction of robust features per MRI sequence and feature class, see Fig. 2A, B. T2 map yielded the highest fraction of 82.03% robust features (CCC & DR ≥ 0.90, red, Fig. 2A). Features of T2 map (dark violet bars, Fig. 2B) were robust in 100%, 87.50%; and 83.33% of the cases for glcm glszm, and first order, respectively. For the other classes, the fraction of robust features of T2 map ranged from 72.43% (gldm) to 80% (ngtdm). Shape feature class was the only feature class where T2 map did not yield the top result among the sequences with 77.00%. FLAIR was the sequence with the second highest fraction of 50.98% robust features (CCC & DR ≥ 0.90, red, Fig. 2A). FLAIR (green bars, Fig. 2B) obtained its highest fraction of robust features (75%) for glcm and glrlm, its lowest fraction (20%) for ngtdm. Compared to the other sequences, FLAIR had the least fraction (53.85%) of robust shape features. T2w TSE, T1w TSE, and HASTE (dark blue, red, light blue, Fig. 2B) yielded 100% robust shape features. Figure 2A shows the rapid decline of the fractions of robust features per sequence for increasing levels of robustness from relaxed (CCC & DR ≥ 0.85) to strict (CCC & DR ≥ 0.95). The results emphasize that, T2 map has advantages for all classes beside shape.

### Observer performance has excellent reproducibility

**Figure 3:**
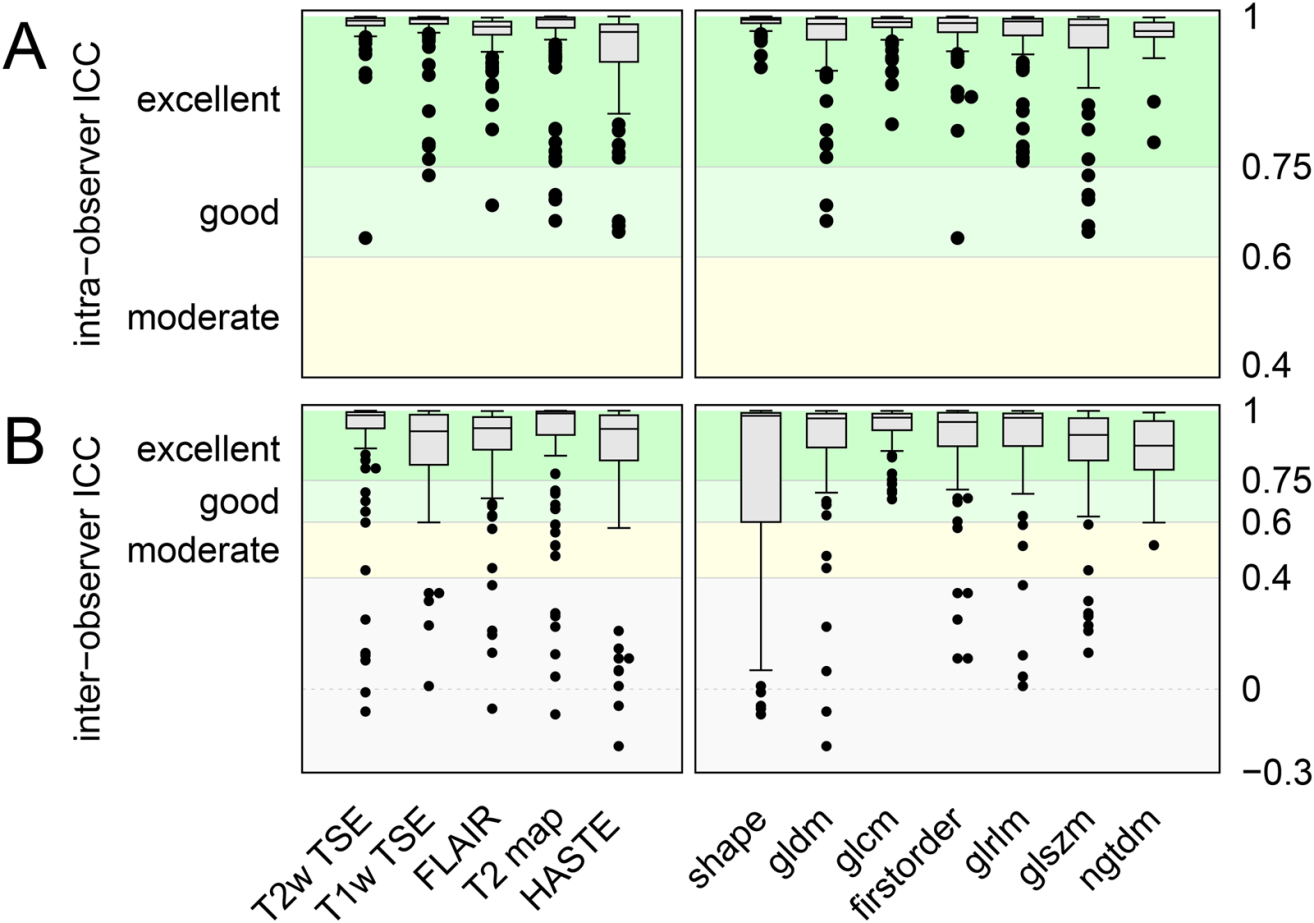
Inter-observer variance highly influences shape features. Box-Whisker plots for intraclass correlation coefficients are depicted (5–95 percentile) to visualize intra- (**A**) and inter-observer (**B**) reproducibility. To comprehensively visualize the effect of each feature, we performed single feature analysis with regard to the MRI sequence (left part) and feature class (right part) with outliers being depicted as dots (**A**, **B**).

The left part of Fig. 3A shows box-and-whisker plots of intra-observer ICCs of the features specific for the MRI sequences. The median values are excellent for all sequences, with a minimum median value of 0.94 and a maximum median value of 0.98 for HASTE and T2w TSE, respectively. Outlier values drop down to the minimum of 0.66 for T2w TSE. The right part of Fig. 3A shows box-and-whisker plots of intra-observer ICCs specific for the feature class. The median values of intra-observer ICC are excellent (≥ 0.95) for each feature class. Feature class shape shows preferable high median with small interquartile range. Left part of Fig. 3B shows corresponding box-and-whisker plots of inter-observer ICCs. As for intra-observer ICCs, the median values are excellent for all sequences, with a minimum median value of 0.83 and a maximum median value of 0.90 for HASTE and T2w TSE, respectively. Outlier values, however, drop down to values even below zero. Right part of Fig. 3B shows corresponding box-and-whisker plots of inter-observer ICCs specific for the feature class. The median values of inter-observer ICCs are excellent (≥ 0.95) for each feature class.

### T2 map inherits the highest robustness and reproducibility of features

We stratified feature subcohorts (CCC ≥ 0.90 & intra-/inter-ICC ≥ 0.75) to propose feature sets for each MRI imaging technique with excellent levels of robustness and reproducibility (Supplementary Table 1, Table A.1). T2 map yielded the highest number of robust and reproducible features (rrf, n = 84, Table 1). FLAIR was the second highest ranked MRI sequence (rrf, n = 59). The further MRI sequences revealed rrf of 20, 26, 29 for HASTE, T2w TSE and T1w TSE, respectively (Supplementary Table 1, Table A.1). A set of eight features was found to be robust and reproducible across all MRI sequences (Table 2). Seven out of these eight features were part of the shape feature class and the further remaining feature was Imc1 of glcm feature class (Table 2). Intra-observer ICC, inter-observer ICC, CCC, and DR for each feature are visualized in supplementary Fig. 1 (Fig. B.1). Supplementary Fig. 2 (Fig. C.1) shows the Bland Altmann plots for the set of the eight features that were robust and reproducible across all MRI sequences.

**Table 1:** T2 map—robust and reproducible features.

Features	CCC	DR	Intra-observer ICC	Inter-observer ICC
shape_Maximum3DDiameter	0.99205286	0.96251665	0.99716664	0.9867156
shape_MajorAxisLength	0.99793419	0.97981361	0.996171	0.99749429
shape_Elongation	0.9169208	0.92685464	0.99414914	0.99282215
shape_Maximum2DDiameterSlice	0.99383495	0.97822157	0.99761689	0.9936394
shape_SurfaceArea	0.99756368	0.93373906	0.99185998	0.87503941
shape_MinorAxisLength	0.99207422	0.96696735	0.99616827	0.99825783
shape_Maximum2DDiameterColumn	0.98837119	0.95757463	0.99194508	0.98736261
shape_Maximum2DDiameterRow	0.99209873	0.95395661	0.99386968	0.97679991
gldm_GrayLevelVariance	0.99604933	0.97096707	0.99580458	0.98995297
gldm_HighGrayLevelEmphasis	0.9987185	0.99001813	0.99867983	0.99895219
gldm_DependenceEntropy	0.9768604	0.93069293	0.97918853	0.95287381
gldm_GrayLevelNonUniformity	0.99672213	0.96844026	0.99404055	0.90588485
gldm_SmallDependenceEmphasis	0.99618473	0.97684637	0.99941963	0.99714743
gldm_SmallDependenceHighGrayLevelEmphasis	0.99785548	0.99026715	0.99849964	0.99848907
gldm_DependenceNonUniformityNormalized	0.98991352	0.97110551	0.99933187	0.99372596
gldm_LargeDependenceEmphasis	0.99604457	0.97768667	0.99663332	0.9956135
gldm_DependenceVariance	0.98074193	0.95832053	0.99468459	0.99131544
gldm_LargeDependenceHighGrayLevelEmphasis	0.91063923	0.95569232	0.99721749	0.9746251
glcm_JointAverage	0.99868486	0.98745931	0.99801085	0.99716702
glcm_SumAverage	0.99868486	0.98745931	0.99801085	0.99716702
glcm_JointEntropy	0.99586316	0.9719637	0.99865882	0.99477919
glcm_ClusterShade	0.9839736	0.96063188	0.99510842	0.99205699
glcm_MaximumProbability	0.97316718	0.95034713	0.98318707	0.97765663
glcm_Idmn	0.97708482	0.95808548	0.99843989	0.98992028
glcm_JointEnergy	0.99472158	0.97035282	0.98991929	0.9924416
glcm_Contrast	0.99878665	0.98414808	0.99899223	0.99747783
glcm_DifferenceEntropy	0.99888168	0.98304573	0.99946222	0.99795149
glcm_InverseVariance	0.99783829	0.98143622	0.99945029	0.99754584
glcm_DifferenceVariance	0.99659893	0.97714962	0.99846232	0.99813253
glcm_Idn	0.970345	0.9541169	0.99815628	0.98941716
glcm_Idm	0.99727834	0.98155574	0.99927382	0.99750192
glcm_Correlation	0.93000815	0.91942547	0.98138967	0.98861346
glcm_Autocorrelation	0.99882875	0.99071159	0.99869595	0.999001
glcm_SumEntropy	0.99415534	0.96610084	0.99671268	0.9937416
glcm_MCC	0.93302938	0.91561808	0.92970785	0.94261298
glcm_SumSquares	0.99716854	0.97213991	0.99724918	0.99249056
glcm_ClusterProminence	0.98931893	0.96491762	0.99077845	0.98964681
glcm_Imc2	0.97815659	0.92212229	0.97592009	0.9396985
glcm_Imc1	0.99777137	0.97086093	0.99426633	0.99035433
glcm_DifferenceAverage	0.99848775	0.98296153	0.99937115	0.99702514
glcm_Id	0.99726682	0.98139837	0.99930241	0.99748562
glcm_ClusterTendency	0.99642325	0.9699074	0.99671296	0.99142976
firstorder_InterquartileRange	0.99521994	0.97486054	0.99841554	0.99669589
firstorder_Uniformity	0.99336279	0.96426864	0.99280367	0.99113126
firstorder_Median	0.99824873	0.9849003	0.9999125	0.99978288
firstorder_Energy	0.99285535	0.95932458	0.99901088	0.92817079
firstorder_RobustMeanAbsoluteDeviation	0.99779342	0.97649493	0.99844399	0.99691045
firstorder_MeanAbsoluteDeviation	0.99844334	0.97694624	0.99821731	0.99604297
firstorder_TotalEnergy	0.99285535	0.95932458	0.99901088	0.92817079
firstorder_RootMeanSquared	0.99884074	0.98651296	0.99989506	0.9997983
firstorder_90Percentile	0.99935775	0.99013933	0.99995552	0.99994976
firstorder_Minimum	0.9698354	0.92185515	0.92678063	0.8563204
firstorder_Entropy	0.99424781	0.96641727	0.99700328	0.99412676
firstorder_Variance	0.99606424	0.97099704	0.99579973	0.98995292
firstorder_10Percentile	0.99778937	0.97865487	0.99899933	0.99627425
firstorder_Kurtosis	0.93847516	0.90984293	0.95408877	0.97436669
firstorder_Mean	0.99880578	0.98622651	0.99989669	0.99974019
glrlm_GrayLevelVariance	0.9958799	0.970434	0.99549048	0.98993049
glrlm_GrayLevelNonUniformityNormalized	0.99543004	0.96647932	0.99413525	0.99112422
glrlm_RunVariance	0.99473224	0.9734017	0.99380636	0.99548573
glrlm_GrayLevelNonUniformity	0.99949804	0.9674928	0.99614749	0.90009412
glrlm_LongRunEmphasis	0.99714509	0.97728321	0.99490007	0.99758722
glrlm_ShortRunHighGrayLevelEmphasis	0.99887822	0.9896169	0.99863031	0.99910028
glrlm_ShortRunEmphasis	0.99850478	0.9822178	0.99846149	0.99790335
glrlm_LongRunHighGrayLevelEmphasis	0.99248684	0.9784732	0.99830025	0.99622993
glrlm_RunPercentage	0.99663584	0.97947032	0.99853888	0.99734065
glrlm_RunEntropy	0.99144107	0.95450254	0.99513693	0.98727089
glrlm_HighGrayLevelRunEmphasis	0.99875561	0.98967427	0.99862958	0.99894877
glrlm_RunLengthNonUniformityNormalized	0.99765911	0.98130563	0.99893779	0.99778324
glszm_GrayLevelVariance	0.9664123	0.93487499	0.97426499	0.97948862
glszm_ZoneVariance	0.99376813	0.97889386	0.98567831	0.91246949
glszm_GrayLevelNonUniformityNormalized	0.97487458	0.93998164	0.99321227	0.98660887
glszm_SizeZoneNonUniformityNormalized	0.96600087	0.93446205	0.99453147	0.97741752
glszm_SizeZoneNonUniformity	0.97920816	0.92353173	0.99747104	0.77391648
glszm_LargeAreaEmphasis	0.99376008	0.97890241	0.98569335	0.91286785
glszm_SmallAreaHighGrayLevelEmphasis	0.99850607	0.98619218	0.9981323	0.99889261
glszm_ZonePercentage	0.99601524	0.97623238	0.99938994	0.99740069
glszm_LargeAreaLowGrayLevelEmphasis	0.95717008	0.96966115	0.94344936	0.94615198
glszm_HighGrayLevelZoneEmphasis	0.99826972	0.98491803	0.99815622	0.99876604
glszm_SmallAreaEmphasis	0.96165228	0.93147704	0.99401327	0.9749973
glszm_ZoneEntropy	0.95503524	0.92776222	0.96164933	0.96298698
ngtdm_Complexity	0.97512148	0.95607862	0.97876807	0.98862786
ngtdm_Contrast	0.99332362	0.9725499	0.99853608	0.99306747
ngtdm_Busyness	0.99582432	0.93702781	0.96571588	0.9623215

T2 map acquisition robust and reproducible features as defined by CCC & DR ≥ 0.9 and inter-/intra-ICC ≥ 0.75. CCC, concordance correlation coefficient; DR, dynamic range; firstorder, first-order features; GLCM, gray level co-occurrence matrix; GLDM, gray level difference matrix; GLRLM, gray level run length matrix; GLSZM, gray level size zone matrix; ICC, intraclass correlation coefficient; NGTDM, neighboring gray tone difference matrix. https://pyradiomics.readthedocs.io^19^.

**Table 2:** Robust and reproducible features across all sequences.

	Features	Shape maximum 3D diameter	Shape major axis length	Shape elongation	Shape maximum 2D diameter slice	Shape minoraxis length	Shape maximum 2D diameter column	Shape maximum 2D diameter row	glcm Imc1
T2w TSE	CCC	1.00	0.99	0.96	1.00	0.99	1.00	1.00	0.99
	DR	0.98	0.98	0.95	0.99	0.96	0.98	0.98	0.97
	Intra-observer ICC	1.00	0.99	1.00	1.00	0.99	1.00	1.00	1.00
	Inter-observer ICC	0.99	1.00	1.00	1.00	1.00	0.99	0.99	0.99
T1w TSE	CCC	1.00	0.98	0.99	1.00	0.98	1.00	0.99	0.93
	DR	0.97	0.95	0.95	0.98	0.96	0.97	0.97	0.91
	Intra-observer ICC	1.00	1.00	1.00	1.00	1.00	1.00	1.00	1.00
	Inter-observer ICC	0.99	0.99	0.99	1.00	0.99	0.98	0.98	0.97
FLAIR	CCC	1.00	1.00	0.98	1.00	0.99	1.00	0.99	0.97
	DR	0.97	0.96	0.96	0.99	0.96	0.97	0.95	0.94
	Intra-observer ICC	1.00	0.99	0.99	1.00	0.99	1.00	0.99	0.99
	Inter-observer ICC	0.98	0.98	0.99	1.00	0.98	0.98	0.97	0.96
T2 map	CCC	0.99	1.00	0.92	0.99	0.99	0.99	0.99	1.00
	DR	0.96	0.98	0.93	0.98	0.97	0.96	0.95	0.97
	Intra-observer ICC	1.00	1.00	0.99	1.00	1.00	0.99	0.99	0.99
	Inter-observer ICC	0.99	1.00	0.99	0.99	1.00	0.99	0.98	0.99
HASTE	CCC	1.00	0.99	0.93	1.00	0.99	0.99	0.99	0.98
	DR	0.98	0.97	0.93	0.99	0.96	0.96	0.97	0.96
	Intra-observer ICC	1.00	0.99	0.99	1.00	0.99	0.99	0.99	0.99
	Inter-observer ICC	0.99	0.99	0.99	1.00	0.99	0.99	0.99	0.99

Across all sequences, eight features proofed to be robust and reproducible. Except of Imc1 from the GLCM feature class, all other features were shape features. *CCC* concordance correlation coefficient, *DR* dynamic range, *FLAIR* fluid-attenuated inversion recovery, *GLCM* gray level co-occurrence matrix, *HASTE* half-Fourier acquisition single-shot turbo spin-echo, *ICC* intraclass correlation coefficient, *T1w* T1-weighted, *T2w* T2-weighted, *TSE* turbo spin-echo.

### T2 map has superior discriminative power for non-shape features

**Figure 4:**
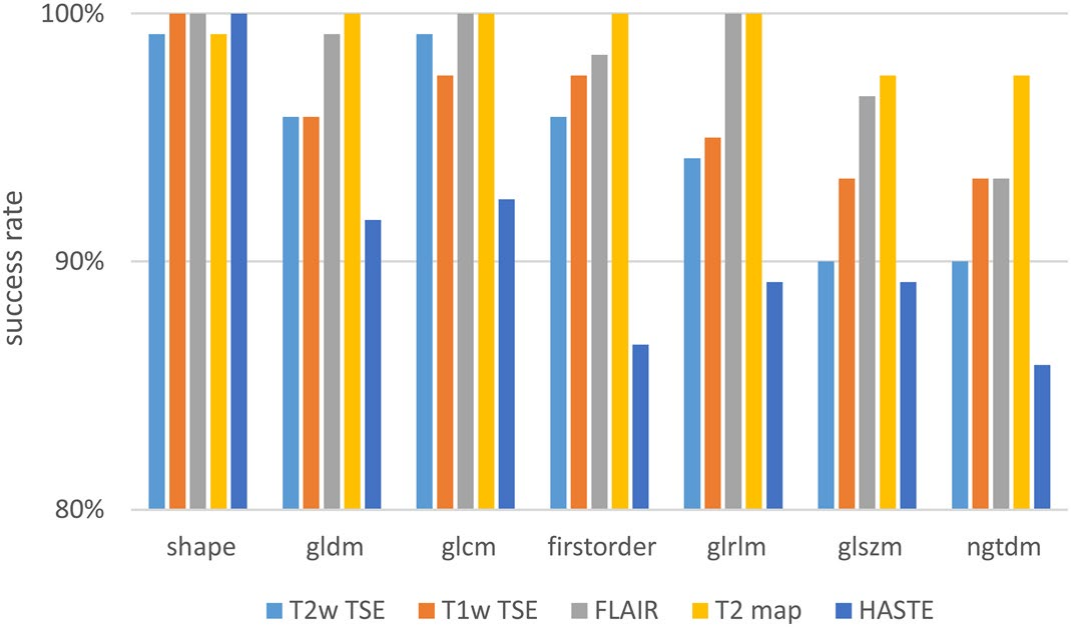
Success rates of MRI sequences within individual feature classes. Maximum score of 120 successes to differentiate a total of 120 pairs of fruits equals a success rate of 100%. firstorder, first-order features; GLCM, gray level co-occurrence matrix; GLDM, gray level difference matrix; GLRLM, gray level run length matrix; GLSZM, gray level size zone matrix; NGTDM, neighboring gray tone difference matrix.

The statistical significance of the perfect results of Gini score one was p_Gini_ = 6! 6! / 12! = 1/924 ≈ 1.08E−3. In total, we computed 63,600 Gini scores for the differentiation of 120 pairs of fruits by 106 features of five sequences. We computed the Gini score for the differentiation of individual fruits. All five sequences yielded a maximal score of 120 successes. Note that, with 106 tested features the false discovery rate (FDR) of a single success, FDR = 1-(1 − p_Gini_)^106^ ≈ 10.8%, was rather high. The significance of 120 successes, however, was significantly high, p_120_ < 1e−58, and demonstrated the predictive power of each of the five sequences. To study the predictive power of the classes, we counted their number of successes. Beside class ngtdm all classes yielded a maximal score of 120 successes. Class ngtdm failed only for the pair Kiwi3/Kiwi4. The high success score demonstrated the predictive power for each class on its own. Figure 4 shows the success rate of the sequences within individual classes. Within shape, three sequences, T1 TSE, FLAIR, and HASTE, yielded a maximal score of 120 successes (100%). T2 map and T2 TSE failed for pair Kiwi2/Kiwi3 and pair Kiwi1/Kiwi2, respectively. For three classes, gldm, glcm, and first order, T2 map yielded the maximal score of 120 successes (100%). T2 map revealed also the top result of 117 successes (97.5%) within glszm and ngtdm. The majority of failed tests occurred for pairs of identical fruit types. Within classes shape, glrlm, and gldm, all sequences obtained a 100% success rate for pairs of different fruit types. For pairs of different fruit types, success rates below 100% were exceptions (n = 4 out of 35), and the minimal success rate was 95.8% for T2 TSE in class glszm. The differentiation of fruits of identical type was more difficult. Success rates below 100% were the rule (n = 26 out of 35), and the minimal success rate was 33.3% for HASTE in class glszm.

### Shape feature class and T2 map imaging technique for non-shape features yield highest discriminative performance

Some fruits were easily distinguishable by differences in size, shape, and textures. Discriminations between apples and limes had high accuracy, e.g., 478 out of 5 × 106 = 530 features were able to distinguish between apple3 and lime2. Discriminations between two fruits of identical type were more challenging, e.g., only 64 out of 5 × 106 = 530 features were able to distinguish between kiwi1 and kiwi3. For fruits of identical type, the mean number of successful features was 155 ± 50 out of 530 to be compared with the mean number 386 ± 54 out of 530 for fruits of different type. A feature with perfect sensitivity and robustness would provide optimal predictive power for each of the 120 pairwise differentiations of the sixteen fruits. Table 3 shows the top-ranked features (17 features for a cut-off value of 109 successes, see supplementary Table 2 (Table D.1) for all features and supplementary Fig. 3 (Fig. E.1) for the respective Bland Altmann plots of the top ranked features). We observe an optimal score of 120 correct discriminations only for one feature, Maximum2DDiameterSlice (class shape) of HASTE. Also, for other sequences, Maximum2DDiameterSlice achieved high ranks, two (T2 TSE, T2 map), 6 (FLAIR), and eleven (T1 TSE), among the 5 × 106 = 530 features. Among the 17 best-ranked features, eleven features are of class shape. Of the six non-shape top features five were of T2 map. Features of class shape were enriched in the set of top-ranked features, and T2 map imaging technique was enriched in the top ranked non-shape features.

**Table 3:** Top ranked features by number of pairwise discriminative successes based on Gini score analysis.

Rank	Feature	Sequence	Class	No. successes	Rate (%)
1	Maximum2DDiameterSlice	HASTE	Shape	120	100.0
2	Maximum2DDiameterSlice	T2w TSE	Shape	114	95.0
2	Maximum2DDiameterSlice	T2 map	Shape	114	95.0
4	MajorAxisLength	HASTE	Shape	112	93.3
5	DependenceNonUniformityNormalized	T2 map	Gldm	111	92.5
6	Maximum3DDiameter	T2w TSE	Shape	110	91.7
6	Maximum2DDiameterRow	T2w TSE	Shape	110	91.7
6	Maximum2DDiameterSlice	FLAIR	Shape	110	91.7
6	Maximum2DDiameterRow	FLAIR	Shape	110	91.7
6	Median	T2 map	Firstorder	110	91.7
11	MajorAxisLength	T2 map	Shape	109	90.8
11	RunPercentage	T2 map	glrlm	109	90.8
11	Maximum2DDiameterSlice	T1w TSE	Shape	109	90.8
11	Idm	T2 map	glcm	109	90.8
11	RunPercentage	FLAIR	glrlm	109	90.8
11	Id	T2 map	glcm	109	90.8
11	Maximum2DDiameterColumn	T2w TSE	Shape	109	90.8

The 17 top ranked features up to a cut-off value of 109 successes are depicted to pairwise discriminate variant fruits. See supplementary Table 2 (Table D.1) for all features.

## Discussion

Radiomics is increasingly applied to perform data mining and augment image data for model building^1^. Nevertheless, data on the robustness and reproducibility of radiomic features, especially for MRI radiomics, are scarce and remain controversial^5,9,16–18,20,21^. Monocenter as well as multicenter studies dealing with the robustness and reproducibility of radiomic features obtained controversial results^5,9,16–18^. Baeßler et al. have demonstrated high vulnerability of the majority of radiomic features^9^. We applied a phantom model as proposed by Baeßler et al.^9^ to acquire standard clinical routine (HASTE, T2w TSE, FLAIR, T1w TSE) and further experimental (T2 map) imaging techniques and extracted 106 radiomic features per sequence. In accordance with Baeßler et al.^9^, we analyzed intra- and inter-observer reproducibility as well as robustness of radiomics features. We could reveal superiority of T2 map yielding the highest performance. FLAIR was the second best imaging sequence. We could demonstrate robustness and reproducibility of 84 features applying T2 map, 59 features applying FLAIR, and only a subset of eight features was robust and reproducible across all sequences. The highest discriminative performance was found for feature class shape and for the imaging technique T2 map for non-shape features.

Baeßler et al. have proposed a subset of 15 features as reliable candidates for radiomic signatures within clinical studies^9^. They claim that all other features should be favored to be dismissed during the feature selection process to improve validity of model building^9^. We examined approximately twice as much features (106 vs 45) and could reveal approximately half as much stable features (8 vs 15)^9^. In line with Baeßler et al., our subset of eight robust features across all sequences included shape features and a feature of the GLCM feature class^9^. Nevertheless, we could not corroborate the feature set of Baeßler et al.^9^. All of our top robust features were variant to the proposed 15 features^9^. We could demonstrate that subsets of the proposed 15 robust features were transferrable to specific MRI sequences in our data set^9^. Our analyzes emphasize that one may not overstate generalizability of single center datasets^9,22^. Mapping imaging techniques enable acquisition of quantitative imaging data in contrast to the standard qualitative MR images^23^. Though mapping parameters depend on the applied field strength, they inherit the potential to serve as quantitative biomarkers^23^. We could demonstrate that T2 map has the highest potential for robust and reproducible feature extraction. In line with Baeßler et al., intra-observer variance revealed a high stability^9^. We applied a semi-automatic segmentation process which is known to reduce inter-observer variance^12^. Nevertheless, inter-observer variance remained a dominant factor reducing the amount of robust and reproducible imaging features. Multidimensional feature classes are routinely applied in radiomic research to mine data and build specific models^1^. Our study urges caution in the interpretation of radiomics study results, especially when the possibility of rapid translation into clinical routine is proclaimed^8^. We elucidate the potential of shape features to represent the most promising features. This may be interpreted in line with a recent proof of validity study of Welch et al.^6^. Welch et al. have been able to demonstrate that radiomic signature features may be surrogates of shape features only and may not yield additional pertinent for prognostication^6^. High-dimensional features may be redundant, and predictive power may be based on shape features^6^. The study has been performed employing computed tomography (CT) data^6^. Qualitative MRI data may inherit an even higher vulnerability.

Our study suffers from limitations that warrant discussion. We applied a fruit phantom and a standardized phantom of defined multi-material compositions might have led to higher levels of reproducibility. To stay in line with Baeßler et al. we favored application of a multifruit model^9^. Stationary macro-object phantoms have limited comparability to human tissues and direct translation to in-vivo radiomic studies would overestimate the findings and is beyond the scope of our study. In an in-vivo setting, MRI sequences are prone to motion artefacts which might have altered the results. We acquired our scan and rescan data directly one after the other on one 3 T scanner. We cannot rule out that recalibration of the MR scanner, temporal or geographical variation might have altered the results^22^. We did design our study to acquire two measurement sets in form of test and retest data, and more repetitions could have stabilized the results. Contrary to Baeßler et al., we applied PyRadiomics to perform the feature extraction^9,19,24,25^. PyRadiomics promotes transparent multicenter research with open source codes being available^8,19^. In an in-vivo setting or a standard of care clinical scenario, the limitations described above would lead to increased variation in the VOI-definition of repeat or follow-up scans, which in turn increases the variation of all radiomic features. One would expect to see a decrease in the number of robust features across all sequences. This highlights the importance of stratifying specific robust and reproducible sequences and corresponding feature subsets to path the way for clinical translation of radiomic data augmentation in the future.

In conclusion, we provide further evidence that the robustness of MRI radiomics features depends on the particular MRI sequence used. We revealed superiority of T2 map to lead to the highest amount of robust and reproducible quantitative imaging features as well having the highest discriminative performance. FLAIR was the second best sequence. Only eight out of 106 features were stable across all MR sequences, and seven out of the respective eight features were part of the shape feature class. We could not corroborate the subset of robust features of Baeßler et al. and therefore urge caution in interpreting radiomic research^8,9^. We propose the inclusion of mapping imaging techniques in the clinical routine setting to enable acquisition of robust imaging data pathing the way for multicenter multivendor research. Multicenter multivendor validation studies employing phantoms and in-vivo experiments are needed prior to translation of radiomic findings and respective models into clinical routine.

## Methods

### Study design

We constructed a multi-fruit phantom as proposed by Baeßler et al.^9^ consisting of four onions, four limes, four kiwifruits and four apples. Image acquisition was performed as scan-rescan: repositioning in the same direction with replanning of all sequences, two measurements.

### MR imaging acquisition and examination

**Figure 5:**
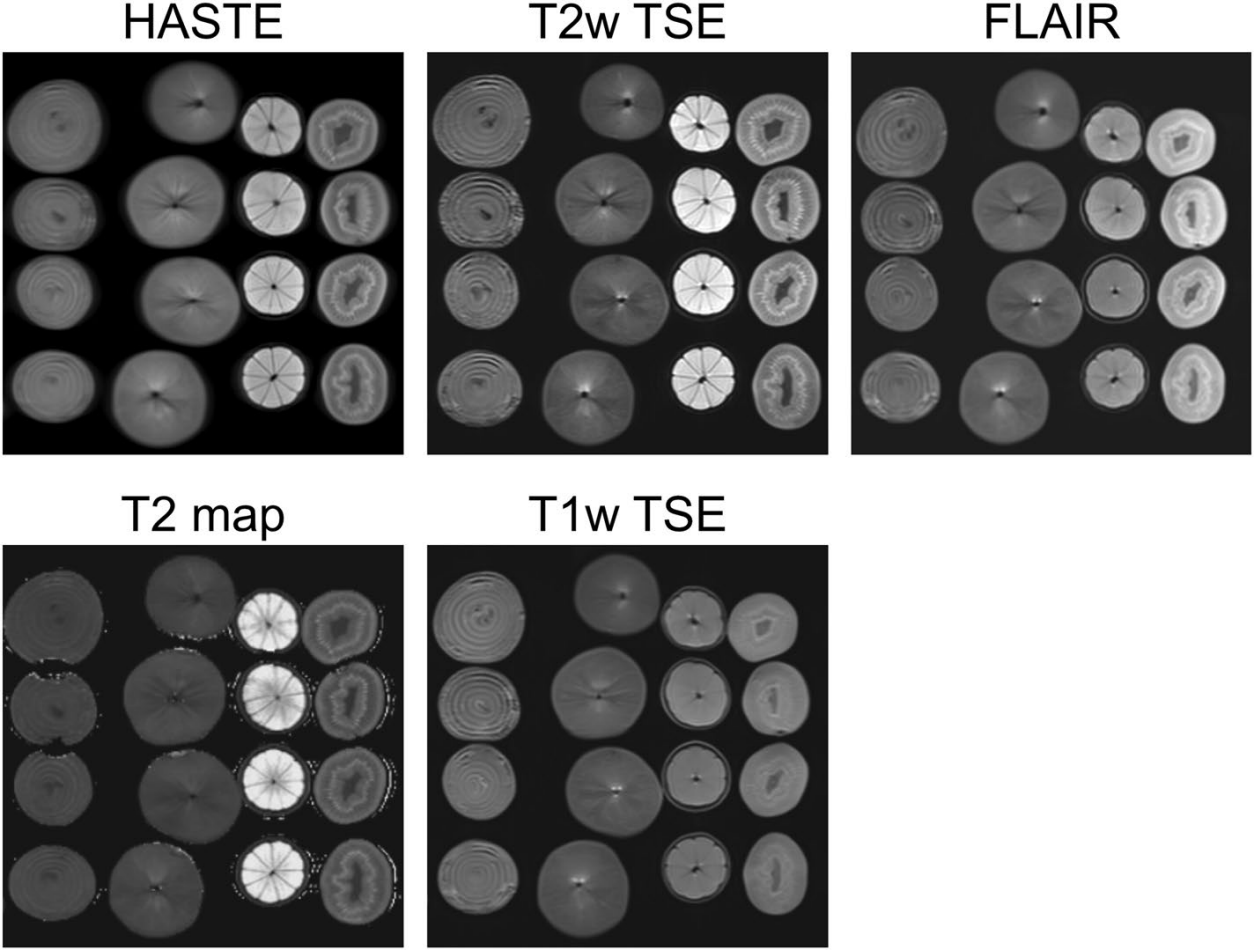
Representative images of the acquired magnetic resonance imaging sequences. FLAIR, fluid-attenuated inversion recovery; HASTE, half-Fourier acquisition single-shot turbo spin-echo; T1w, T1-weighted; T2w, T2-weighted; TSE, turbo-spin-echo.

All examinations were performed on a single 3 T scanner with a standard body-array coil (Magnetom Prisma^FIT^, Siemens Healthcare, Erlangen, Germany) and built-in spine phased-array coil**.** The sequences were adapted from the standard clinical liver sequences, including an experimental quantitative mapping imaging technique leading to a total of five variant imaging techniques: (I) T2-weighted (T2w) half-Fourier acquisition single-shot turbo spin-echo (HASTE), (II) T2w turbo spin-echo (TSE), (III) T2w fluid-attenuated inversion recovery (FLAIR), (IV) T2 map and (V) T1-weighted (T1w) TSE (Fig. 5). Details of imaging parameters of acquisition are shown in Table 4.

**Table 4:** Magnetic resonance imaging sequence parameters.

Sequence	T2w HASTE	T2w TSE	T2w FLAIR	T2 Map	T1w TSE
Orientation	Axial	Axial	Axial	Axial	Axial
TR (ms)	1000	7500	9000	4000	600
TE (ms)	87	96	89	34; 80	20
Averages	1	2	1	1	2
Flip angle	115	160	150	180	161
FOV (mm^2^)	382 × 350	400 × 400	400 × 400	299 × 399	400 × 400
Matrix (px^2^)	280 × 256	256 × 320	256 × 256	173 × 384	240 × 320
Bandwidth (Hz)	700	200	220	220	185
Slice thickness (mm)	6	3	5	4	4
Original protocol	Liver	Liver	Liver	Liver	Liver

Acquisition parameters of the modified clinical routine protocols are shown. *FLAIR* fluid-attenuated inversion recovery, *FOV* field of view, *HASTE* half-Fourier acquisition single-shot turbo spin-echo, *T1w* T1-weighted, *T2w* T2-weighted, *TE* echo time, *TR* repetition time, *TSE* turbo-spin-echo.

### Image preprocessing and segmentation

**Figure 6:**
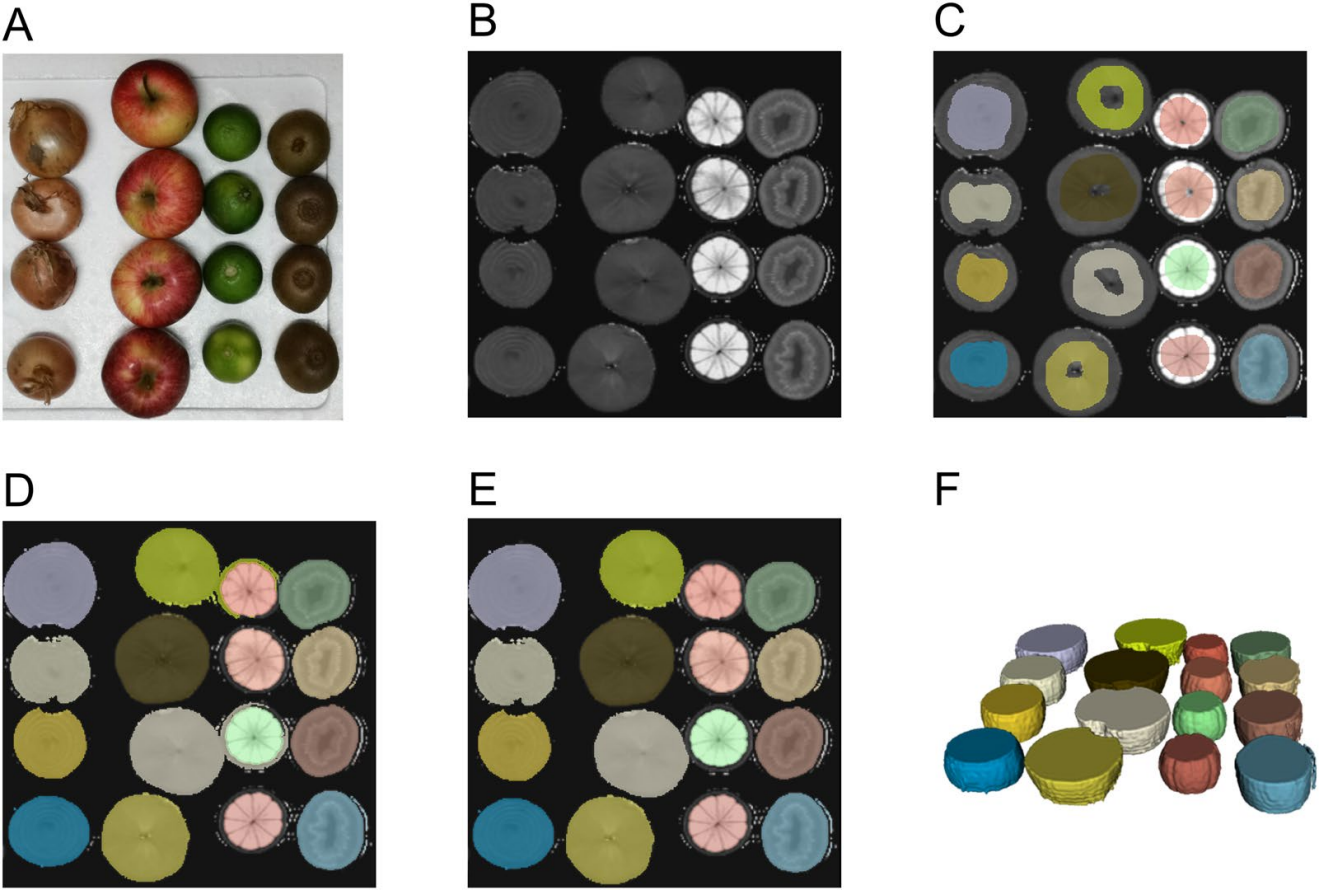
Phantom design and workflow of semi-automatic segmentation. The phantom (**A**) and the workflow of semi-automatic segmentation are shown exemplarily for T2-weighted turbo-spin-echo acquisition (**B**–**E**). On the original image (**B**), we manually defined preliminary volumes of interest (**C**, diameter 1.5 cm). The growth from seeds algorithm was used to augment the 3D volumes (**D**) with subsequent manual correction of erroneous border segment sections (**E**). In F a representative 3D volume rendering is shown.

MR images were extracted in Digital Imaging and Communications in Medicine (DICOM) format and imported into the open-source 3D Slicer software platform (http://slicer.org, version 4.9.0)^24,25^. Images were resampled to a spacing of 1 mm × 1 mm × 1 mm employing B-spline interpolation (https://www.slicer.org/wiki/Registration:Resampling, supplementary methods 2 of Griethuysen et al.^19^)^25^. For the segmentation, a three-dimensional volume of interest (VOI) was defined in each fruit employing the paint tool of the segment editor^25^. Augmentation of the VOI to match the boundaries of the fruit was performed using the semi-automatic grow from seeds algorithm, known to reduce inter-observer variability^12,25,26^. By limiting manual VOI placement to the middle proportion of each fruit with a 1.5 cm diameter VOI, we did limit the consecutive growing algorithm to segment the middle portion of each fruit, thus reducing partial volume artefacts of the upper and lower boarder zones (Fig. 6). Fruits were positioned in close proximity paralleling the real world scenario of VOIs being surrounded by variant tissues. Consequently, segmentation errors were observed. Respective foci of error were manually corrected employing the brush-erase tool^9^. The segmentation workflow is shown in Fig. 6. To analyze the inter- and intra-observer variance, the segmentation workflow as well as feature extraction was additionally conducted by a second reader after initial training and by the first reader after a pause of one month, respectively.

### Features extraction

We applied the open-source package PyRadiomics^19^ as extension within the 3D Slicer software platform^24,25^ to extract the radiomic features. Feature definitions of PyRadiomics are broadly implemented according to the IBSI definition consensus^8,11,19^. From seven feature classes we extracted all original standard features: Shape-based, First Order Statistics, Gray Level Co-occurrence Matrix (GLCM), Gray Level Run Length Matrix (GLRLM), Gray Level Size Zone Matrix (GLSZM), Gray Level Dependence Matrix (GLDM), Neighboring Gray Tone Difference Matrix (NGTDM) leading to 107 features per VOI and sequence (http://pyradiomics.readthedocs.io^19^). Default settings of PyRadiomics were used for feature extraction, i.e. original without filtering, no wavelet-based features, bin width 25, and enforced symmetrical GLCM, http://pyradiomics.readthedocs.io^3,8,19^. As we restricted the segmentation to the middle proportion of the fruits, least axis parameter was systematically biased and we excluded this feature from the analyses, leading to a total of 106 “*true*” features per VOI and sequence (further referred to as the total amount of features).

### Evaluation of robustness and reproducibility

To ensure highest methodological transparency, we used open-source software with source codes being available online. We performed statistical calculations and analysis with Python 3.7.6^27^, within Jupyter Notebook^28^ with the respective package scipy (version 1.4.1)^29^. We computed concordance correlation coefficient (CCC) and dynamic range (DR) values on paired samples, x and y^30–32^. The samples x and y contained a first set and a second disjunct set of values of a feature, respectively. CCC values range from -1 to 1, where 1 refers to the perfect agreement between the two samples x_i_ = y_i_, i = 1, …, n. The value CCC = -1 refers to perfectly anticorrelated pairs of samples x_i_ = -y_i_, i = 1, …, n. The value DR = 0 refers to the lowest possible variability in the sets x and y, i.e., x_1_ = x_2_ = $\cdots$ = x_n_ ≠ y_1_ = y_2_ = $\cdots$ = y_n_. The value DR = 1 refers to optimal reproducibility x_i_ = y_i_, i = 1, …, n combined with a nonzero data range. Recent studies have defined high correlation for CCC and DR using a cut-off value of 0.9^9,32^. The choice of 0.9 as the cut-off has been based on the study of Segal et al. applying Pearson correlation measurement^33^. CCC is known to outperform Pearson correlation coefficient^30^ and no consensus exists, therefore, we defined our cut-off value at 0.9, as proposed by Baeßler et al.^9^. Also, in line with Baeßler et al., we further added analyses of a relaxed and a more strict cut-off value of 0.85 and 0.95, respectively^9^. Further, we tested intra- and inter-observer reproducibility by means of intraclass correlation coefficients (ICCs)^34,35^. ICC assesses the reproducibility of measurements performed by different observers measuring the same quantity^34,35^. ICC range from − 1 to 1, where 1 refers to perfect correlation and − 1 refers to perfect anticorrelation. In accordance with Baeßler et al.^9^, we defined excellent, ≥ 0.75; good, 0.60–0.74; moderate, 0.40–0.59; and poor, ≤ 0.39, reproducibility^36,37^. To correct CCC values for subtle intrareader variances, we applied the bias correction as done by Baeßler et al.: CCC_corr_ = CCC + (1 − intra-observer ICC)^9^.

### Gini scores

We applied the Mann–Whitney U test^38^ to measure the predictive power of a feature to distinguish the sixteen individual objects of the phantom. The Mann–Whitney U test computes a U-parameter from the numeric ranks of the values in the union of two groups. The statistic of the U parameter describes the null hypothesis of identical distributions of both populations. We rescaled the U parameter to the area under the receiver operating characteristic curve (ROC, AUC)^39^

$${\text{AUC}} = {\text{U}}/\left( {{\text{n}}_{{\text{1}}} {\text{n}}_{{\text{2}}} } \right),$$

where n_1_ and n_2_ denote the number of feature values of fruit A and B, respectively. We calculated the Gini score

$${\text{Gini}} = {\text{2}}\;{\text{AUC}} - {\text{1}}$$

to measure the predictive power. Note that, a Gini Score of Gini = 100% enables a correct decision based on a single value of a feature. For features with a Gini score of Gini = 0%, any prediction would be random and the feature would give no valuable information for a decision. For an individual fruit, we considered six replicate values, one value of each of three segmentations of two scans. The Mann–Whitney U test compared the six values of a fruit with the six values of another fruit and computes a value of Gini score between zero (no predictive power) and one (perfect predictive power). We named the fruits apple1-4, lime1-4, onion1-4, and kiwi1-4. We denoted a group of features to be successful to distinguish a pair of fruits, if at last one feature in the group yielded a perfect Gini score of one. For example, a sequence yielded a maximal score of 120 successes only if for each of the 120 pairs of fruits, at least one of its 106 features was able to distinguish the two individual fruits.

### General statistical analysis

For statistical analysis, the values of significance are depicted in the graphs as followed: *p < 0.05; **p < 0.01; ***p < 0.001. Further graphical illustrations and statistics were performed employing JMP 14 (SAS), Prism 6.0 (GraphPad software), Microsoft Excel (Microsoft Corporation) and Affinity Designer 1.8.5.703 (Serif (Europe) Ltd).

## Supplementary Information

Supplementary Figure 1.

Supplementary Figure 2.

Supplementary Figure 3.

Supplementary Table 1.

Supplementary Table 2.

Supplementary Table Legends.

## Data Availability

The datasets used and/or analysed during the current study are available from the corresponding author on reasonable request.

## Abbreviations

AUC: Area under the curve
CCC: Concordance correlation coefficient
DICOM: Digital imaging and communications in medicine
DR: Dynamic range
FLAIR: Fluid-attenuated inversion recovery
FDR: False discovery rate
GLCM: Gray level co-occurrence matrix
GLDM: Gray level dependence matrix
GLRLM: Gray level run length matrix
GLSZM: Gray level size zone matrix
HASTE: Half-Fourier acquisition single-shot turbo spin-echo
IBSI: Image biomarker standardization initiative
ICC: Intraclass correlation coefficients
NGTDM: Neighboring gray tone difference matrix
ROC: Receiver operating characteristic
rrf: Robust and reproducible features
T1w: T1-weighted
T2w: T2-weighted
TSE: Turbo spin-echo
VOI: Volume of interest
