# Supplementary material for: Impact of rescanning and repositioning on radiomic features employing a multi-object phantom in magnetic resonance imaging
Source: Sci Rep. 2021 Jul 9;11:14248. doi: 10.1038/s41598-021-93756-x (PMC8271025; doi:10.1038/s41598-021-93756-x)
Supplement: Supplementary file 1 — Supplementary Figure 1. [file 41598_2021_93756_MOESM1_ESM.pdf]

Supplementary Figure 1: *Measurements of reproducibility and robustness for each feature and MRI sequence*

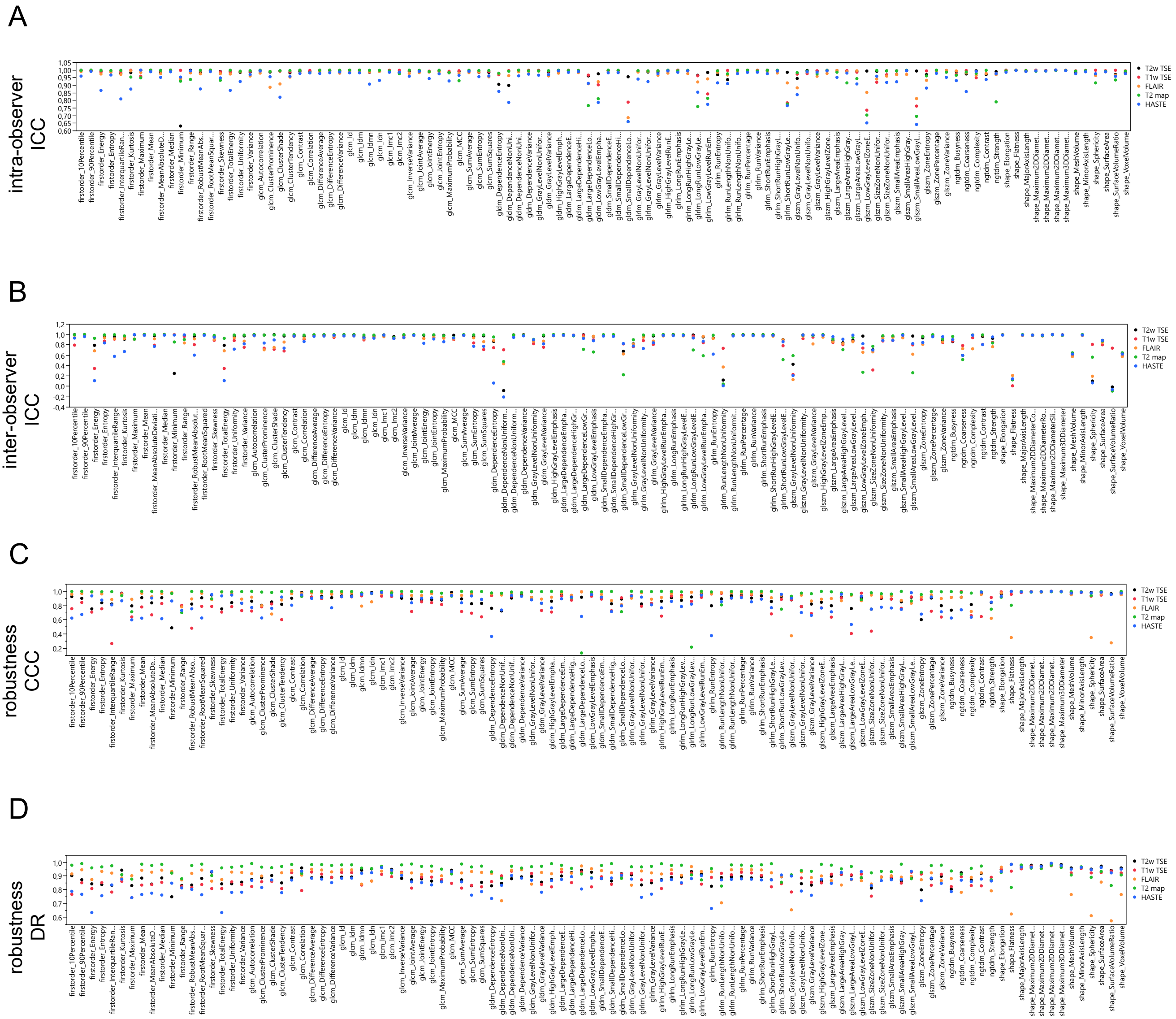

We depict comprehensive raw data of measurements of reproducibility (ICC, intraclass correlation coefficient) for intra- (A) and inter- (B) observer analysis and measurements of robustness (CCC, concordance correlation coefficient, C; DR, dynamic range, D). Each feature can be put into exact context of the measured values to estimate its validity. Colored dots depict the respective MRI sequence: T2w TSE, black; T1w TSE, red; FLAIR, orange; T2 map, green; HASTE, blue. Raw data values are in supplementary table 1.
