# Supplementary material for: Impact of rescanning and repositioning on radiomic features employing a multi-object phantom in magnetic resonance imaging
Source: Sci Rep. 2021 Jul 9;11:14248. doi: 10.1038/s41598-021-93756-x (PMC8271025; doi:10.1038/s41598-021-93756-x)

**Suppl. Figure 2:** Bland Altmann plots of features that were robust and reproducible across all sequences. Each colored point represents a fruit. The color code is green for lemon, red for apple, brown for kiwi, and sandybrown for onion. Plots are produced with the program gnuplot. Ideally, the points should be well-separated from each other. The differences, plotted on the y-axes, should show preferable small random fluctuations around zero.

Maximum3DDiameter


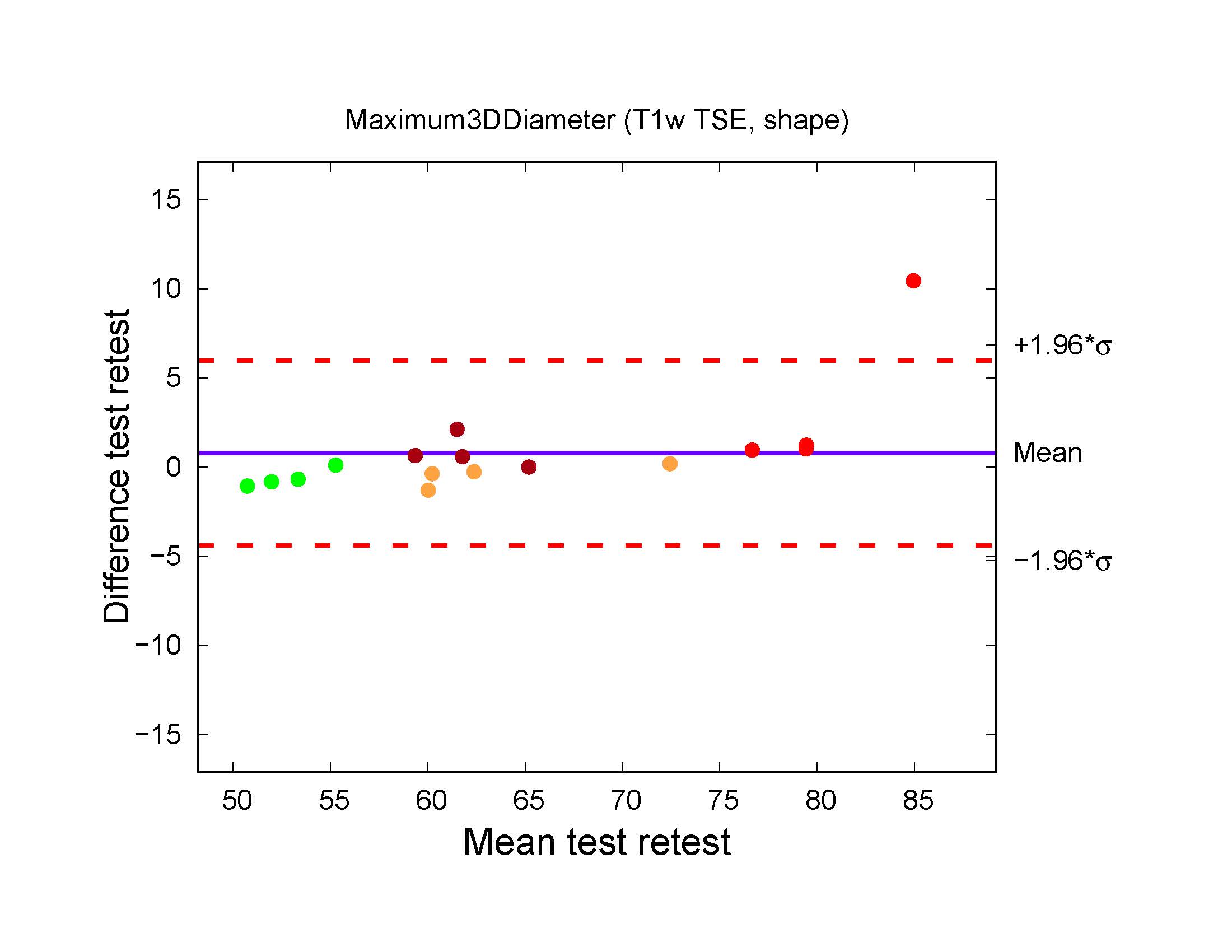

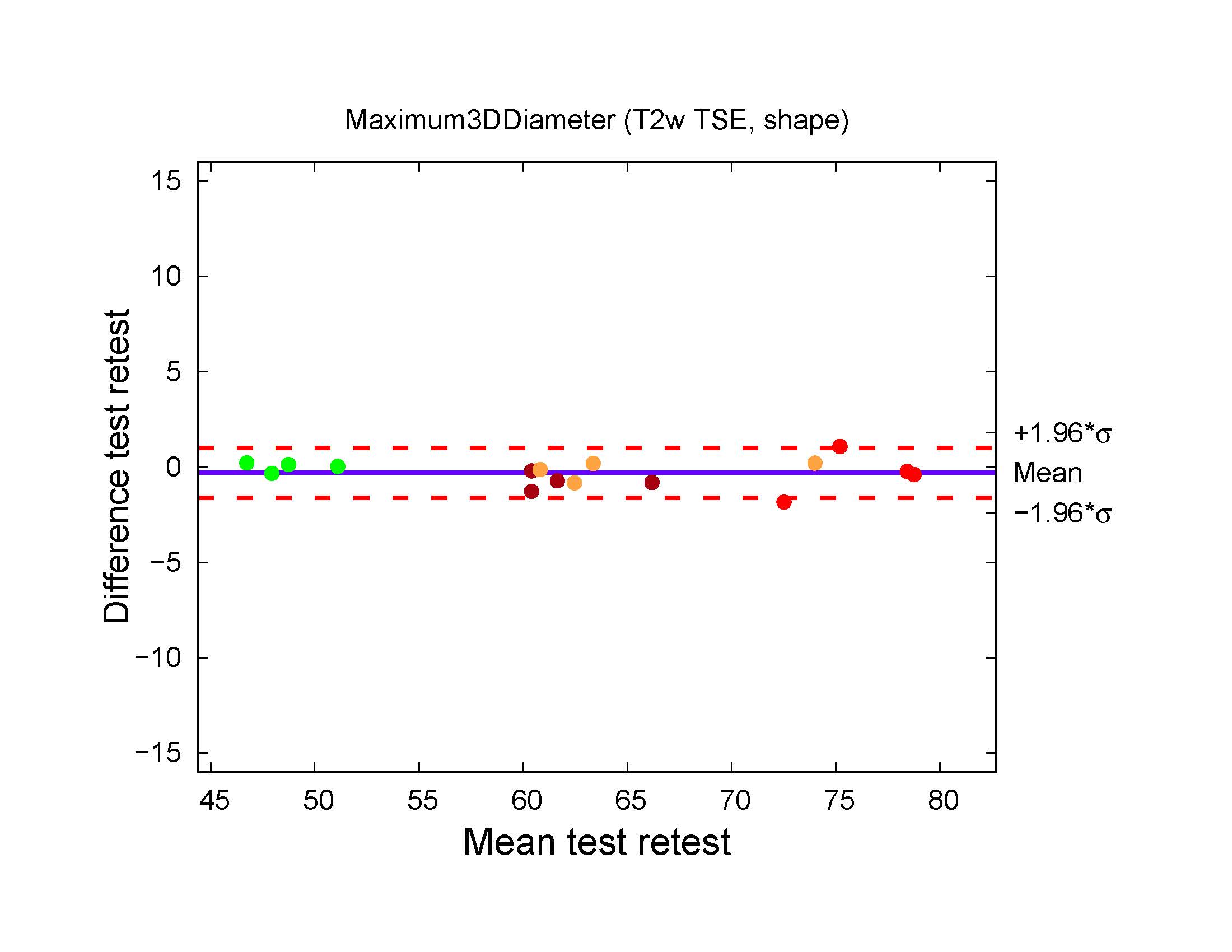

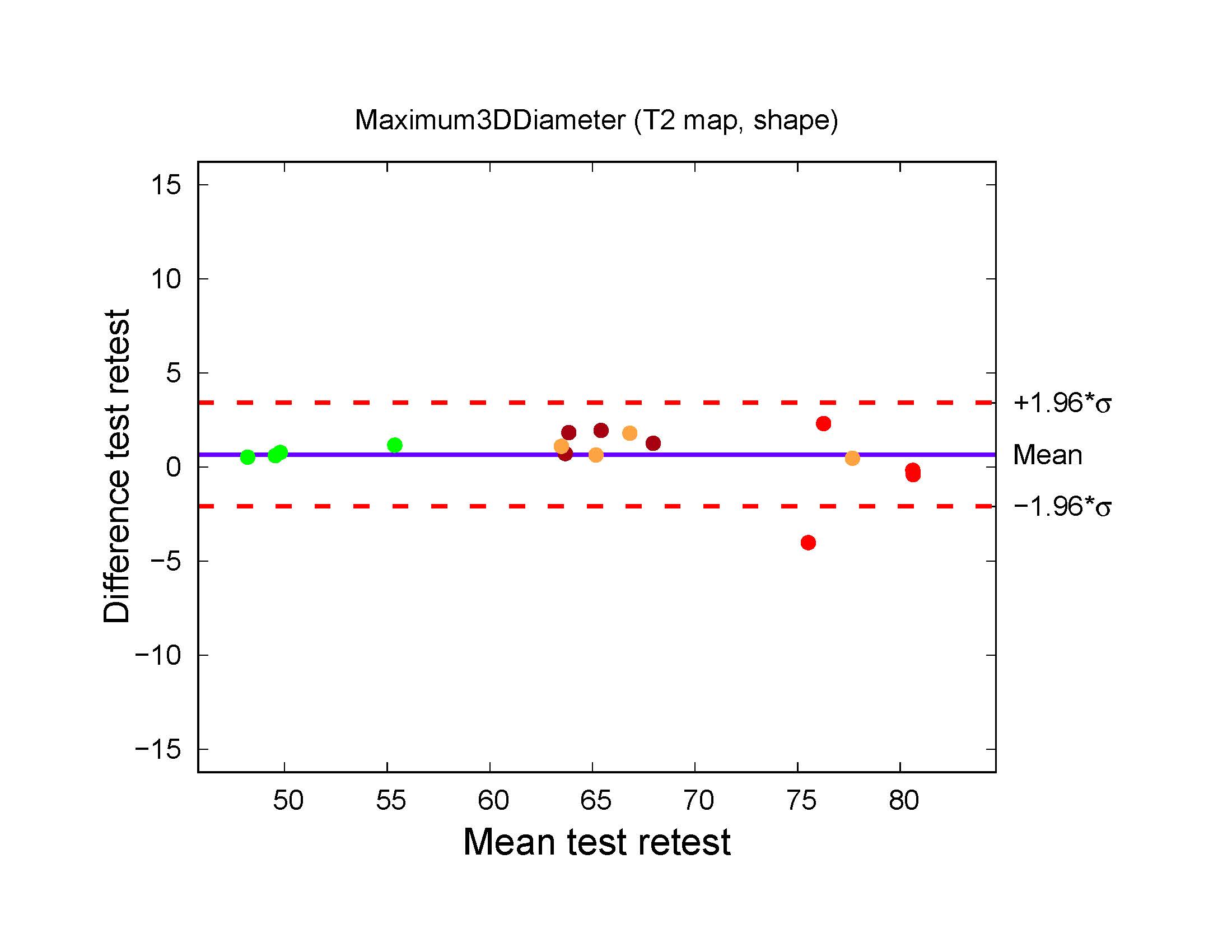

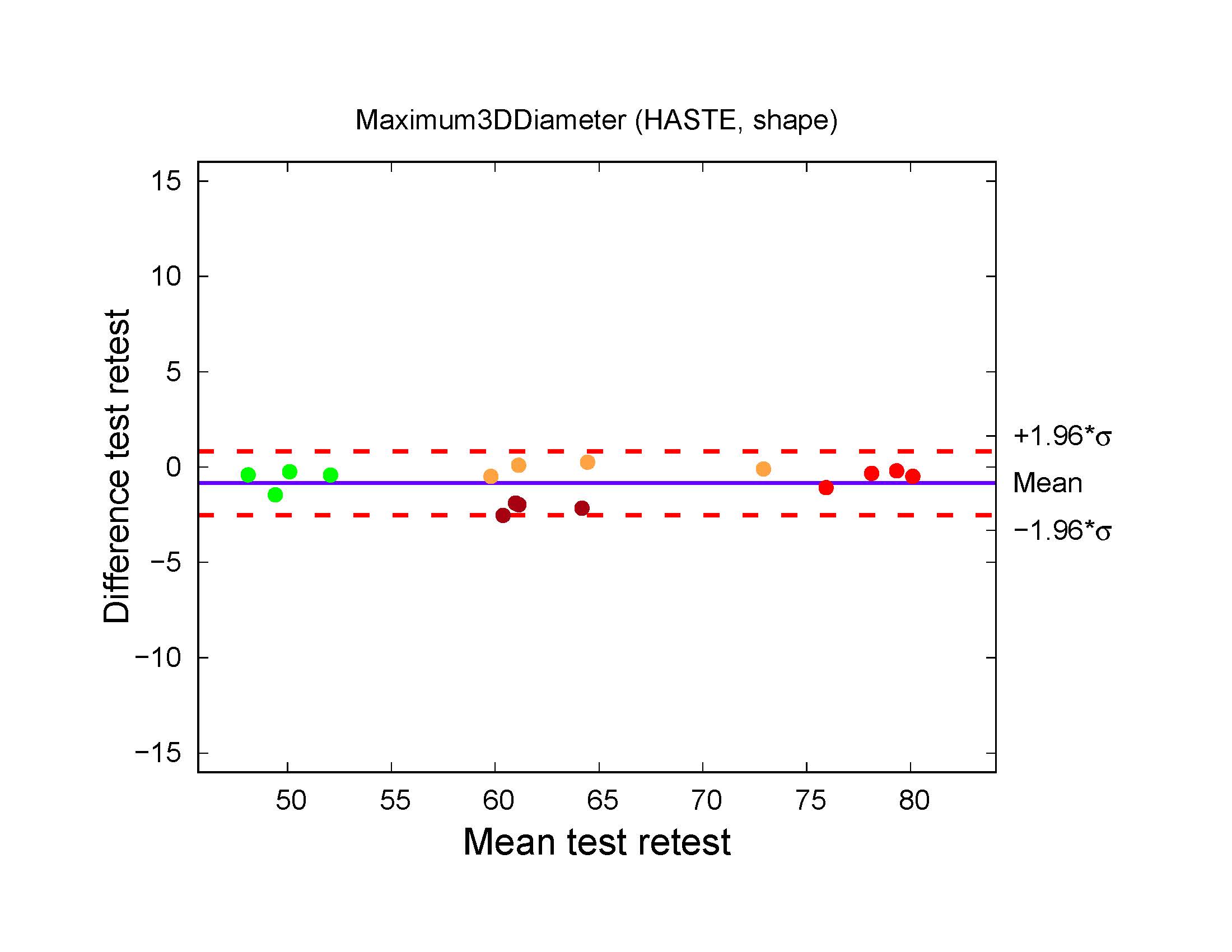

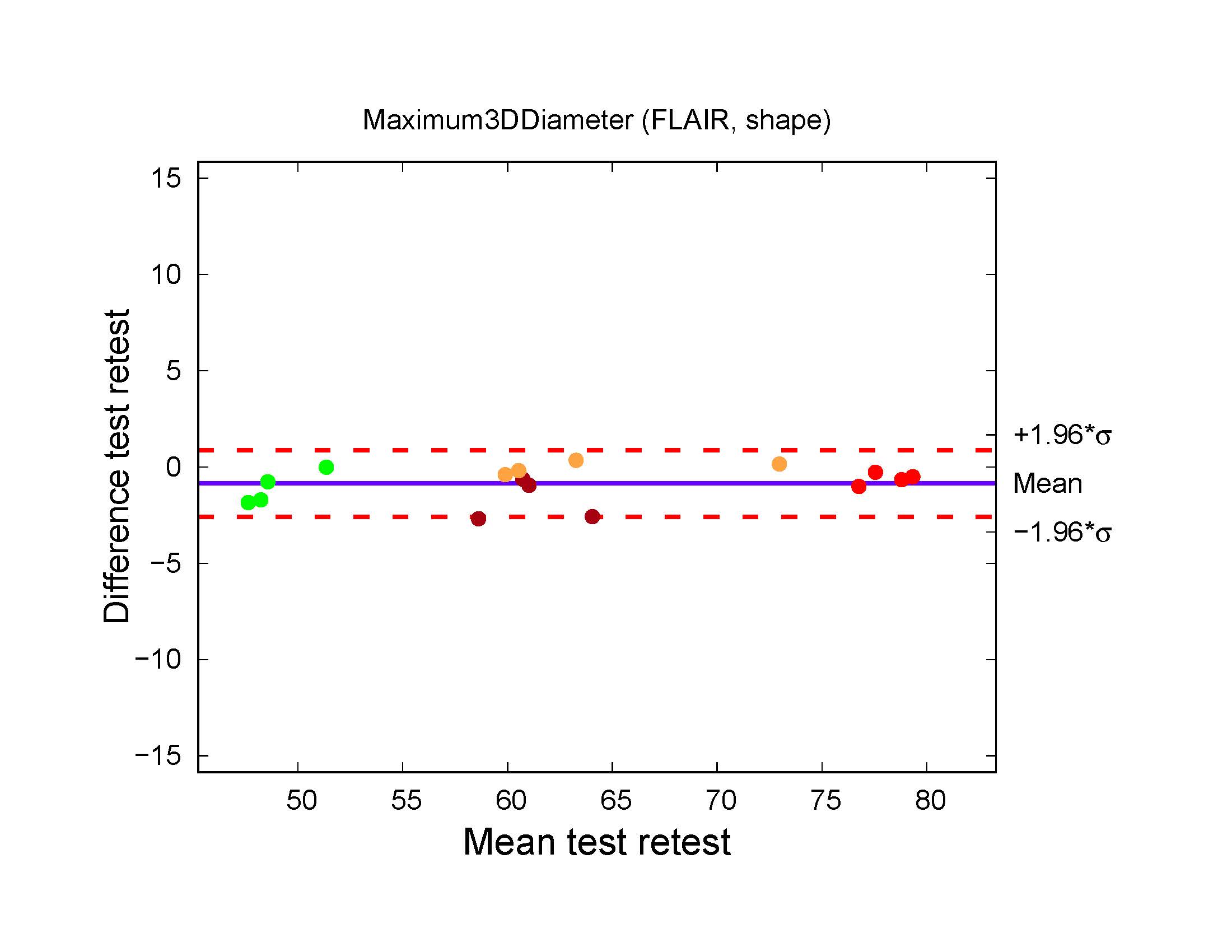


MajorAxisLength


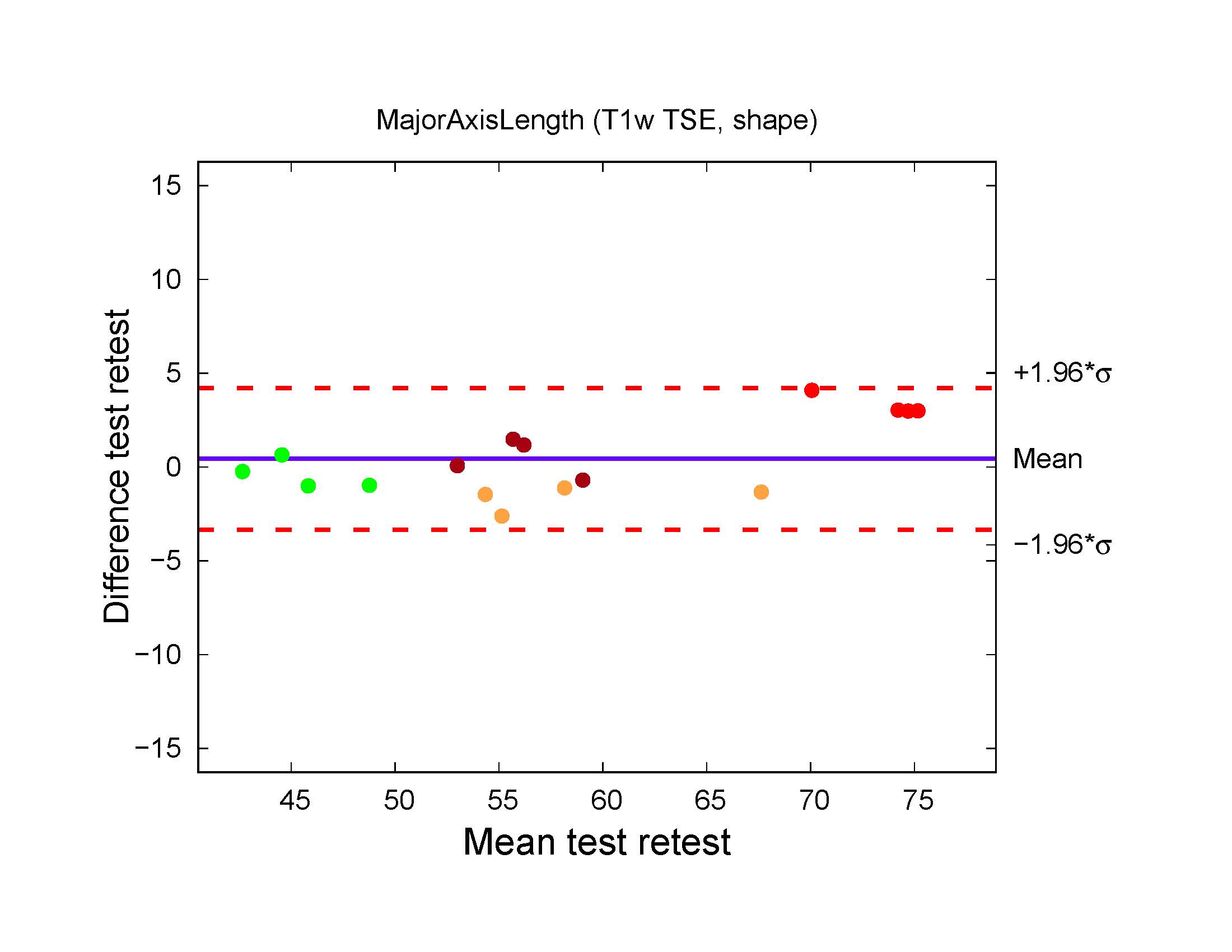

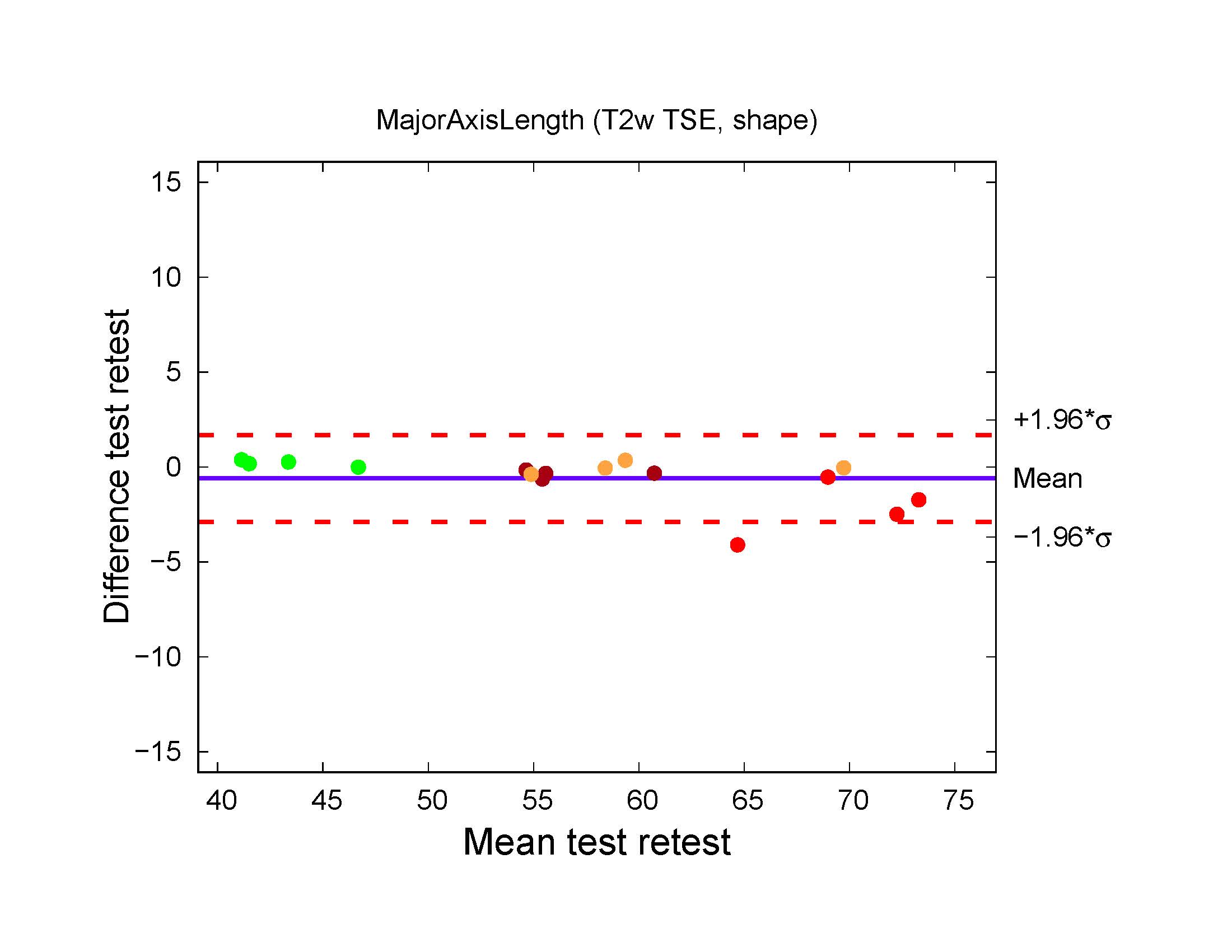

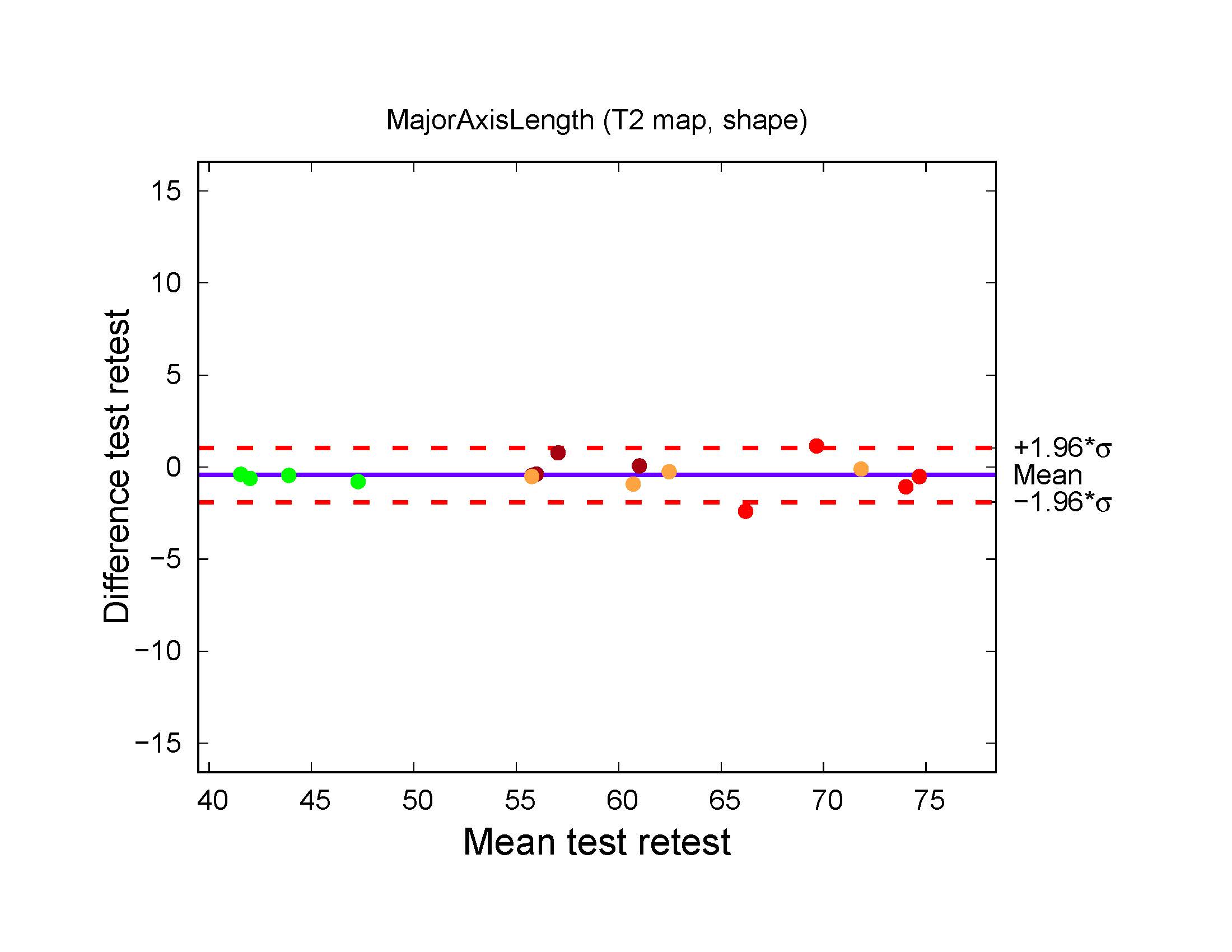

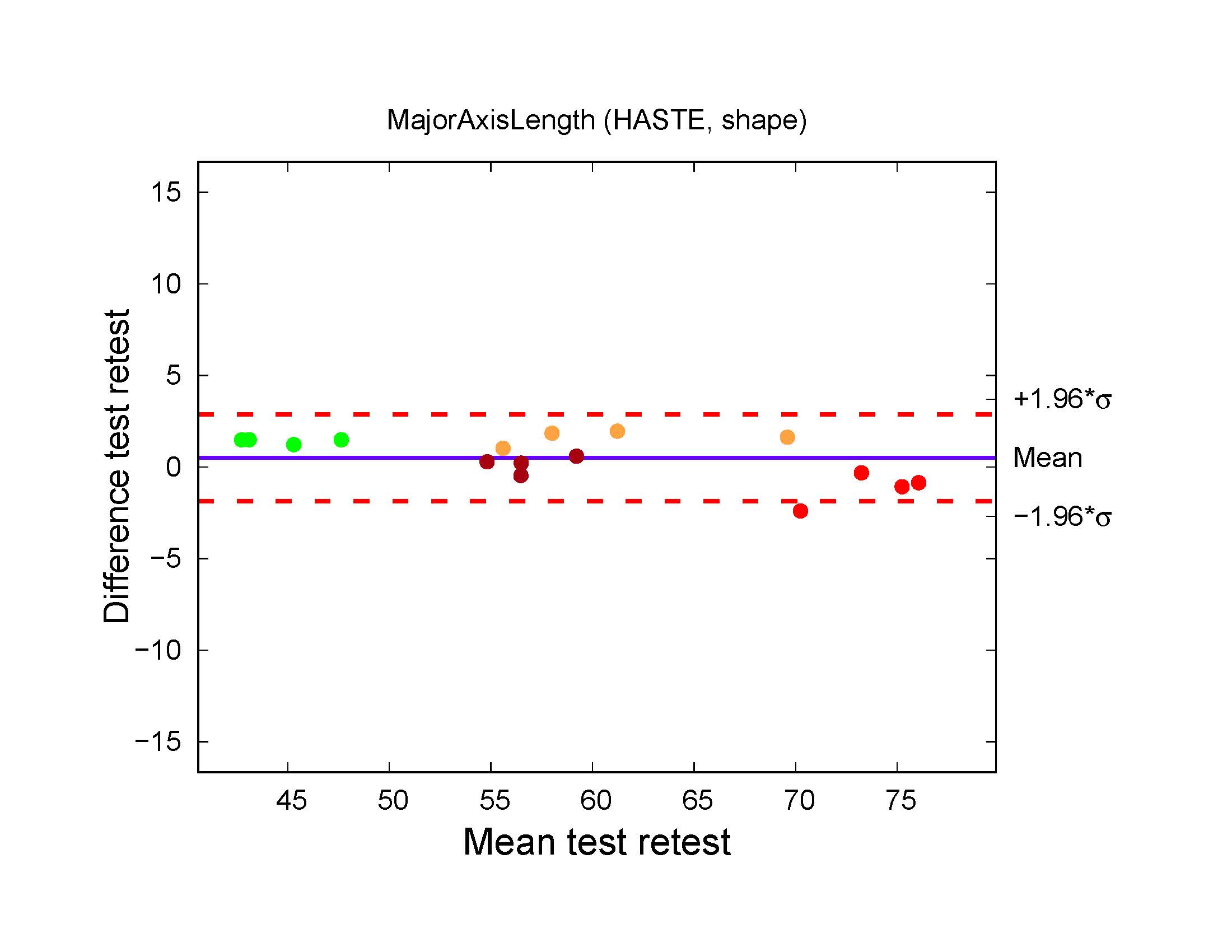

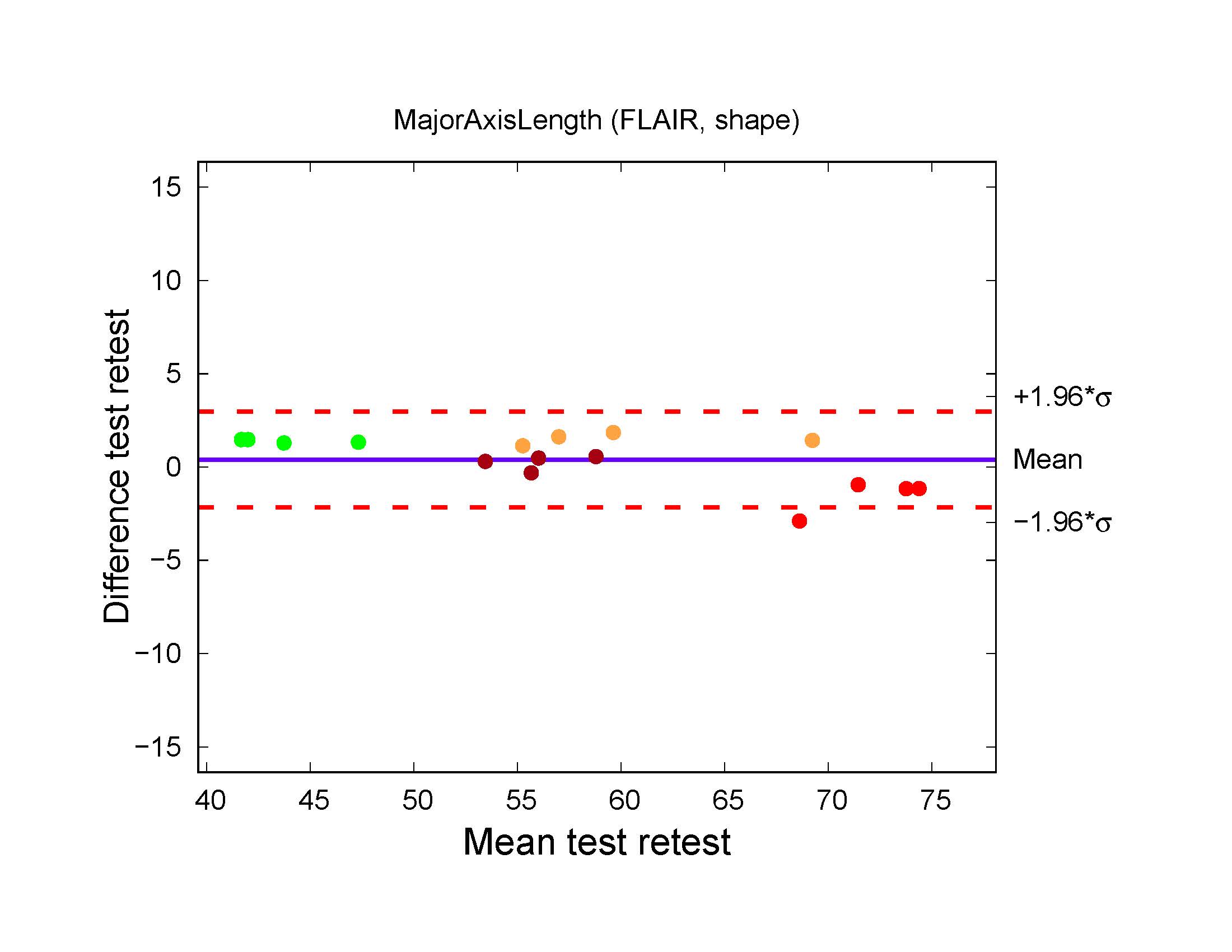


Elongation


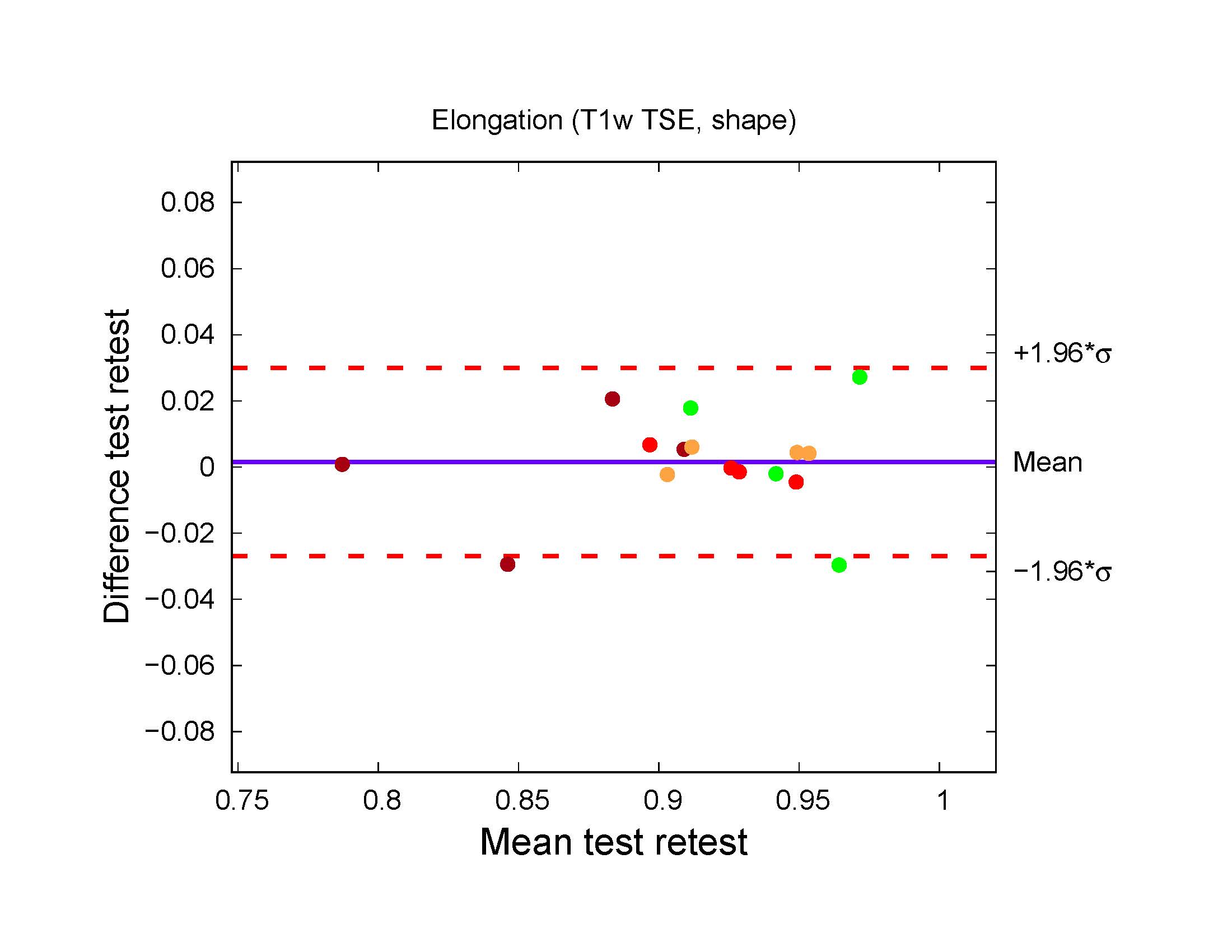

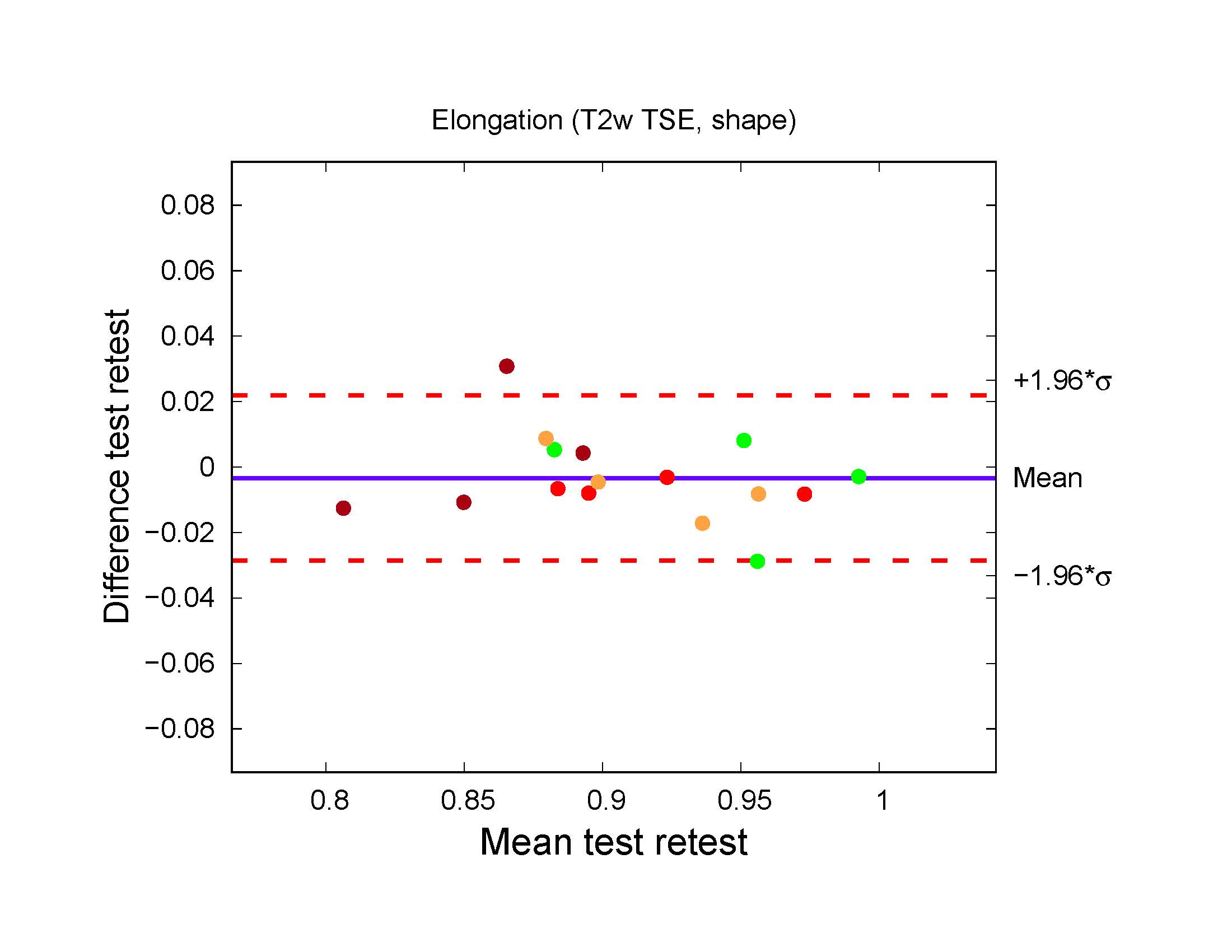

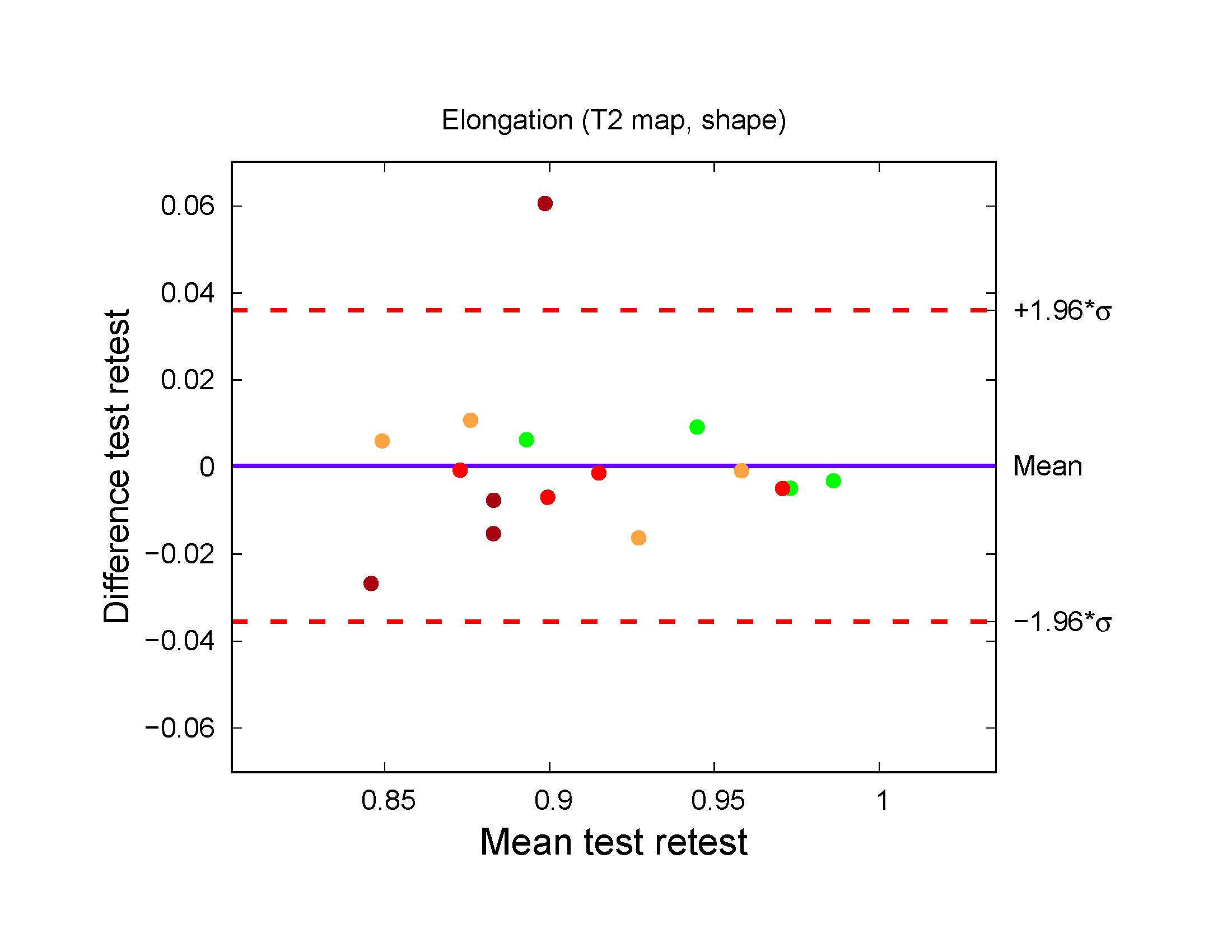

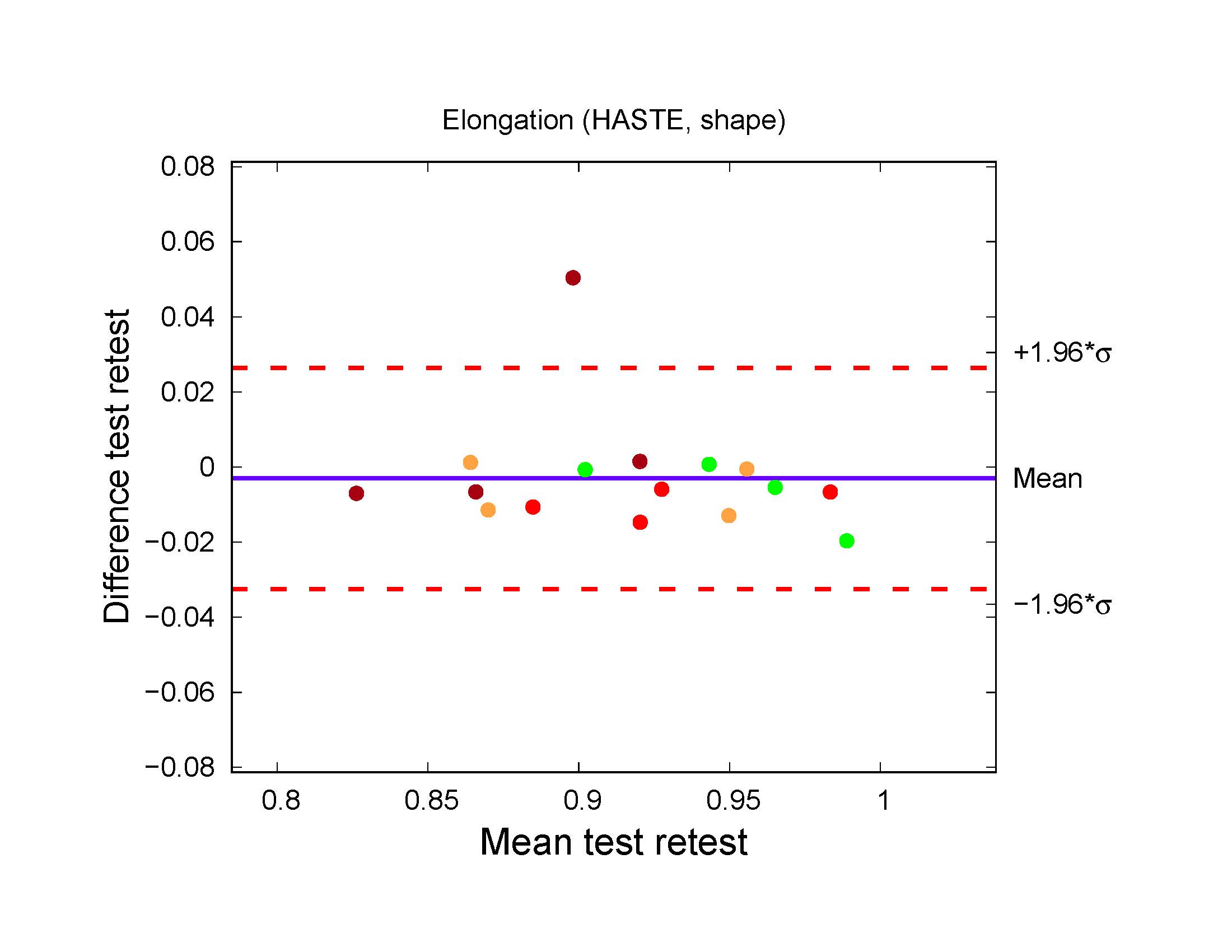

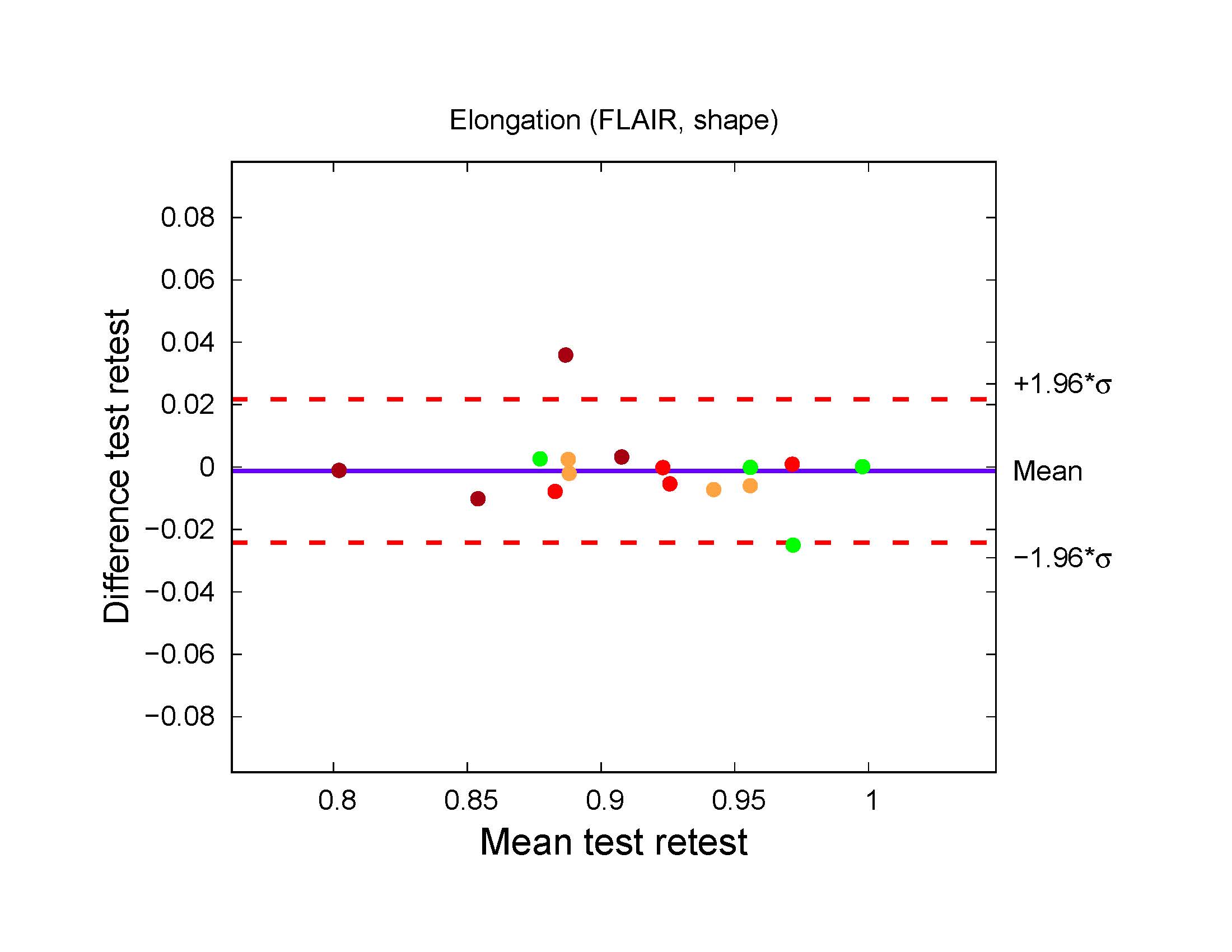


Maximum2DDiameterSlice


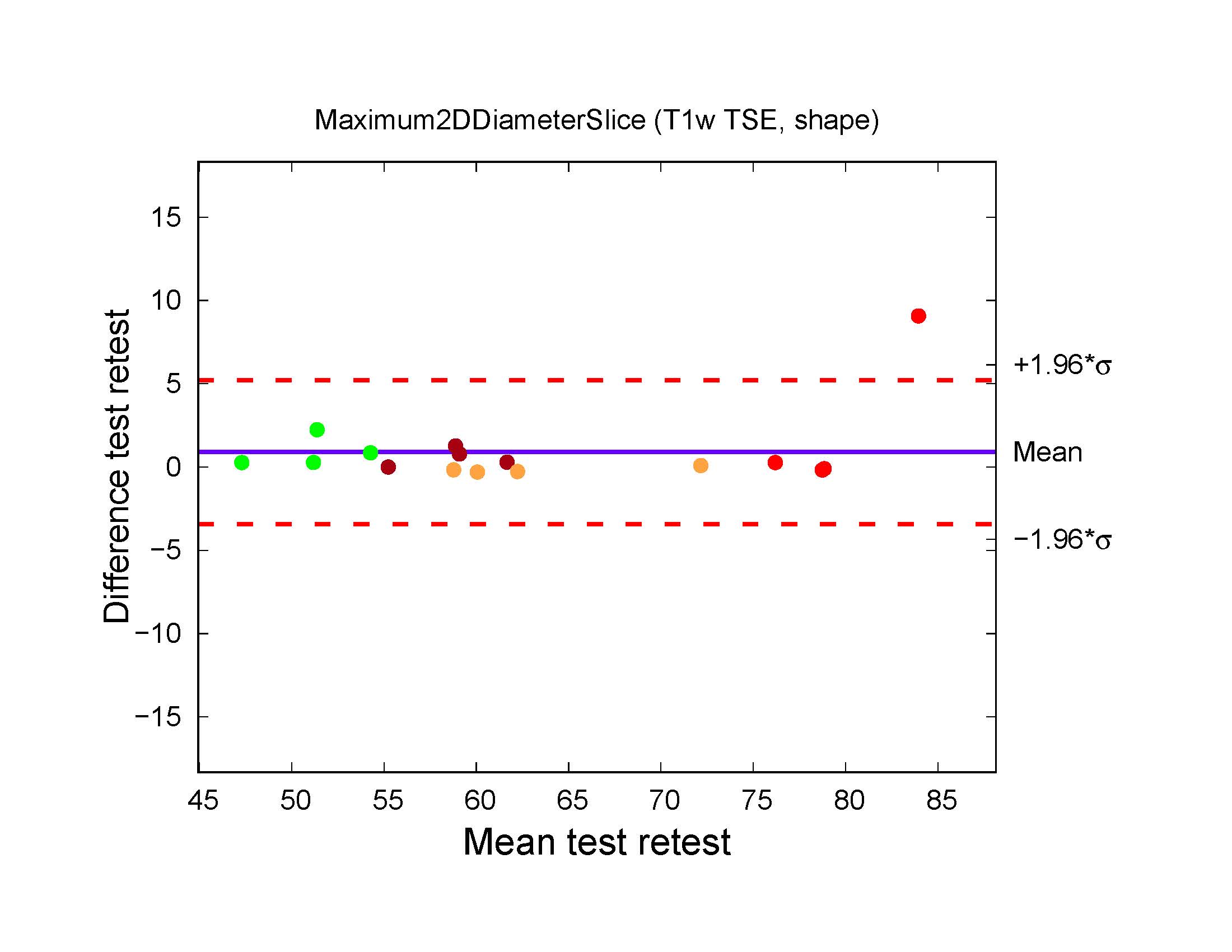

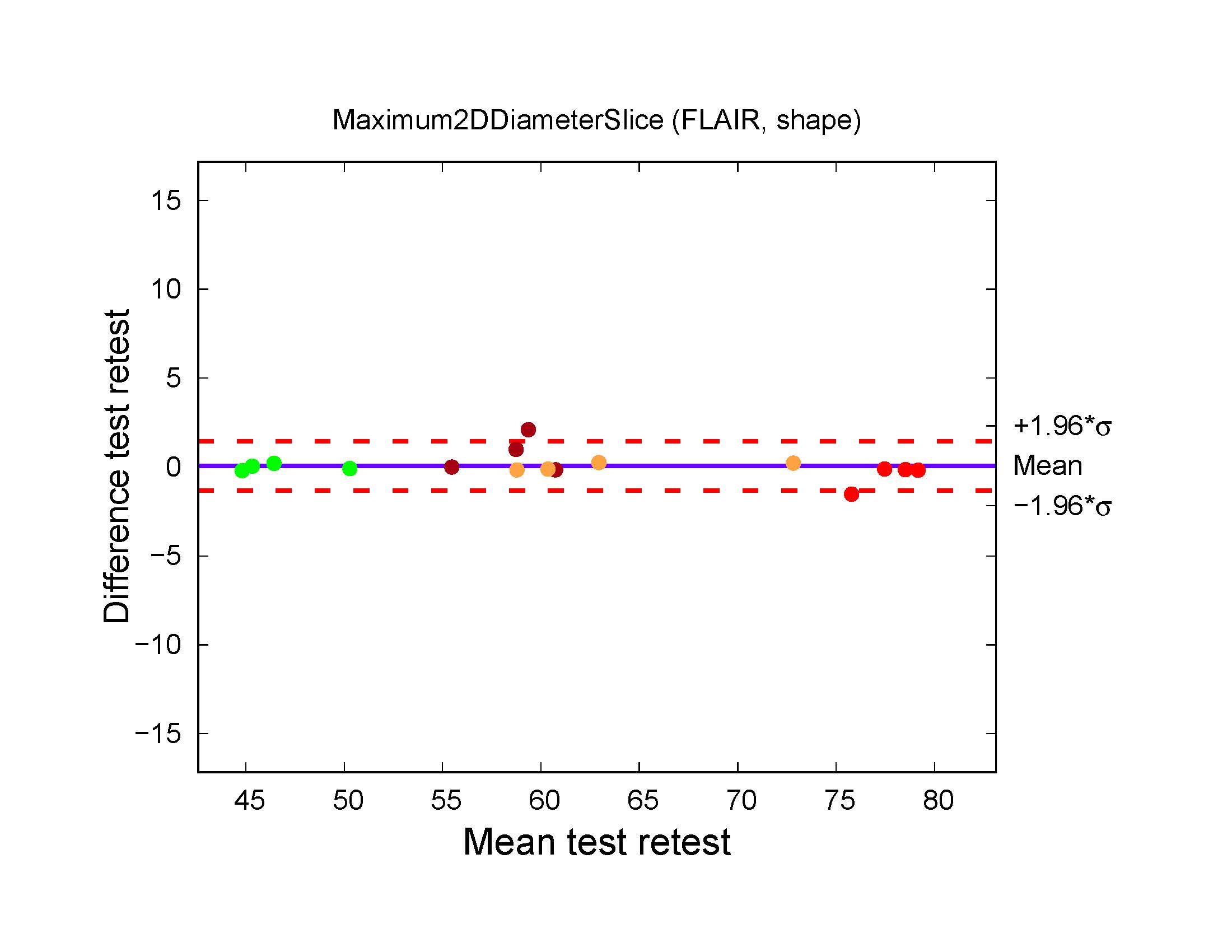

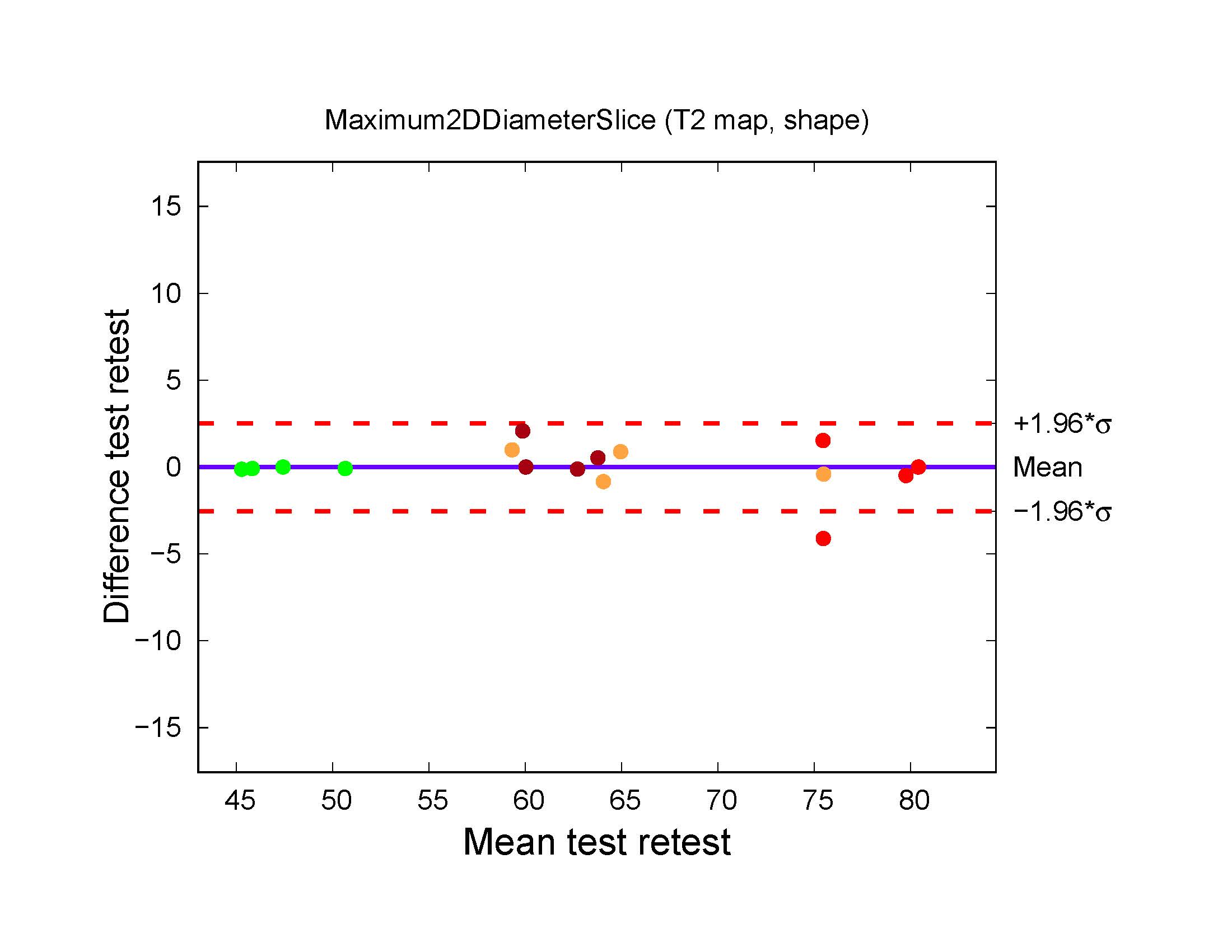

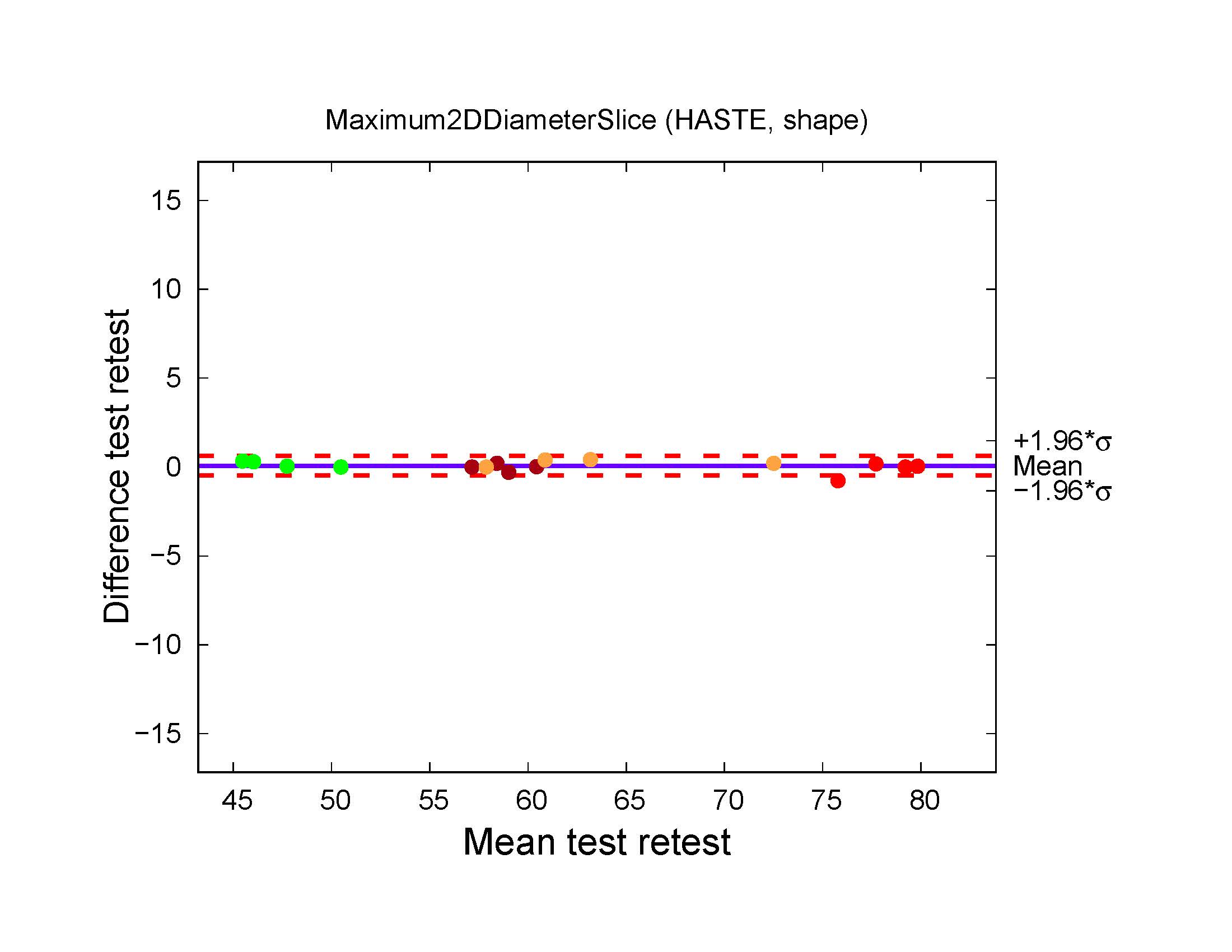

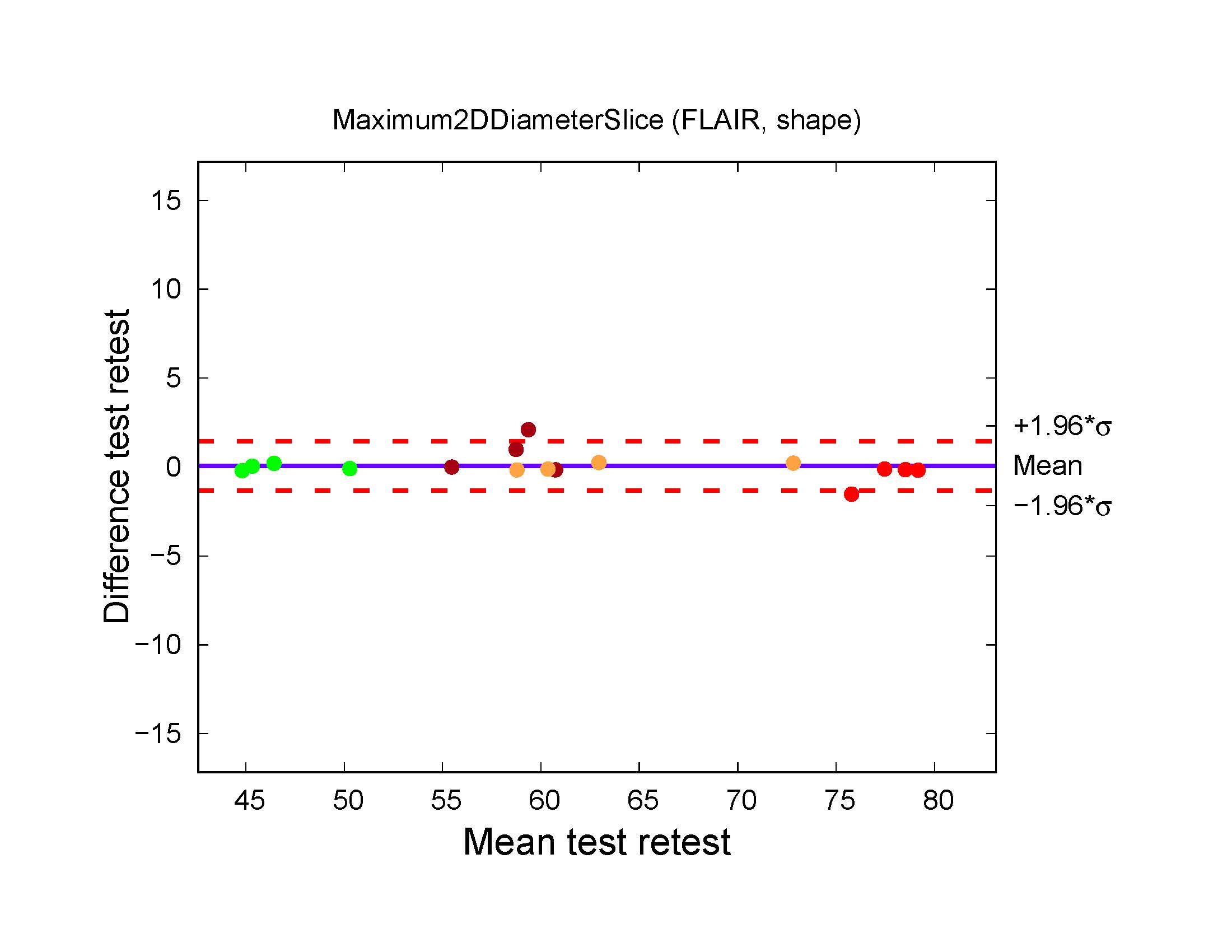


MinorAxis Length


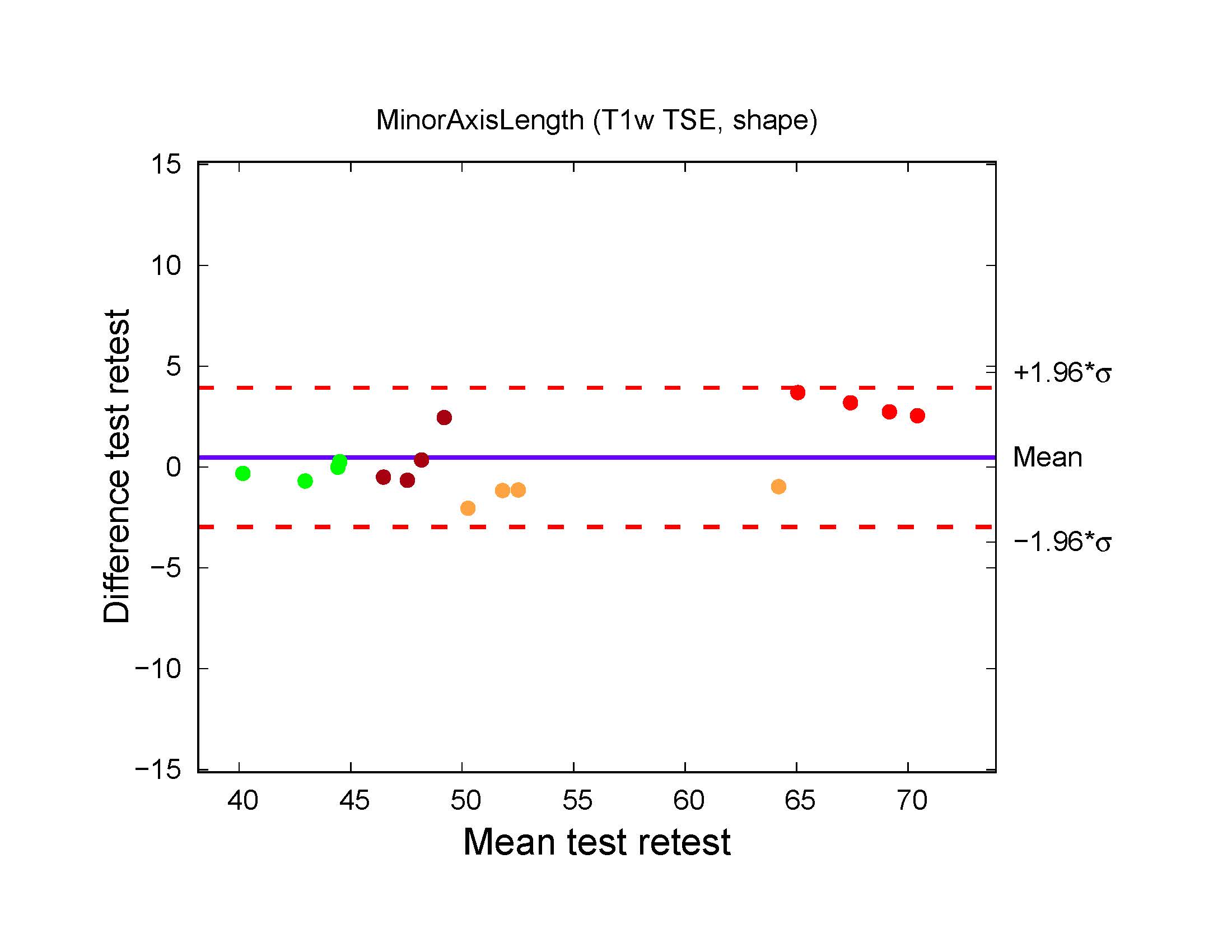

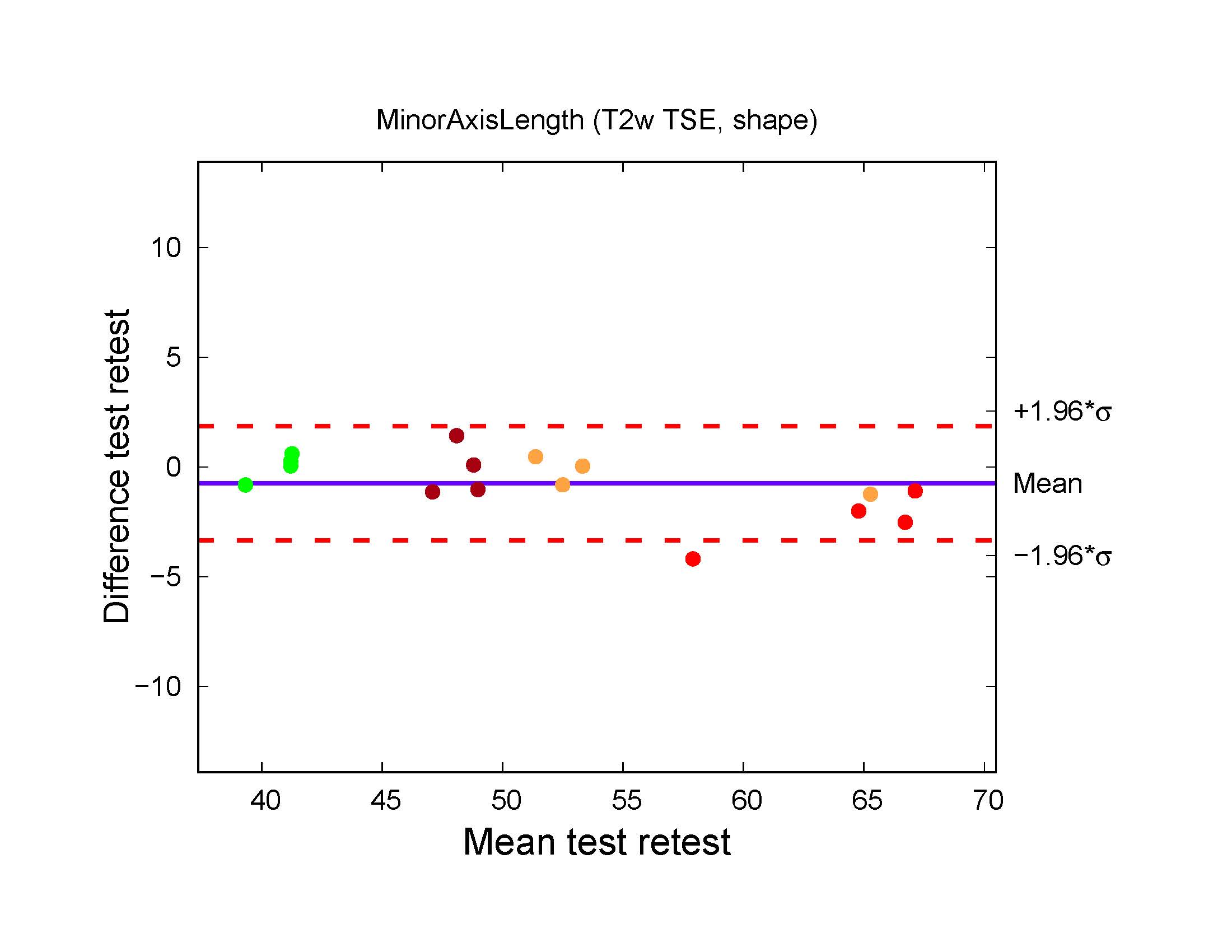

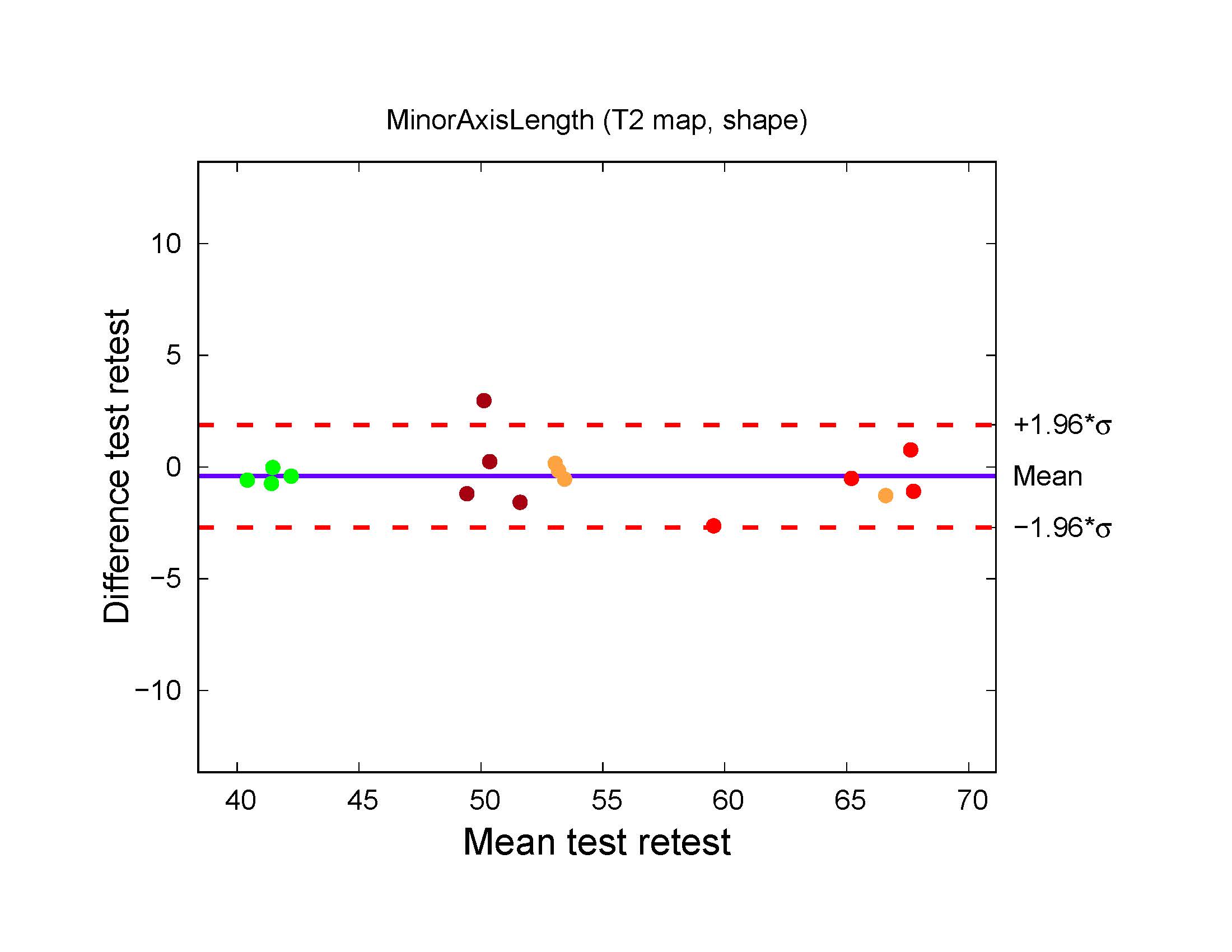

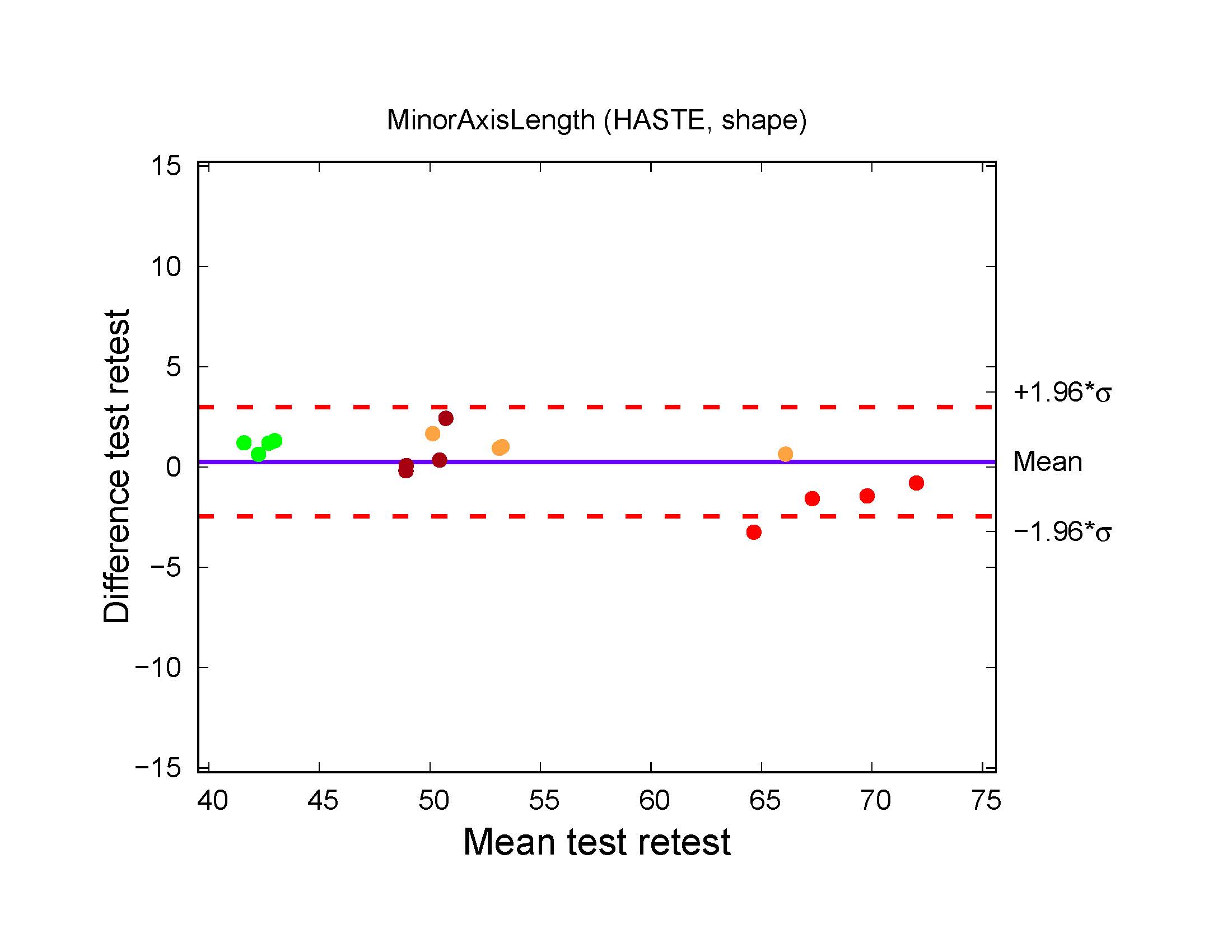

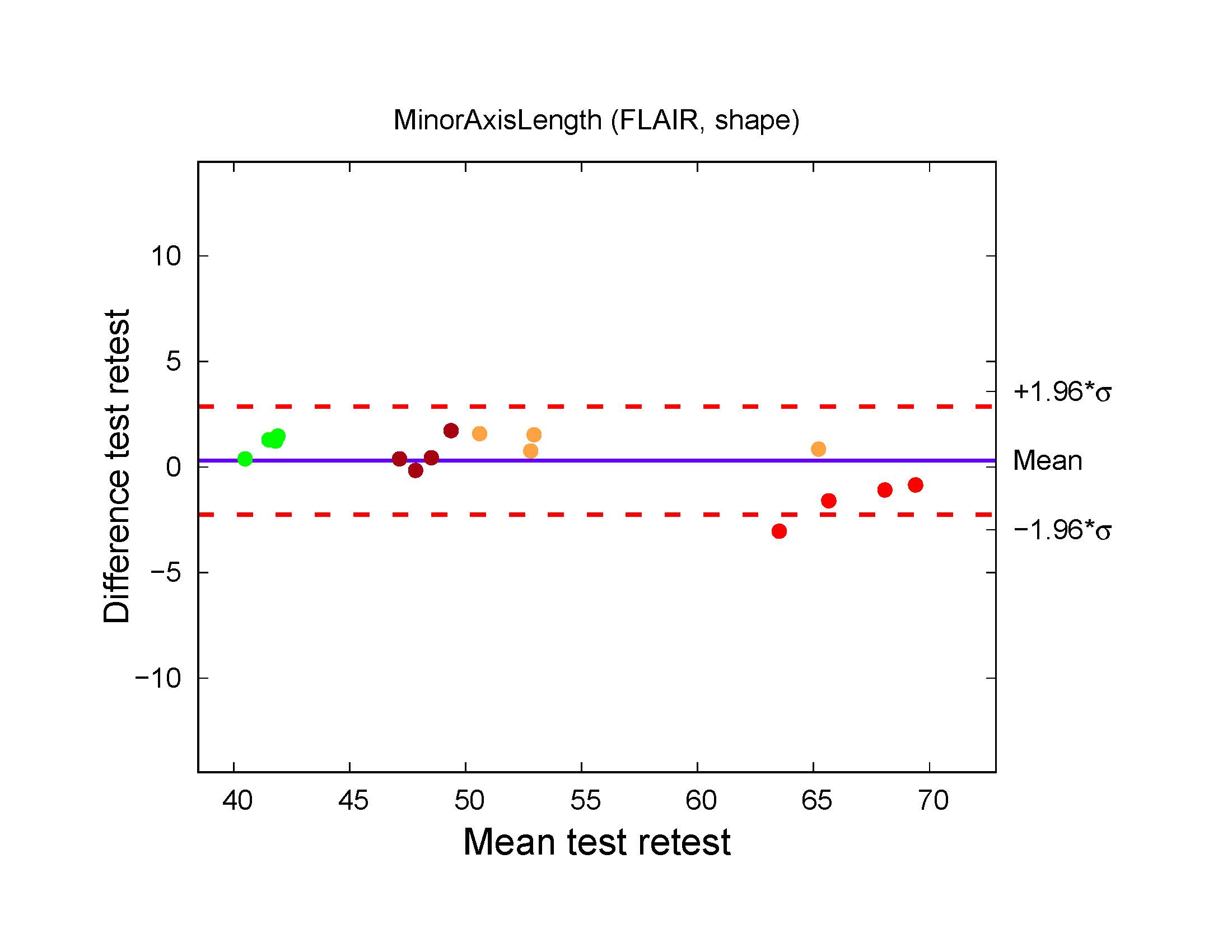


Maximum 2D Diameter Column


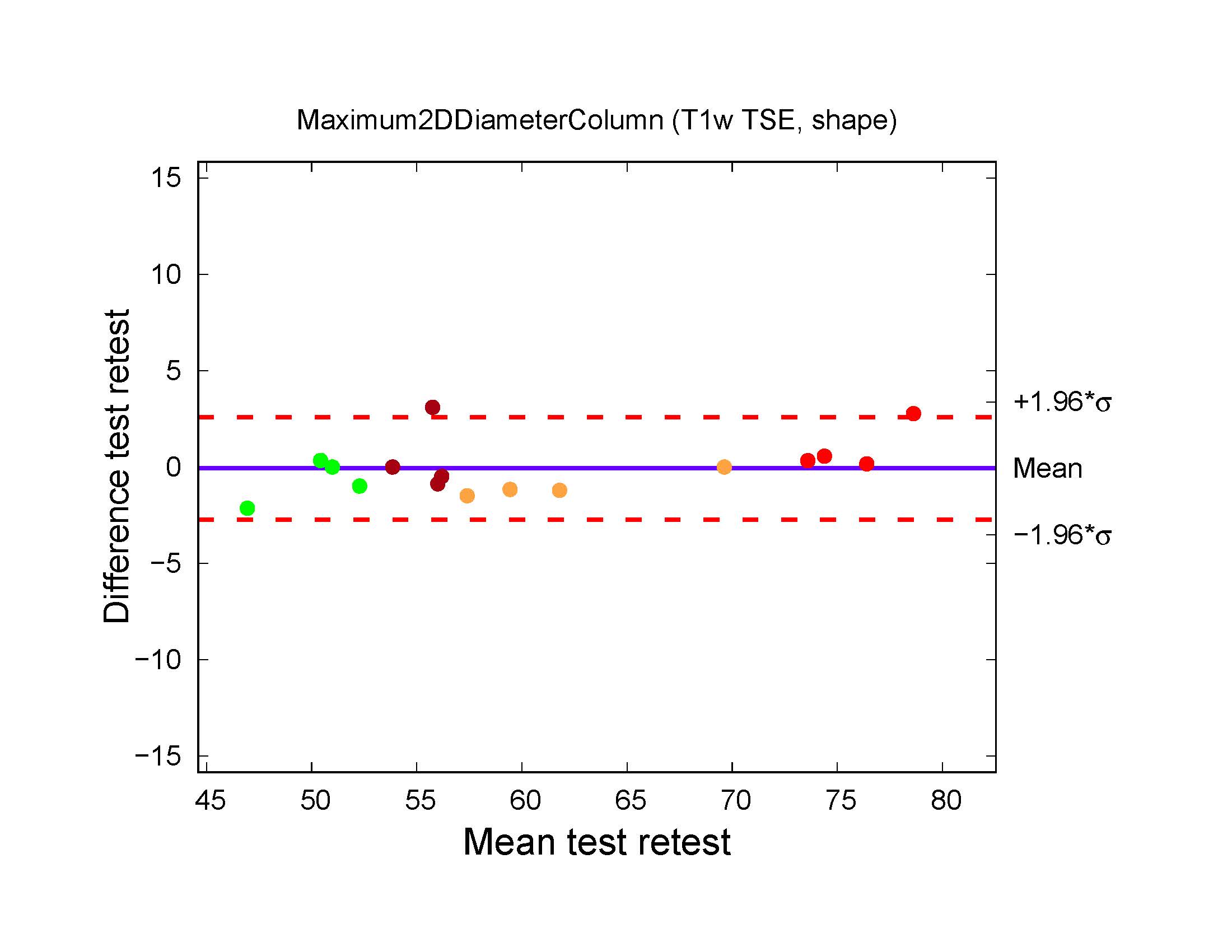

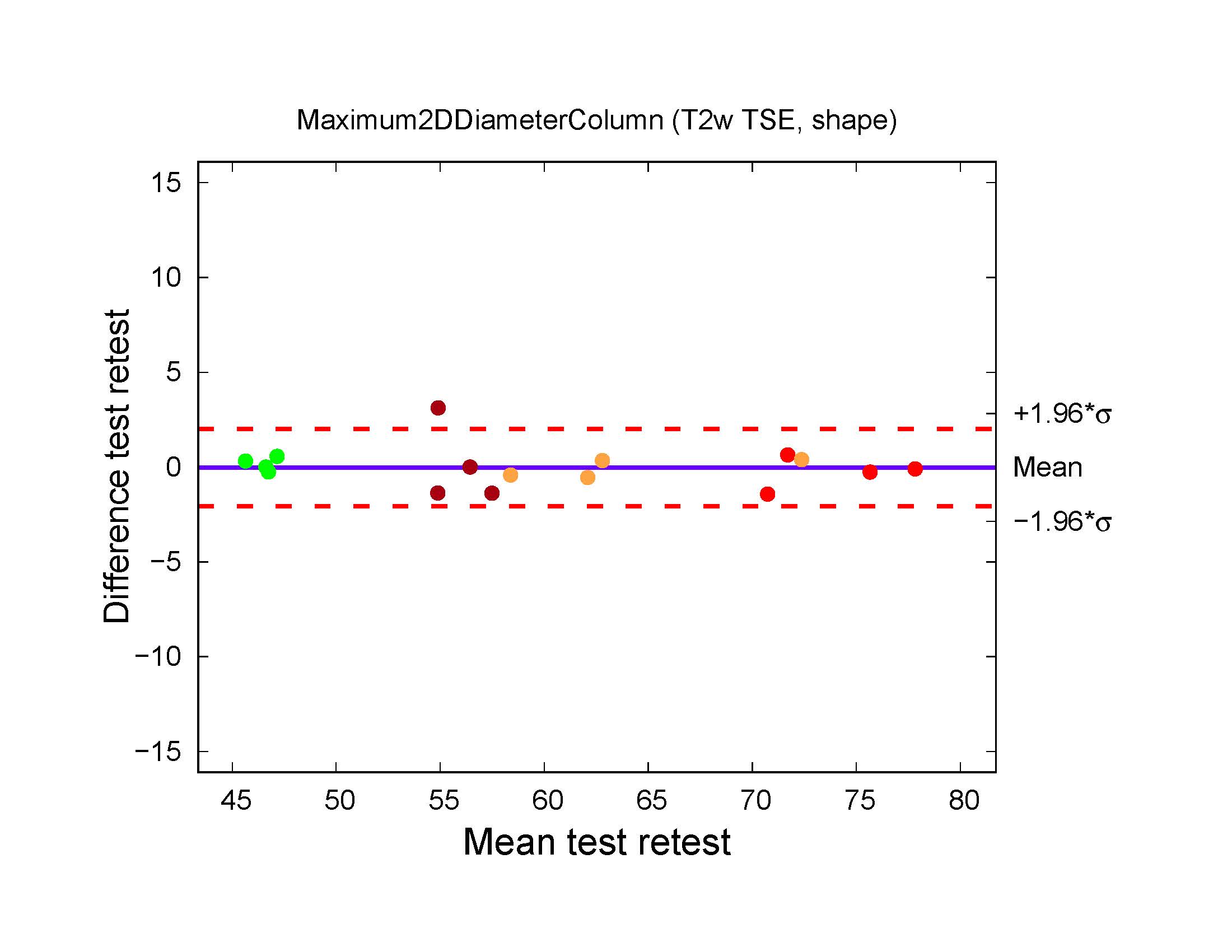

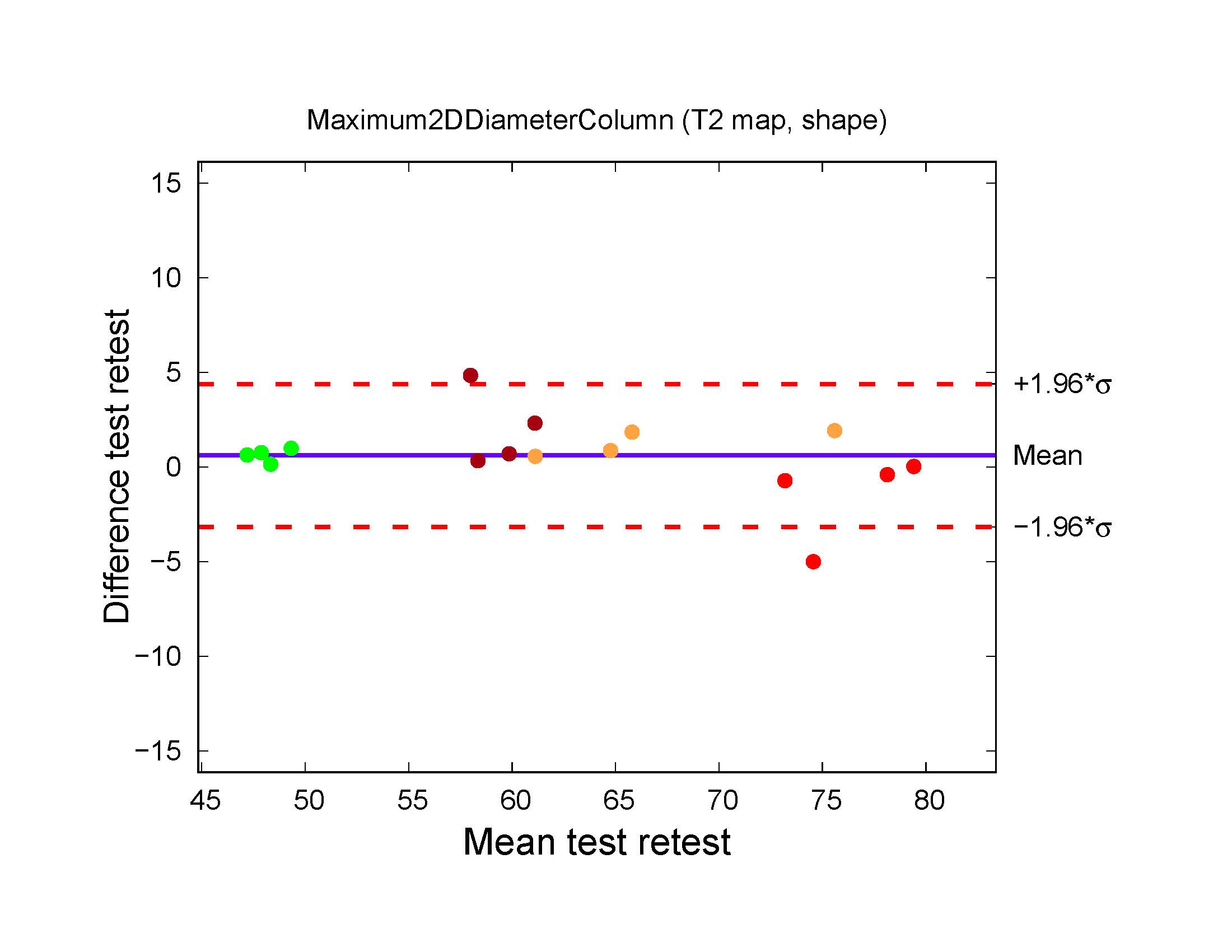

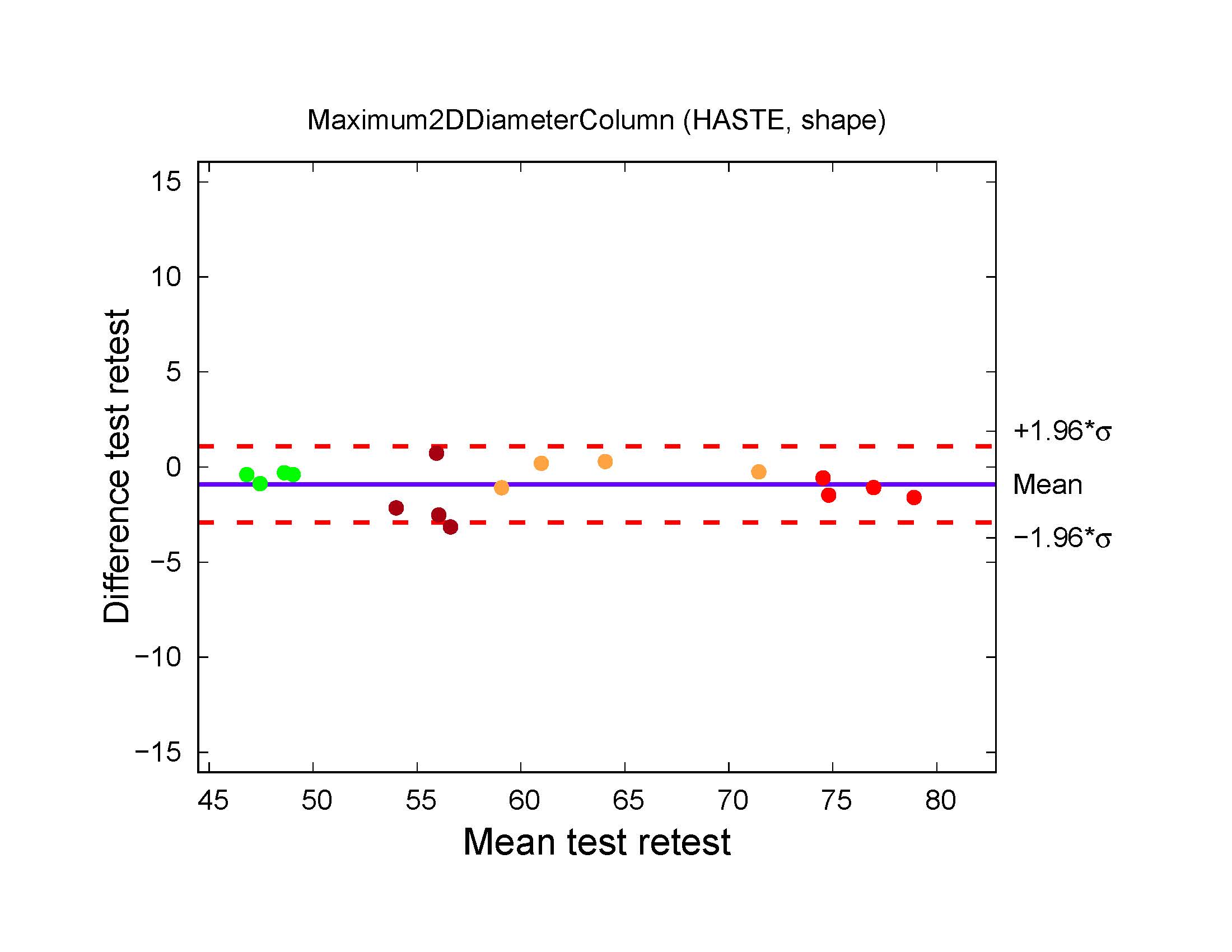

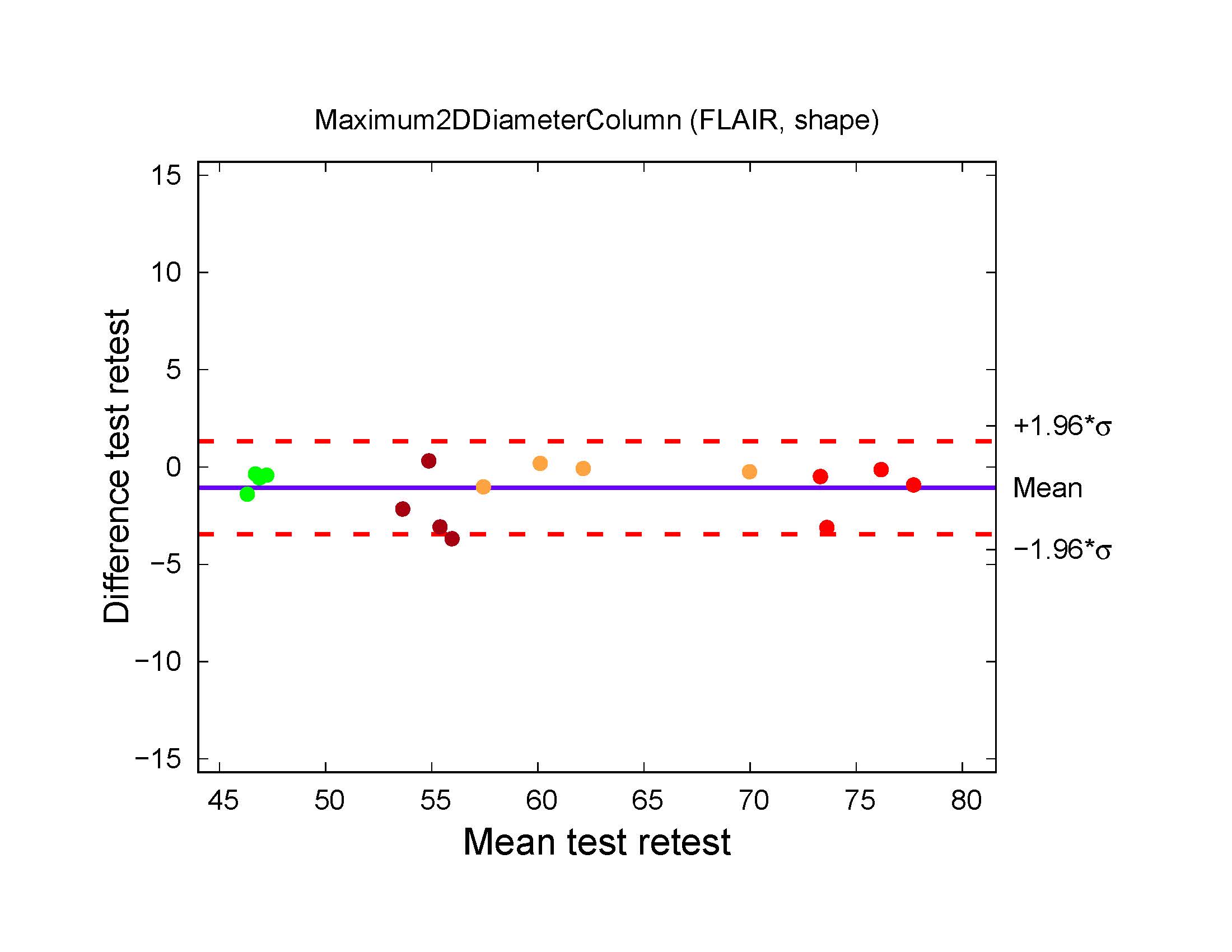


Maximum 2D diameter Row


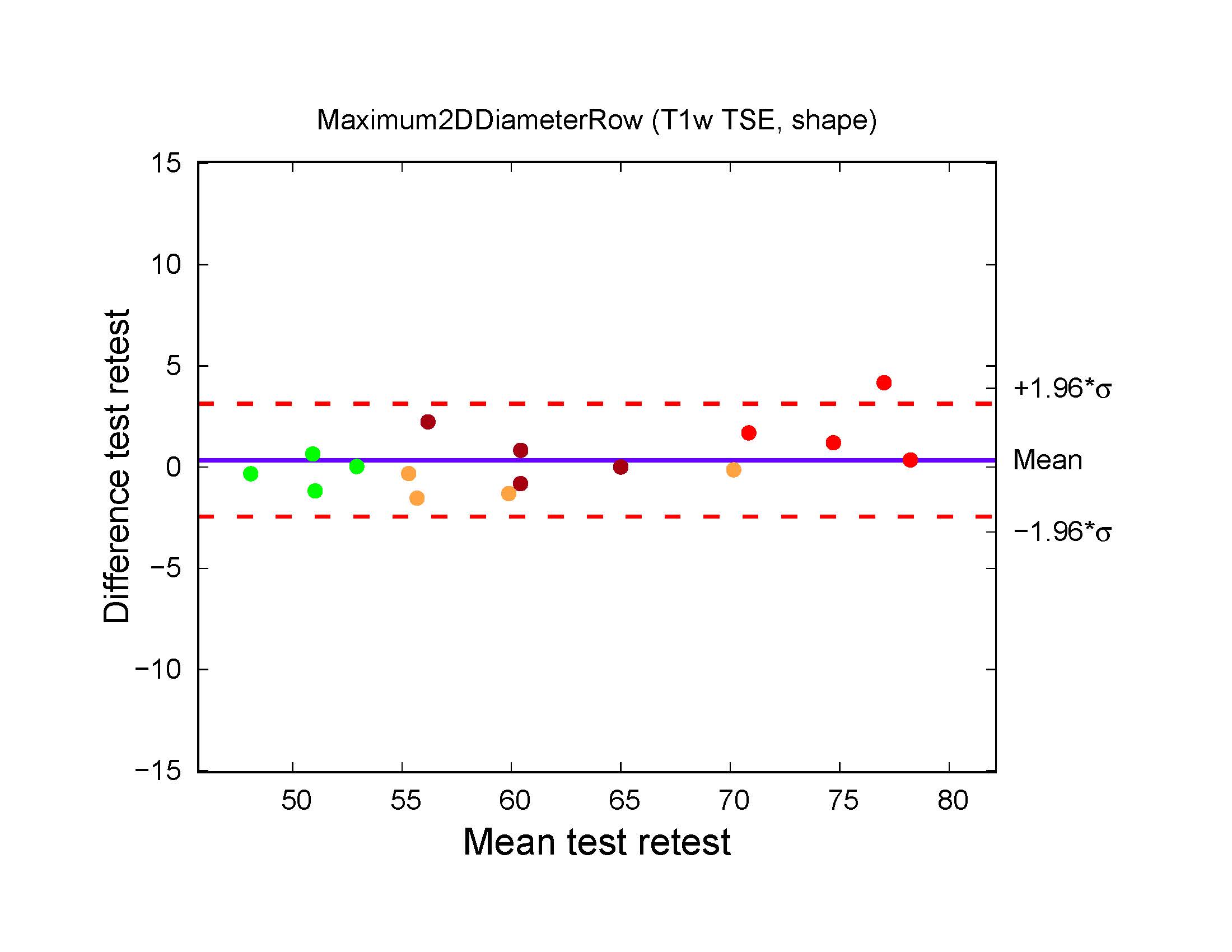

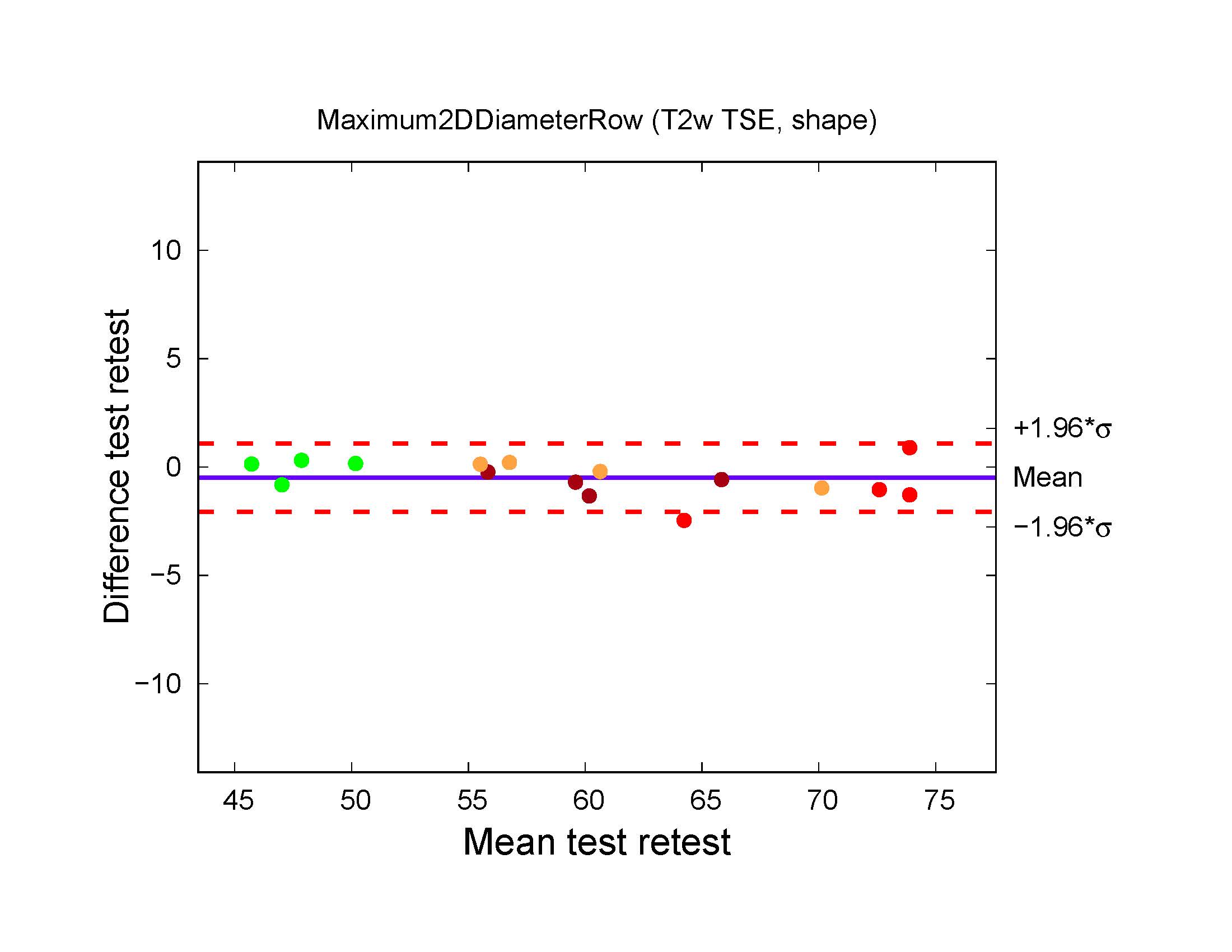

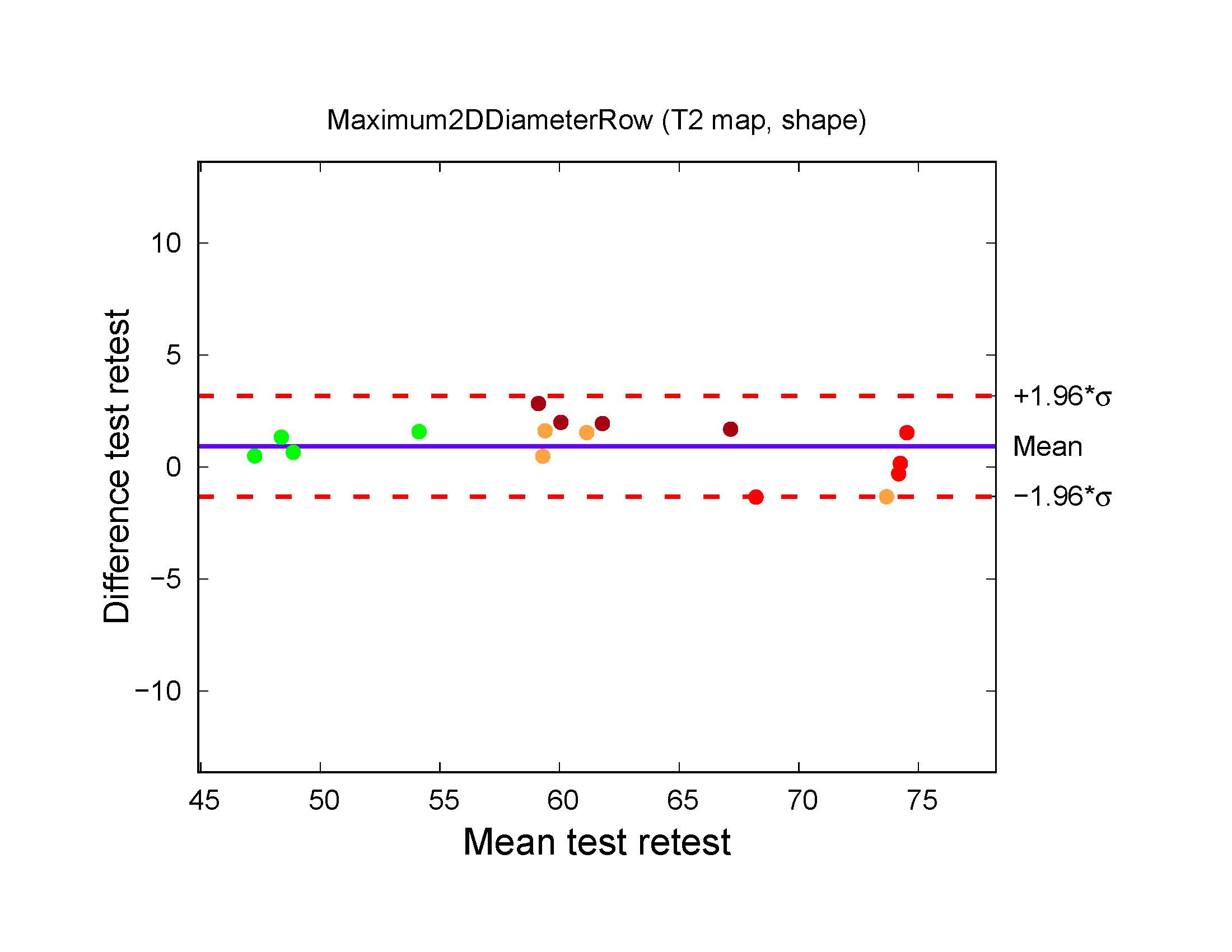

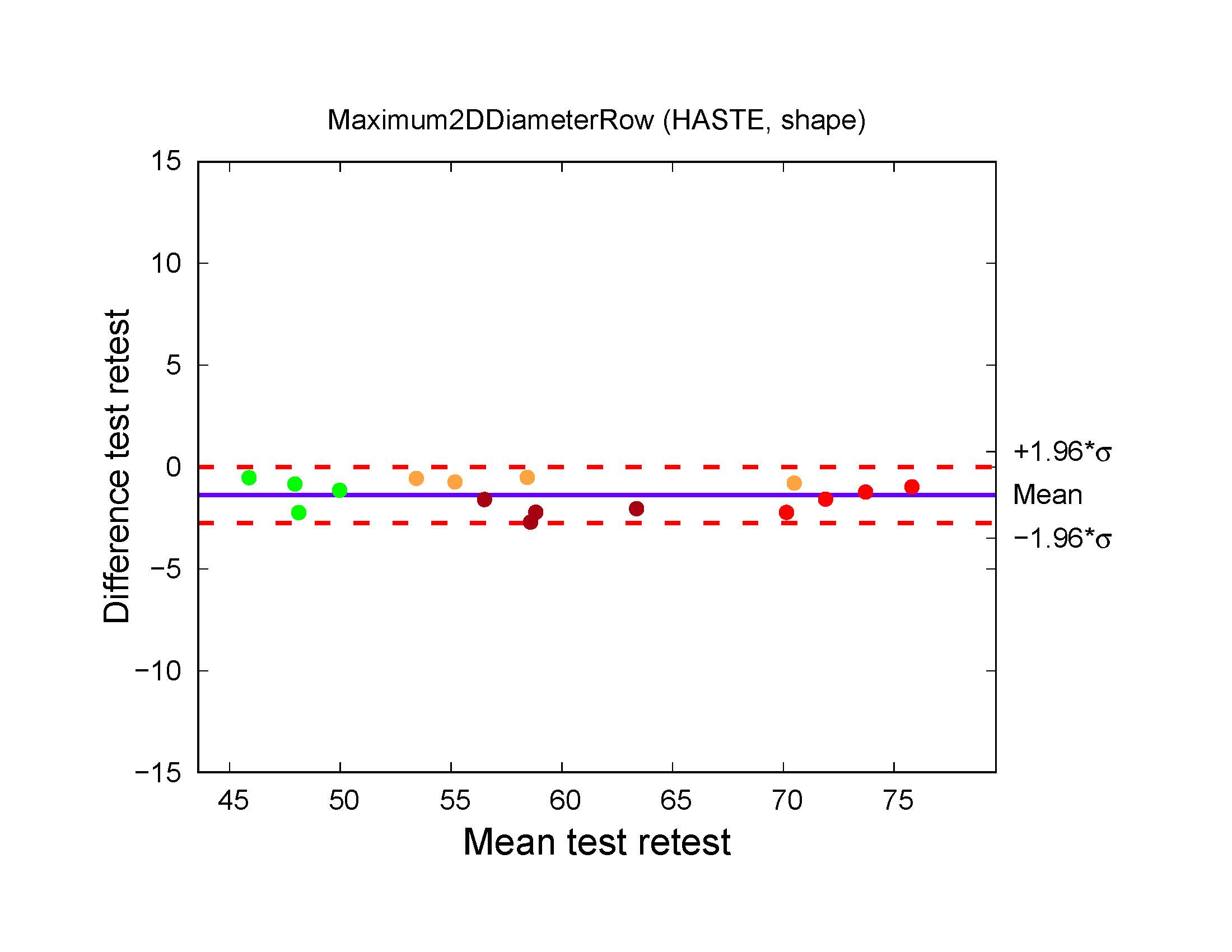

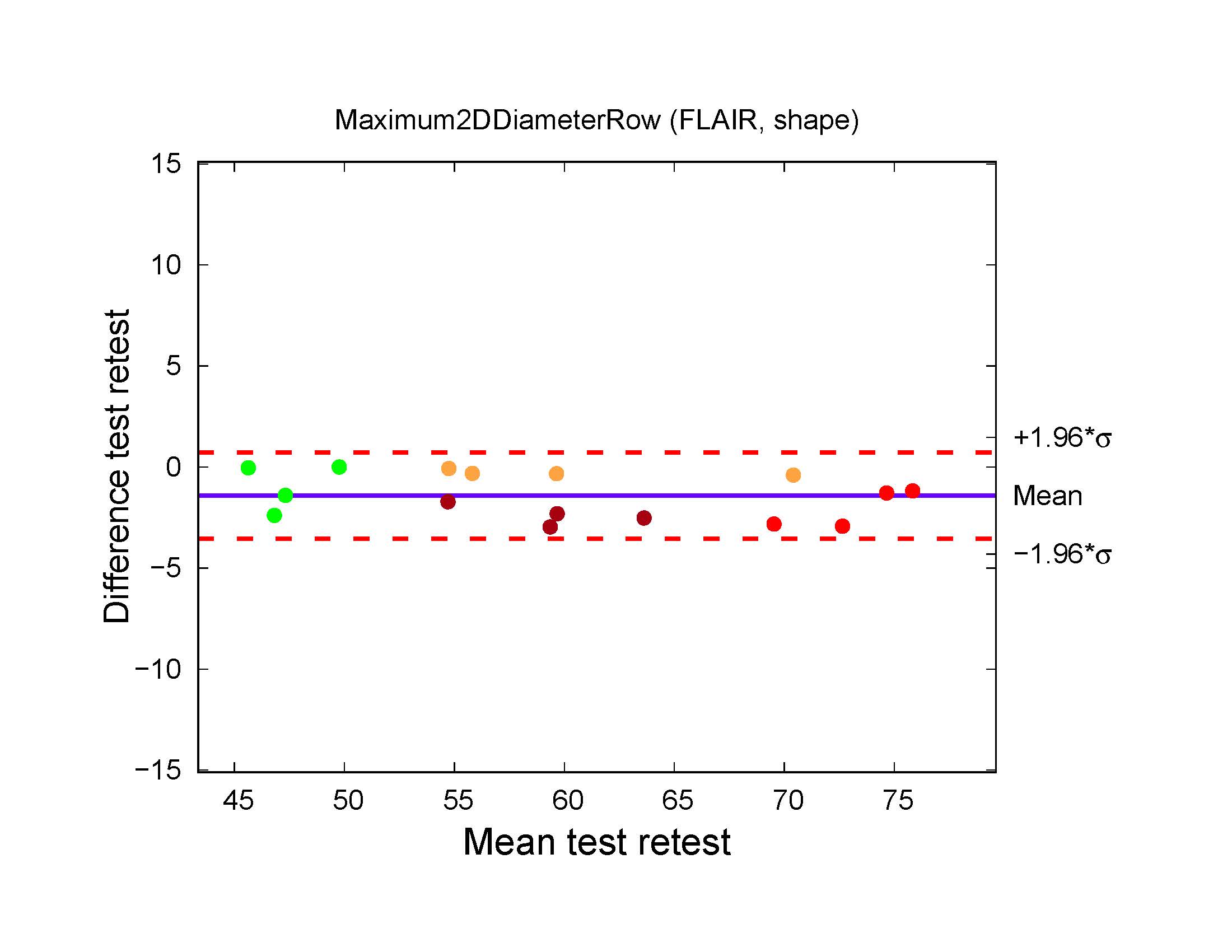


Imc1


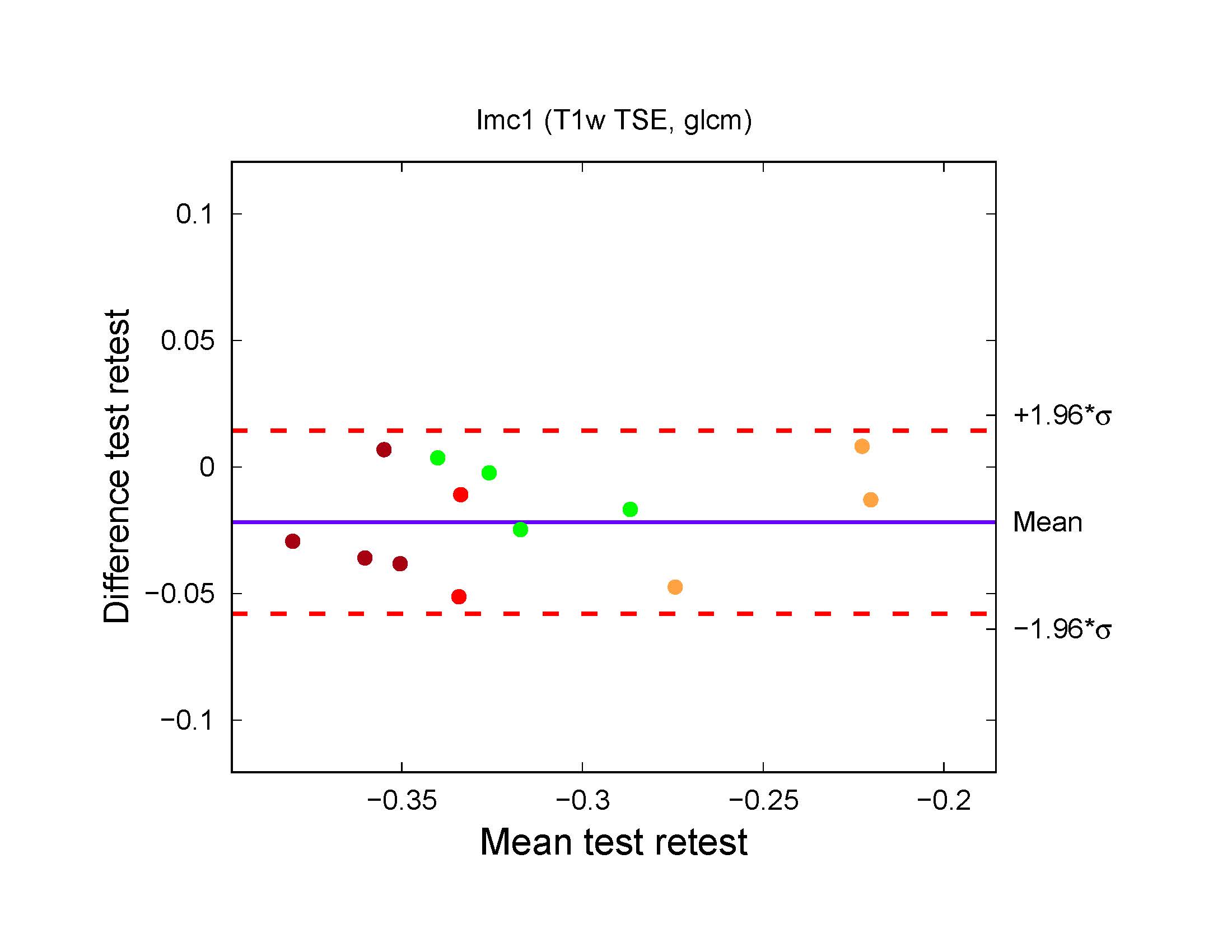

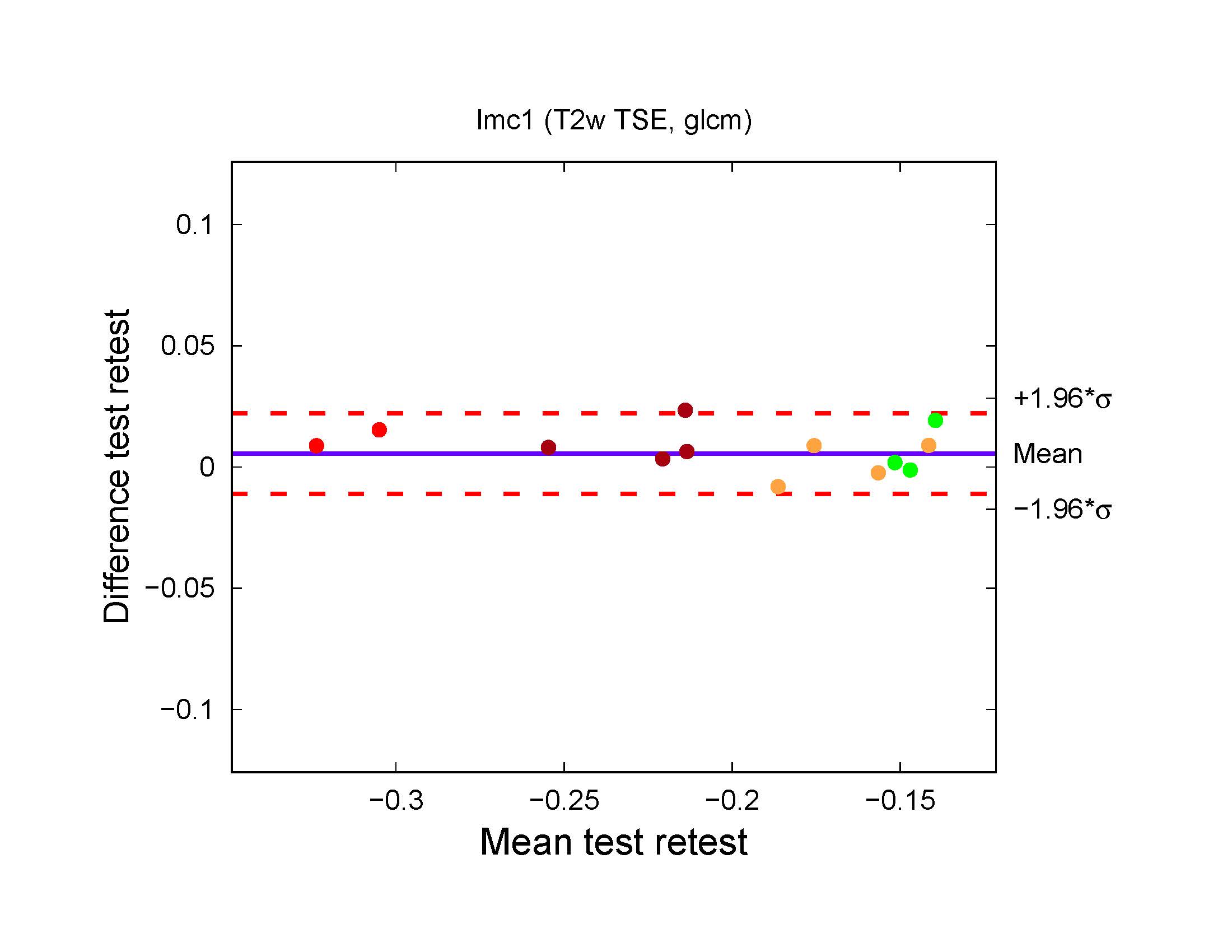

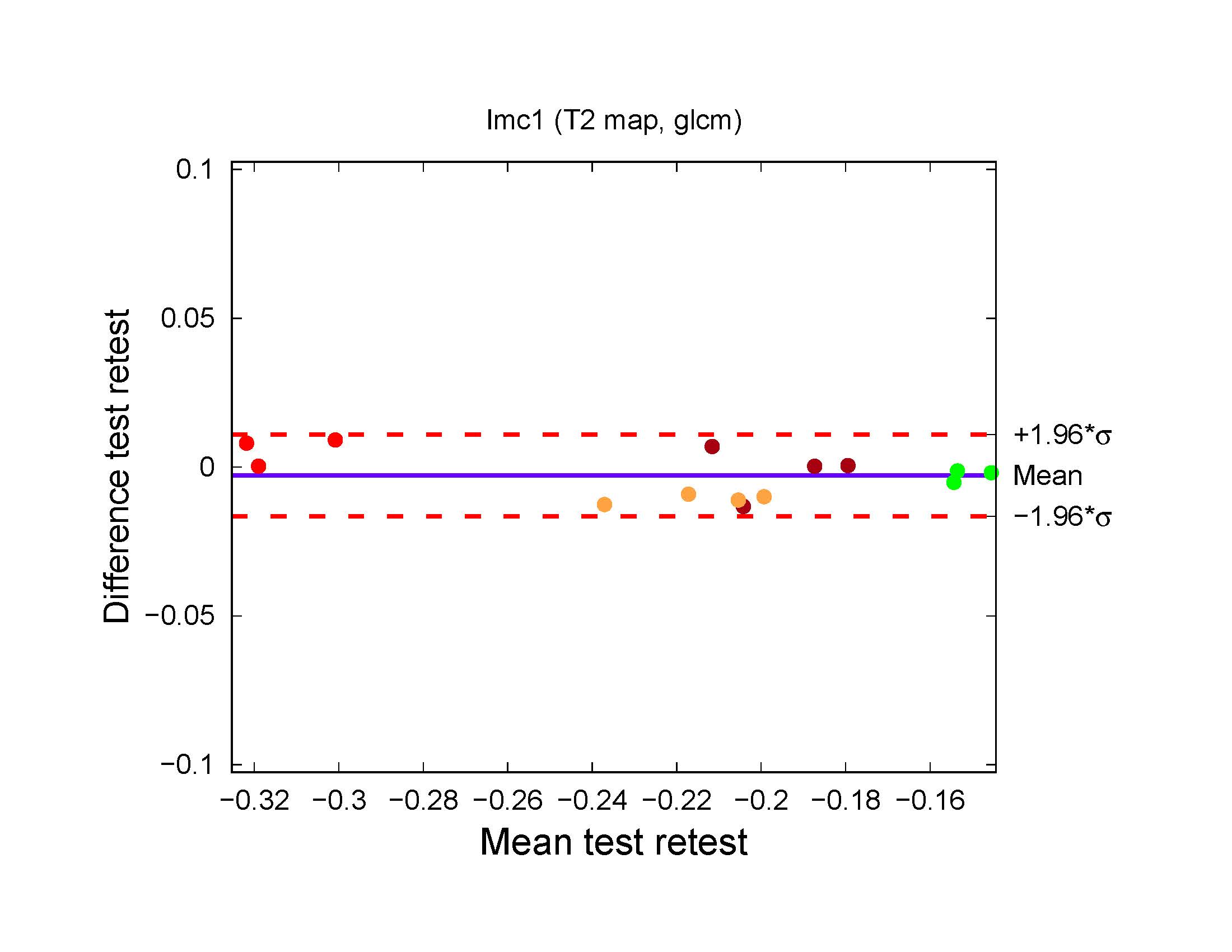

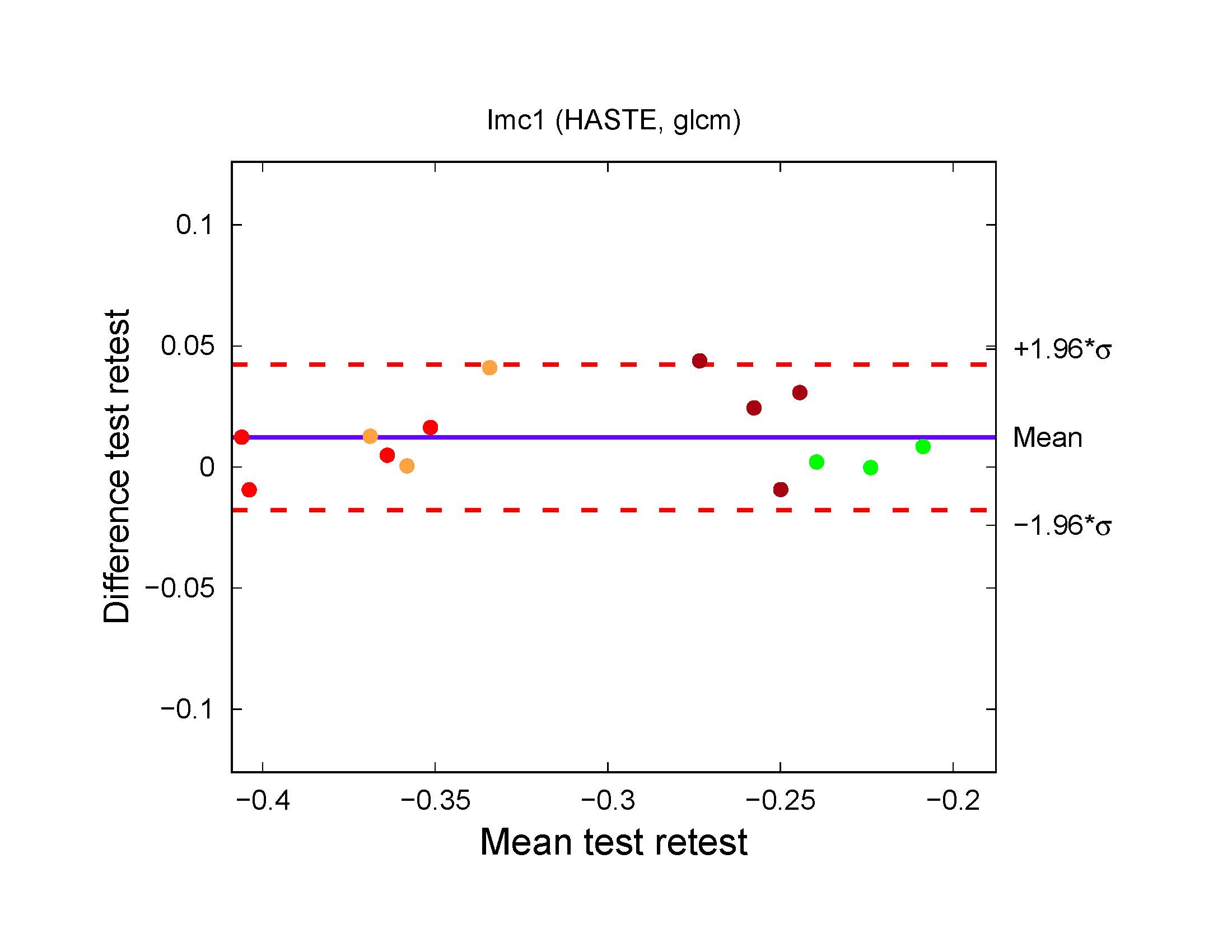

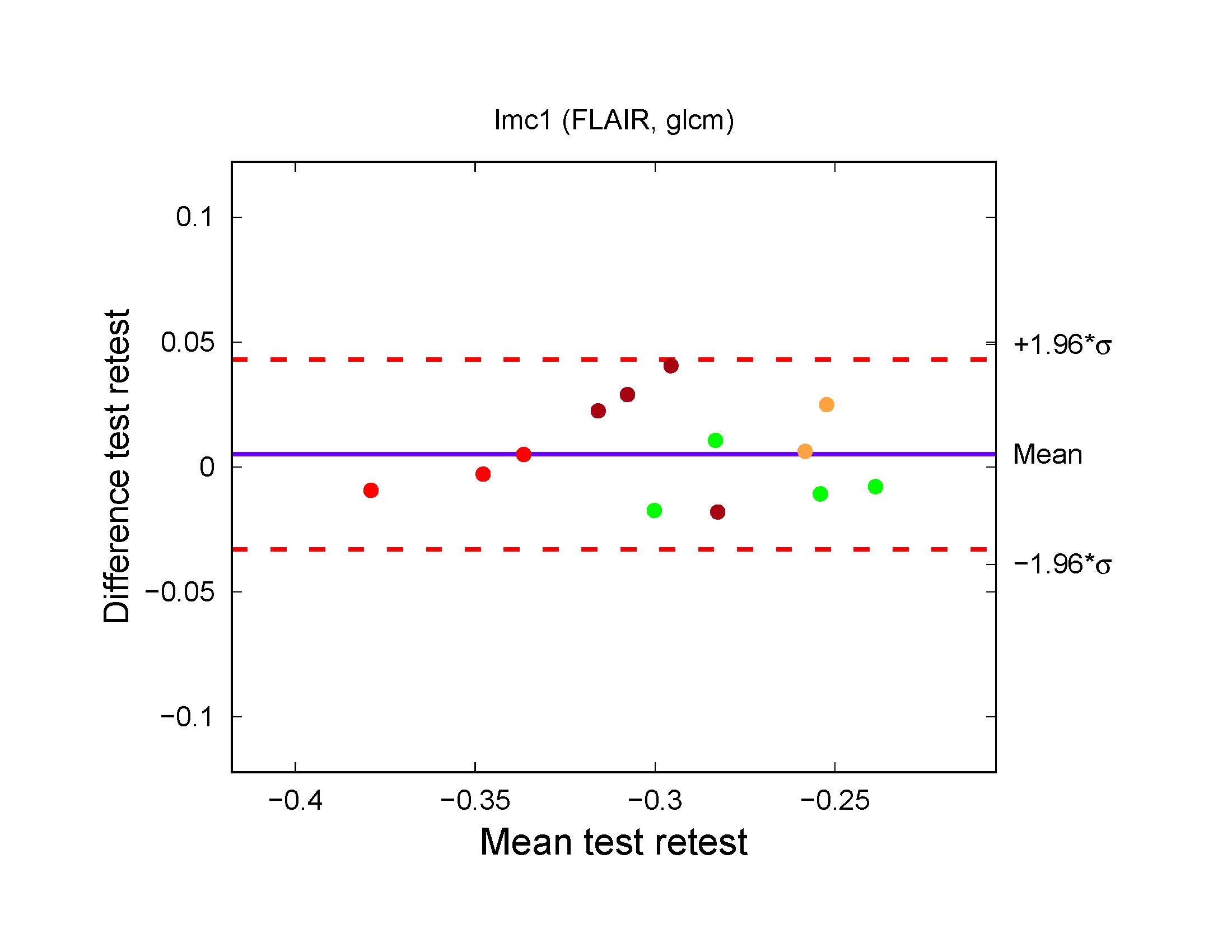

Supplement: Supplementary file 2 — Supplementary Figure 2. [file 41598_2021_93756_MOESM2_ESM.docx]
