# Supplementary material for: Impact of rescanning and repositioning on radiomic features employing a multi-object phantom in magnetic resonance imaging
Source: Sci Rep. 2021 Jul 9;11:14248. doi: 10.1038/s41598-021-93756-x (PMC8271025; doi:10.1038/s41598-021-93756-x)

**Suppl. Figure 3:** Bland Altmann plots of features with top discriminative power (Mann–Whitney U test). The features are ranked according to the number of perfect Gini score, 100%, in 120 tests to distinguish pairs of fruits. Each colored point represents a fruit. The color code is green for lemon, red for apple, brown for kiwi, and sandybrown for onion. Plots are produced with the program gnuplot. Ideally, the points should be well separated from each other. The differences, plotted on the y-axes, should show preferable small random fluctuations around zero.

Rank 1:


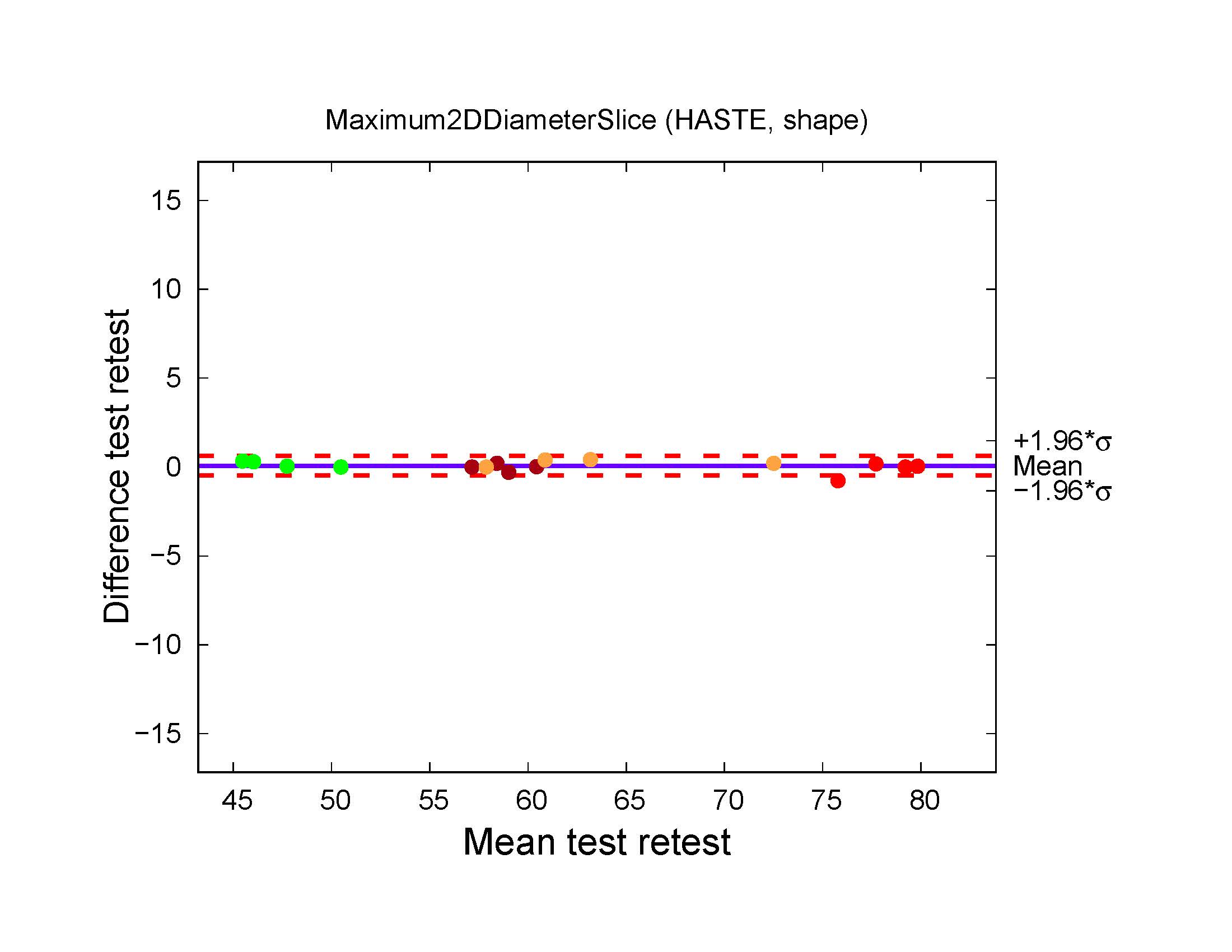


Rank 2:


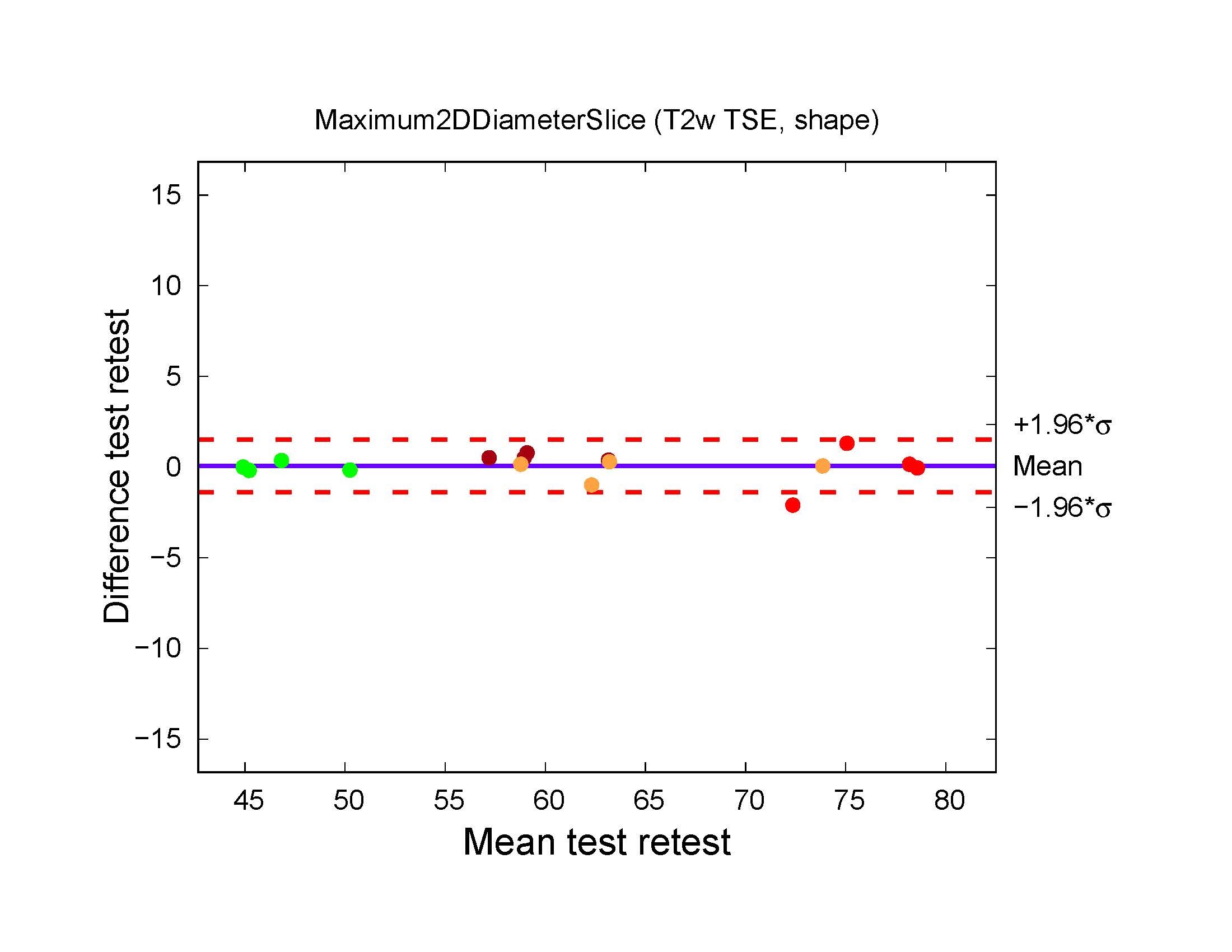

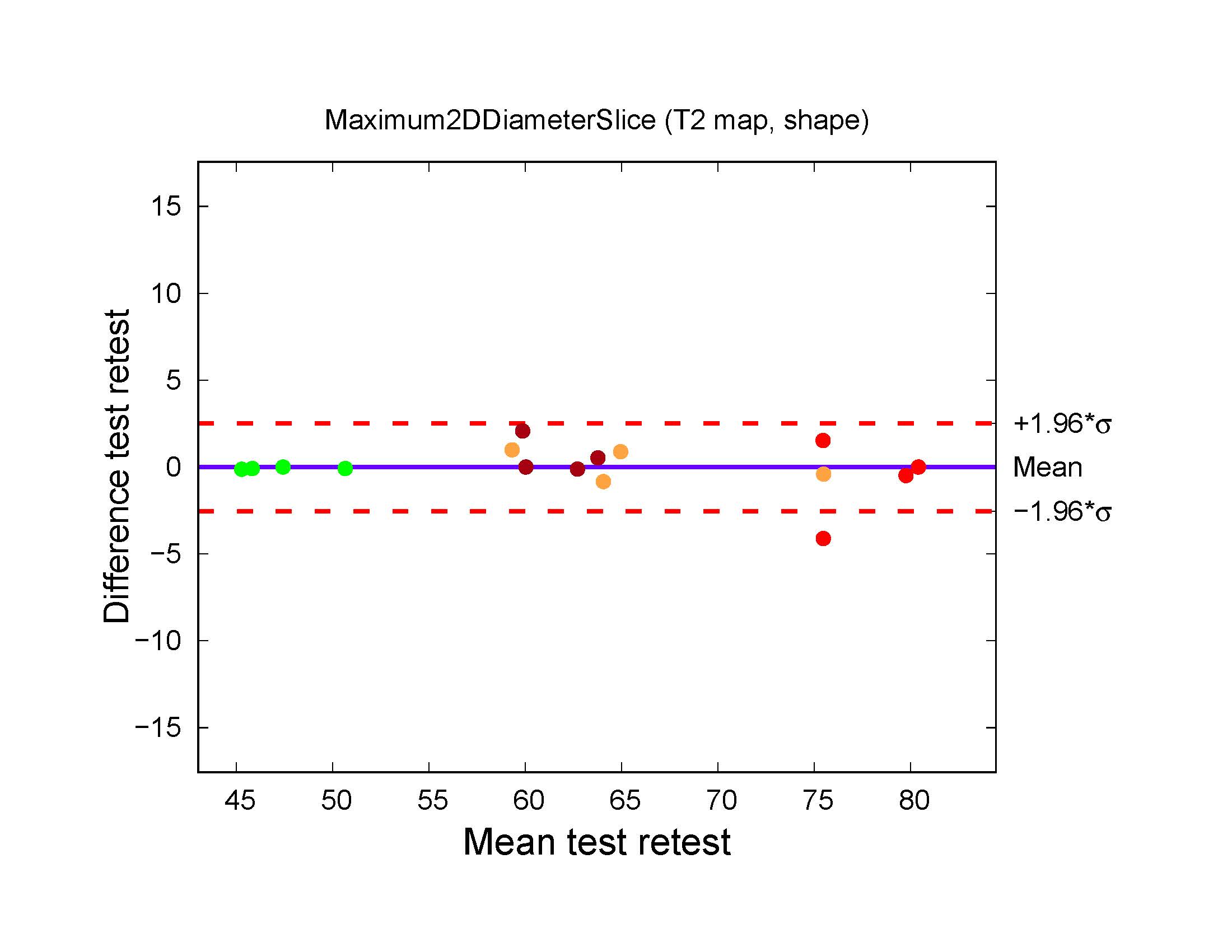


Rank 4:


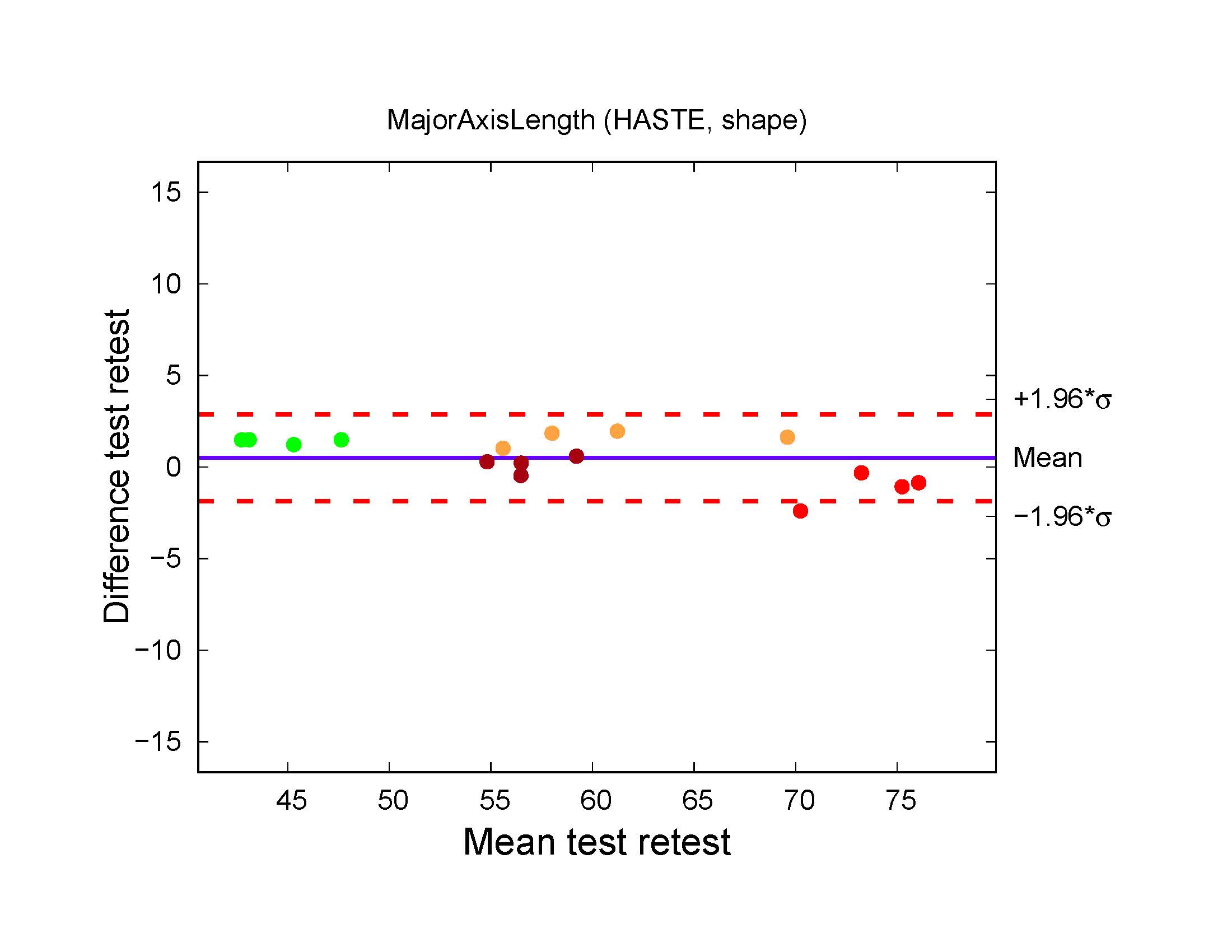


Rank 5:


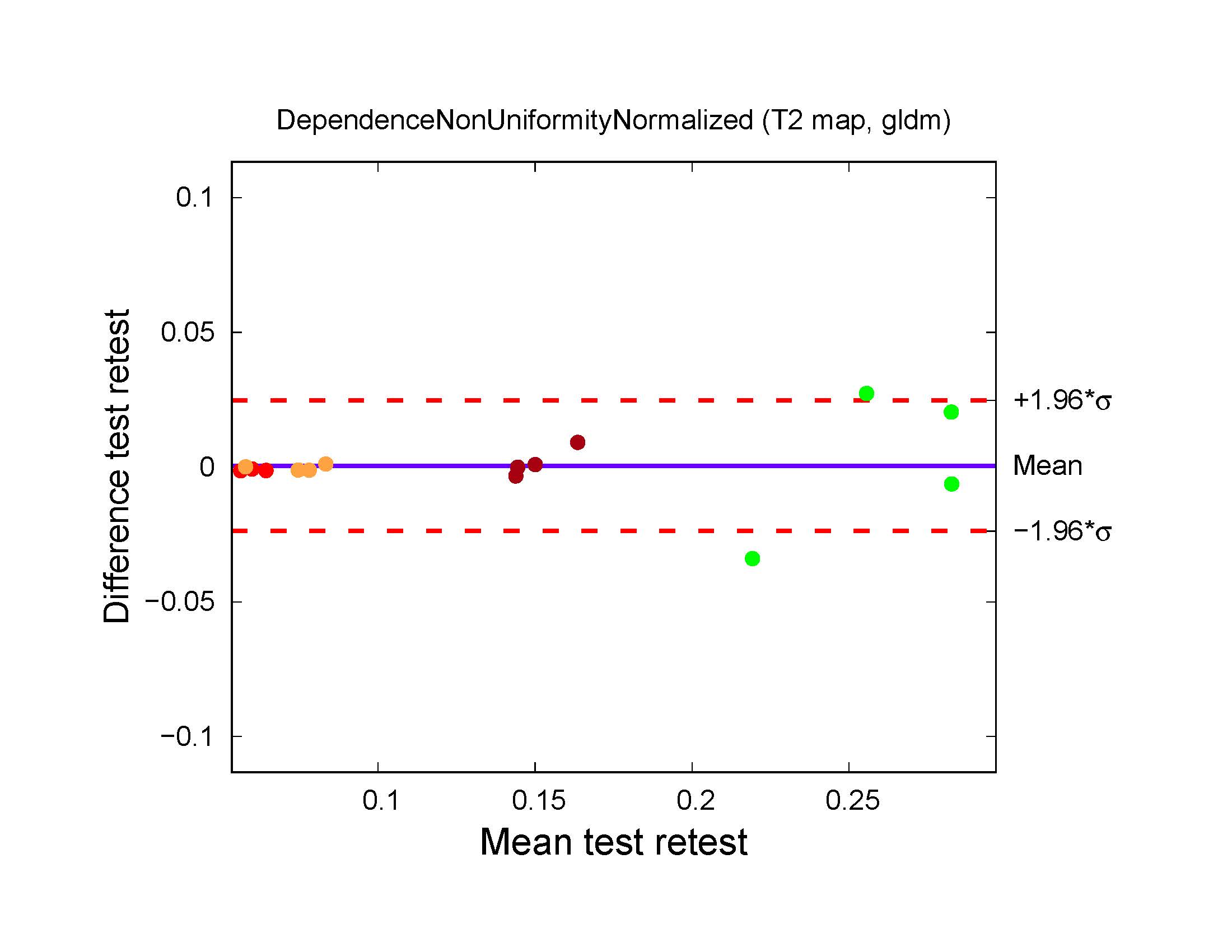


Rank 6:


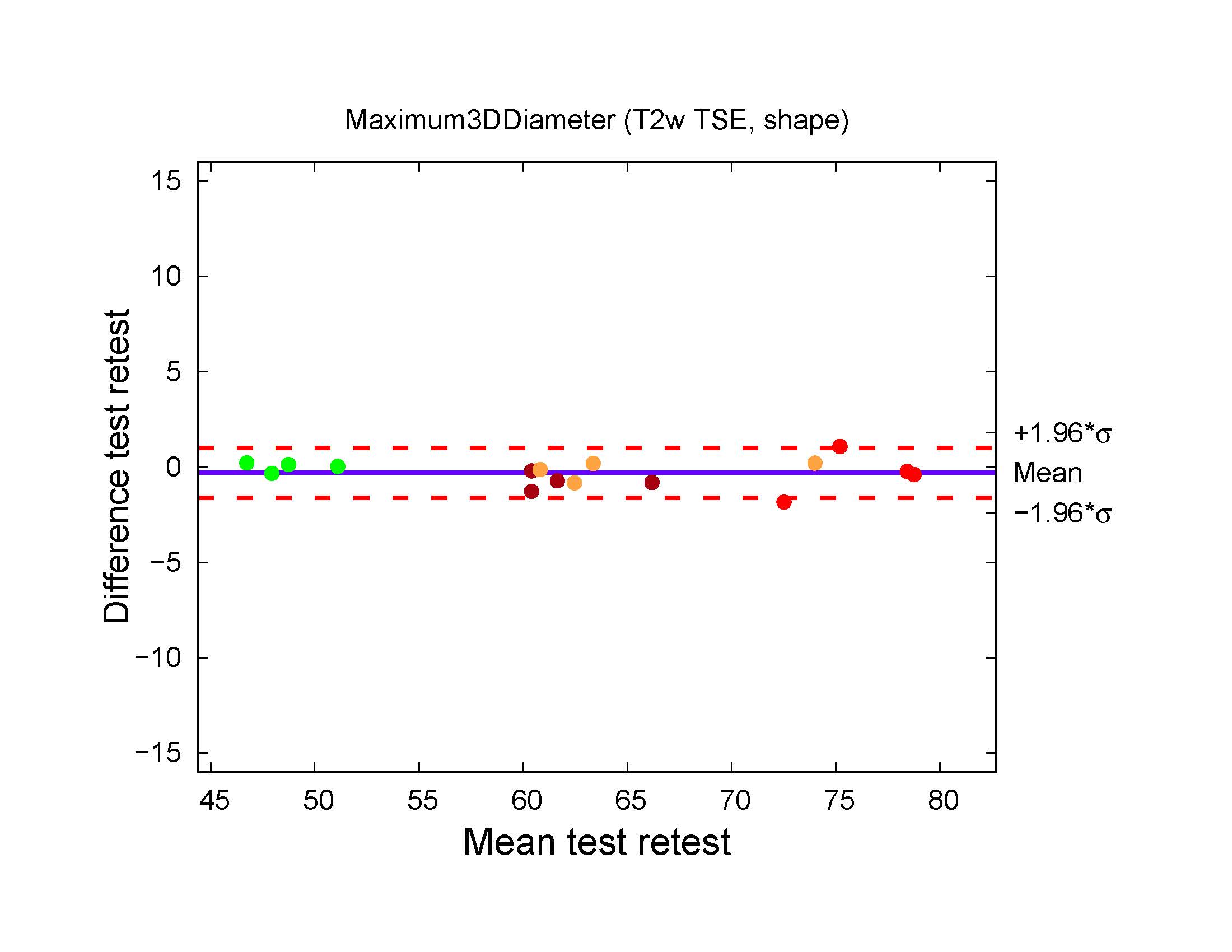

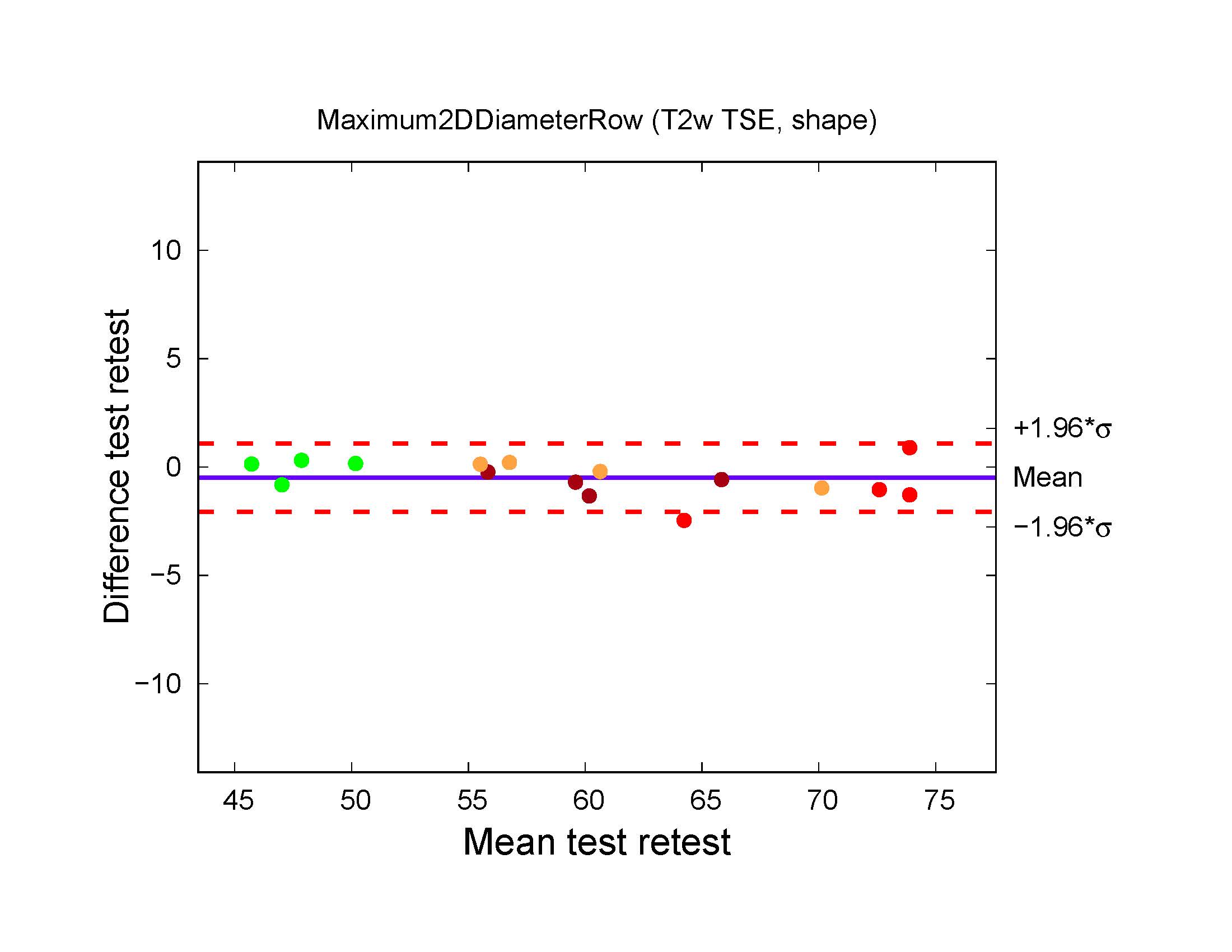

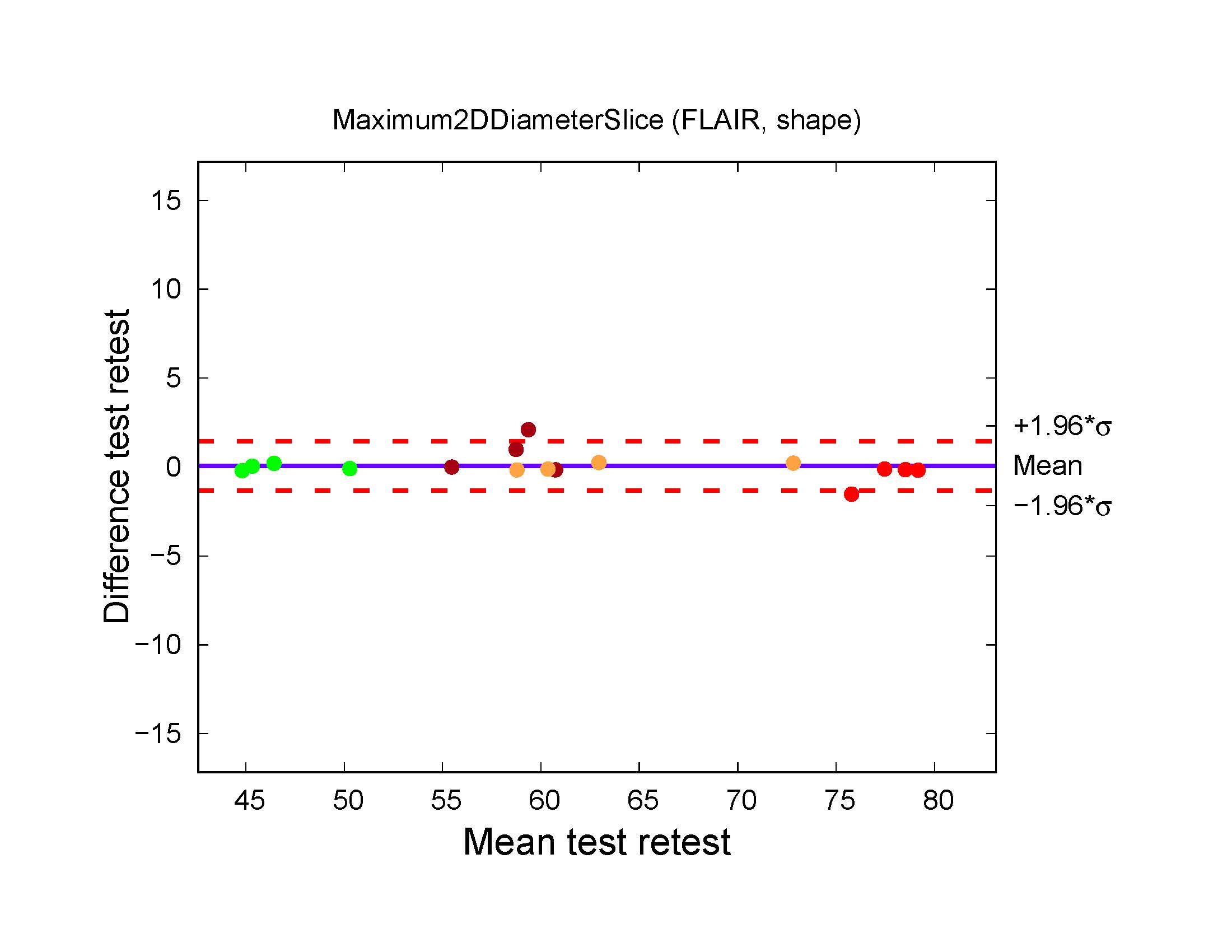

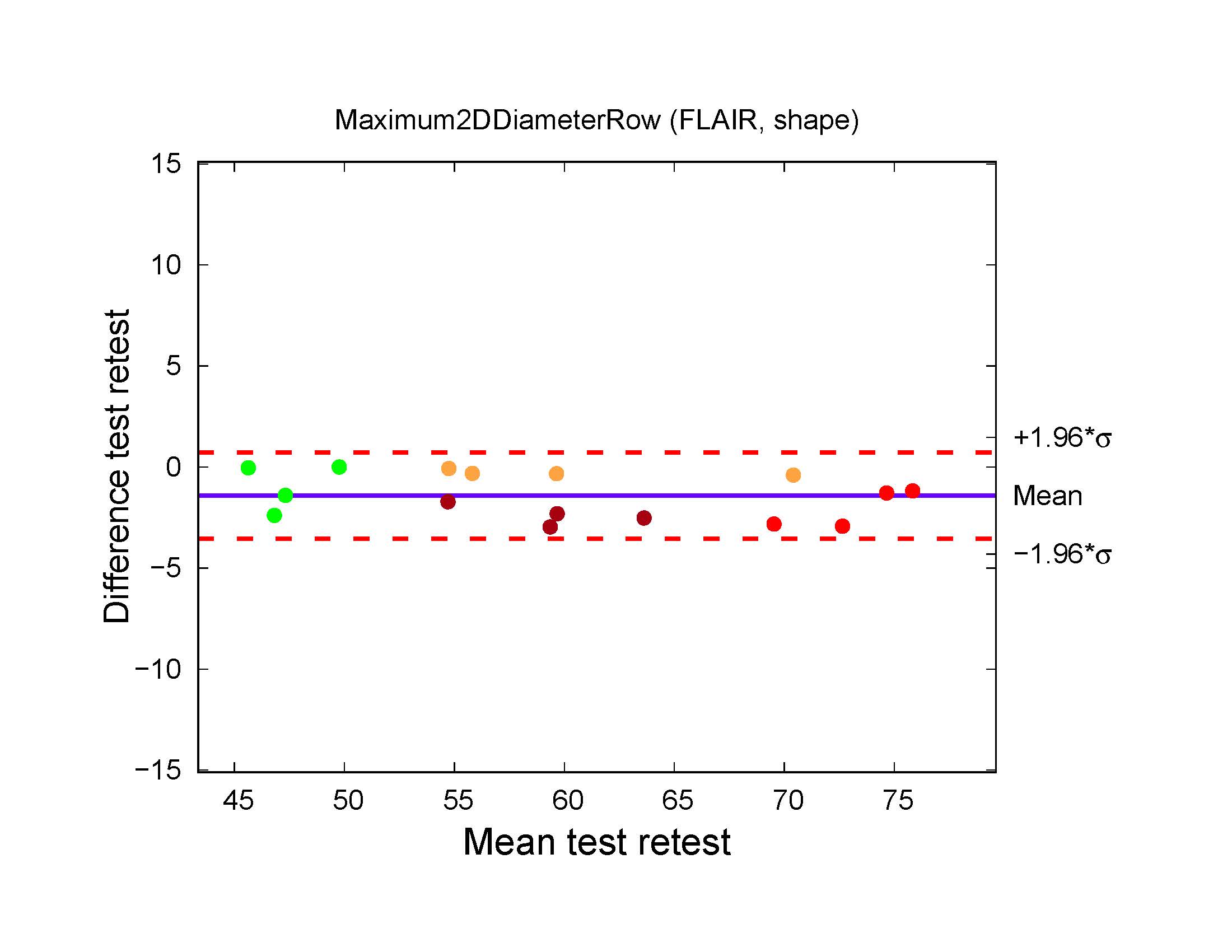

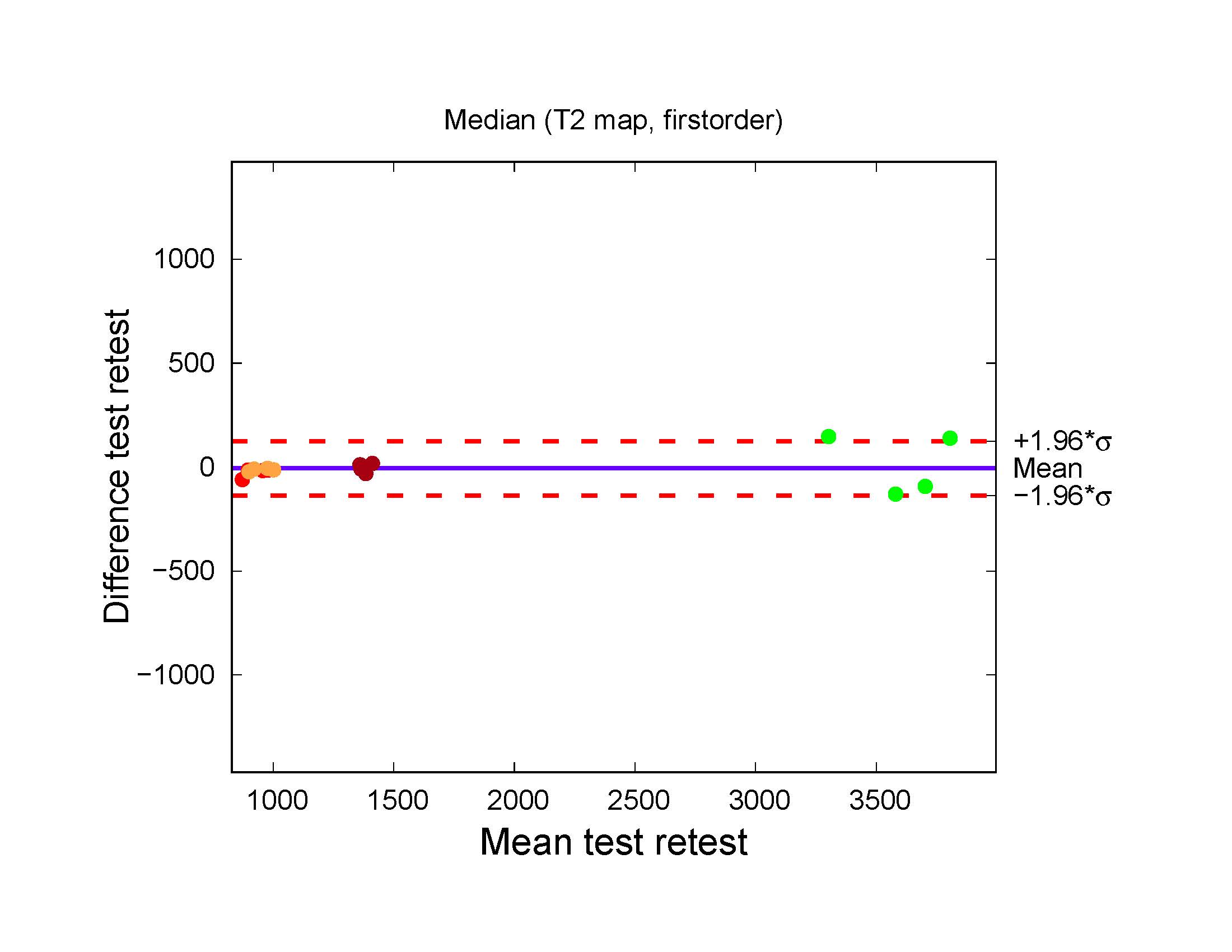


Rank 11:


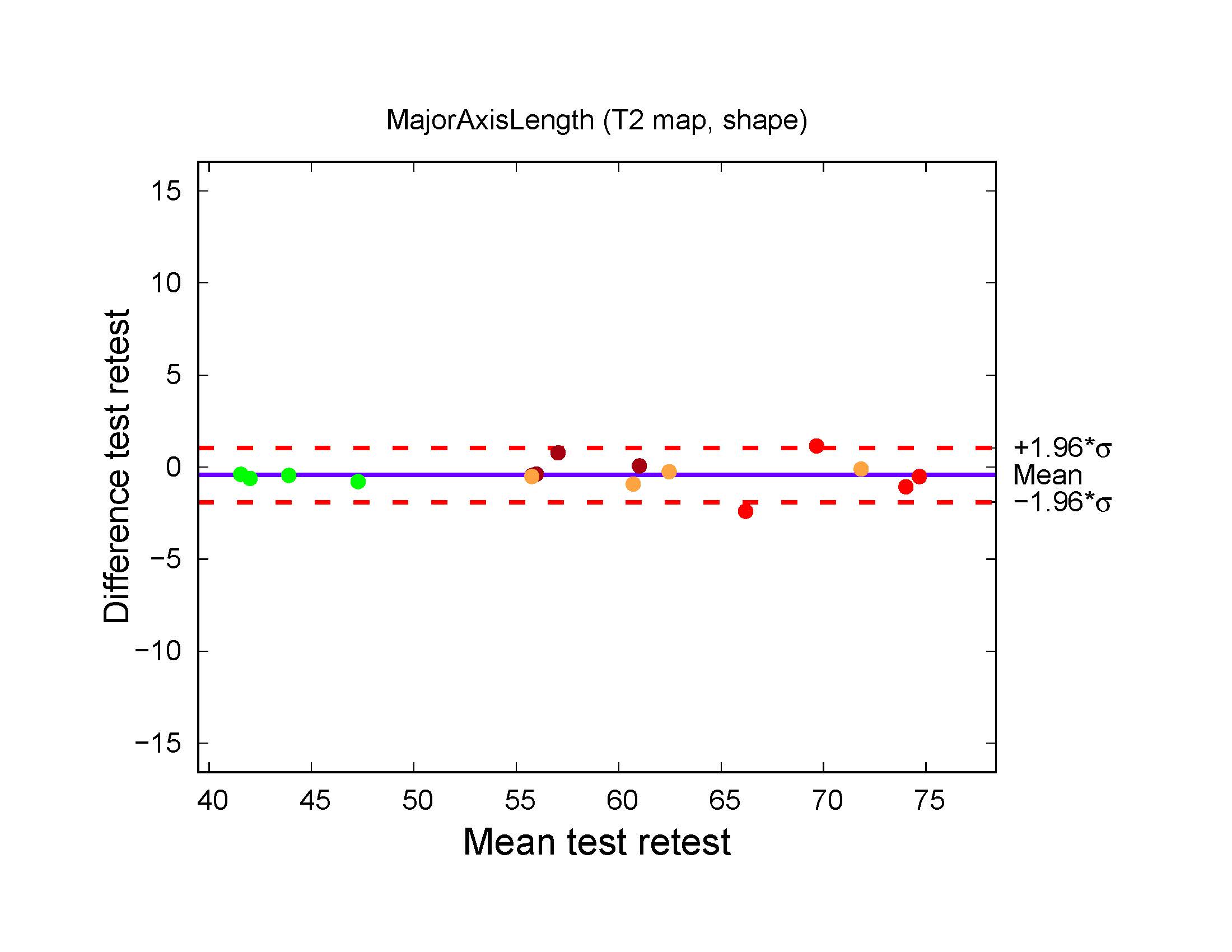

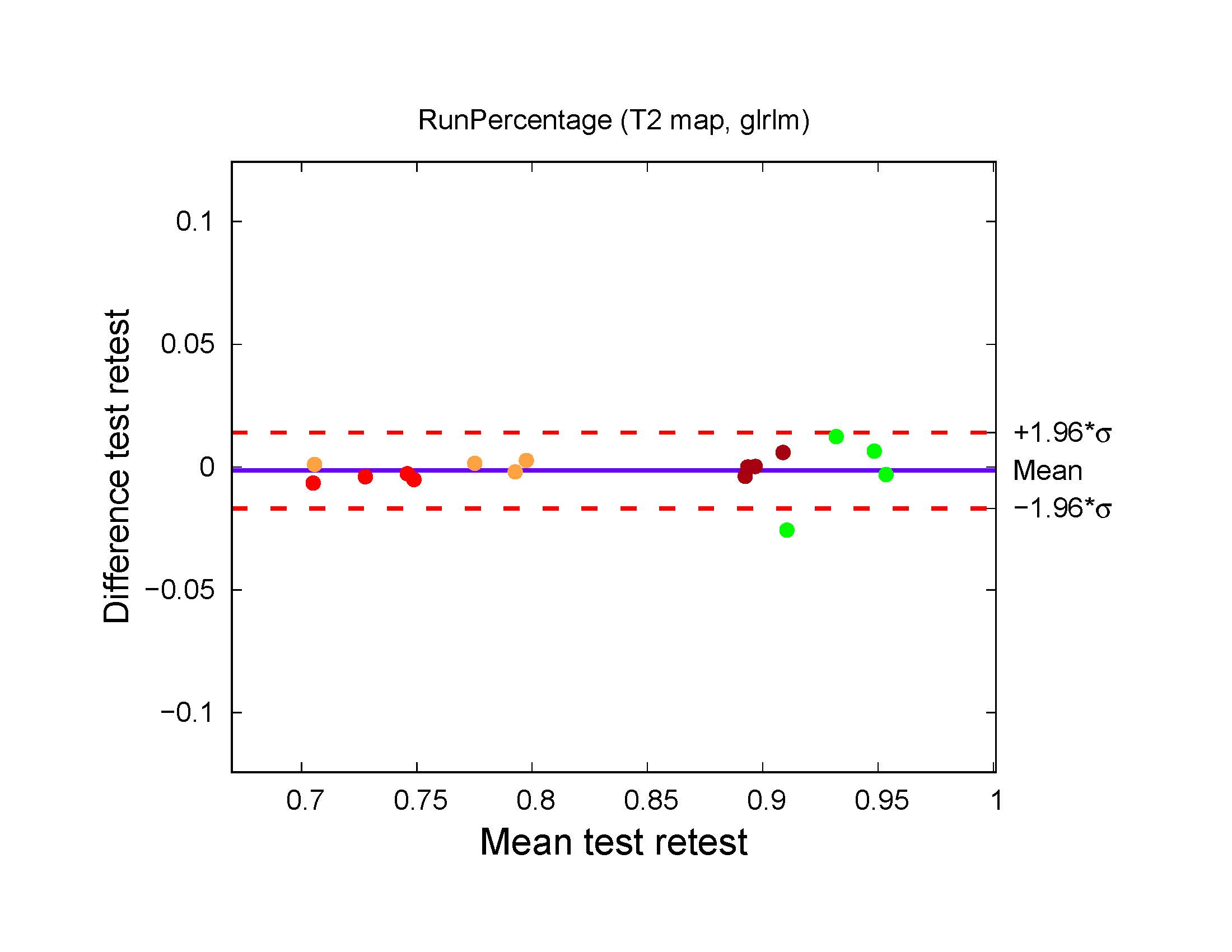

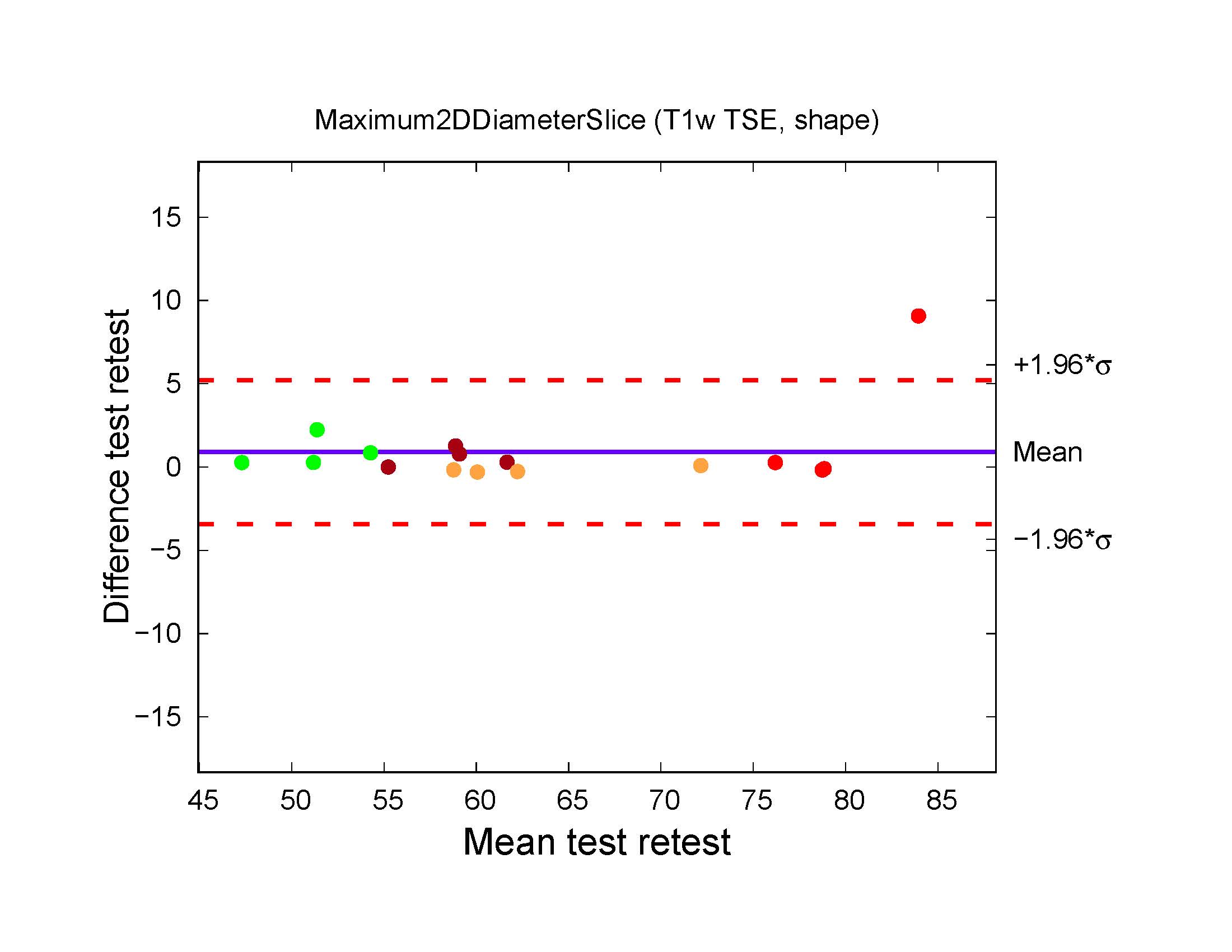

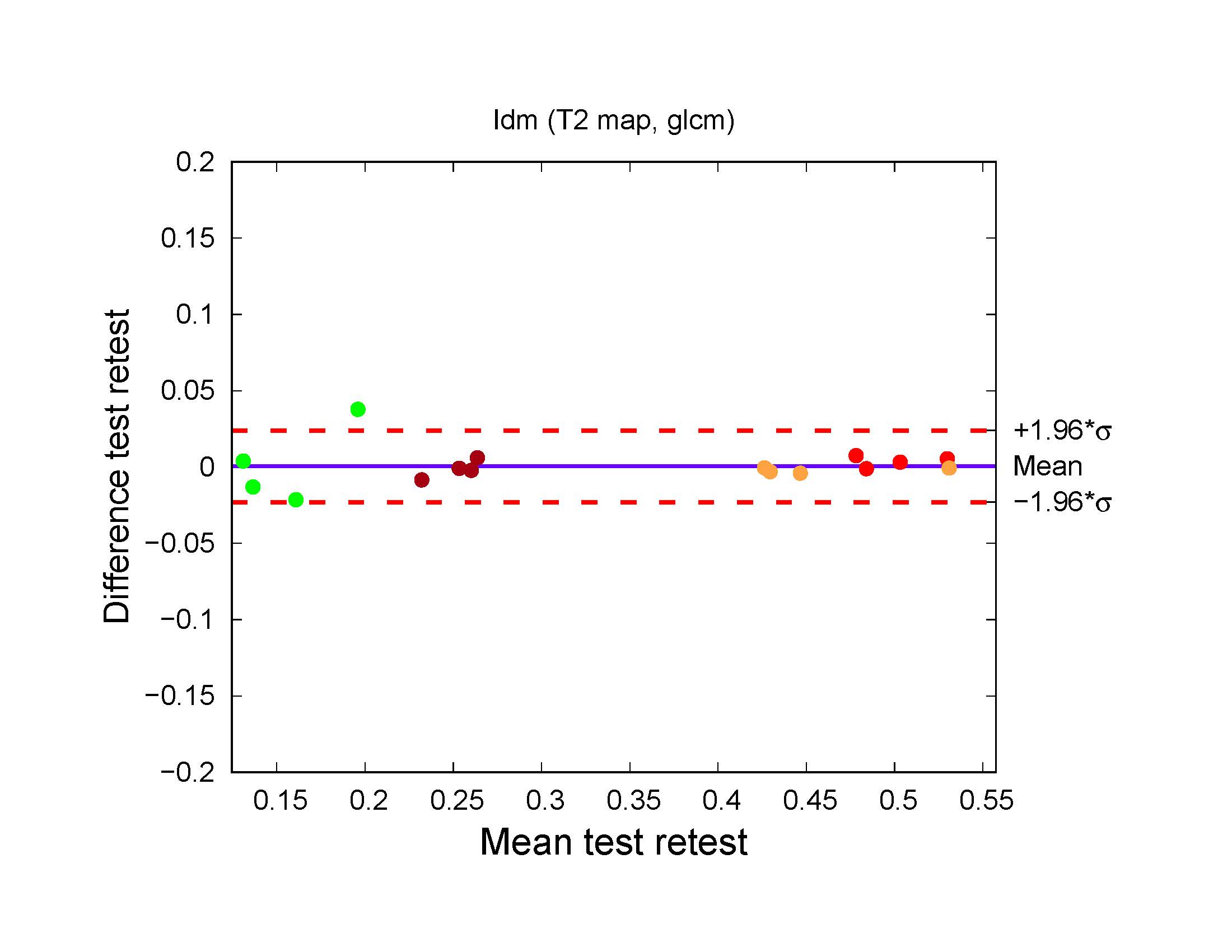

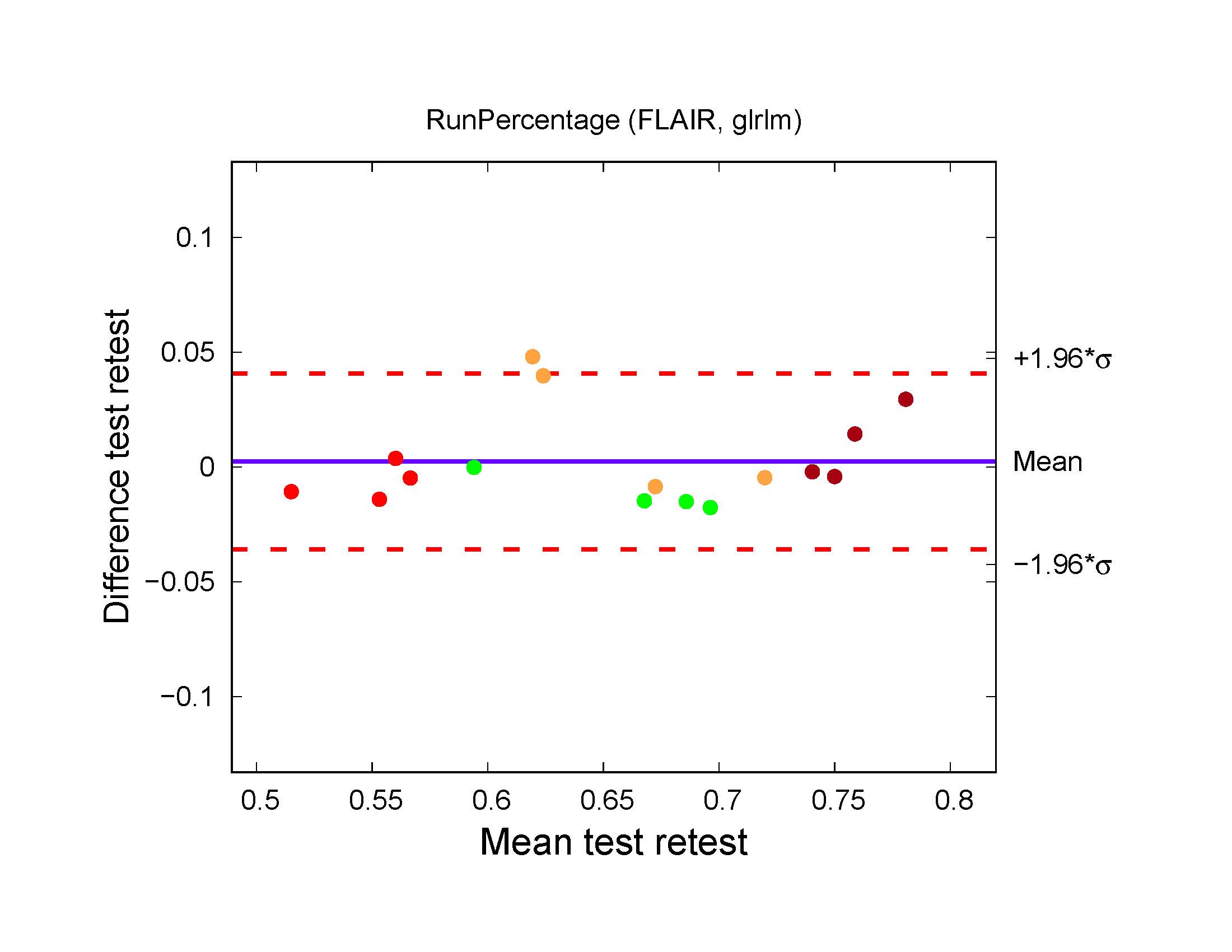

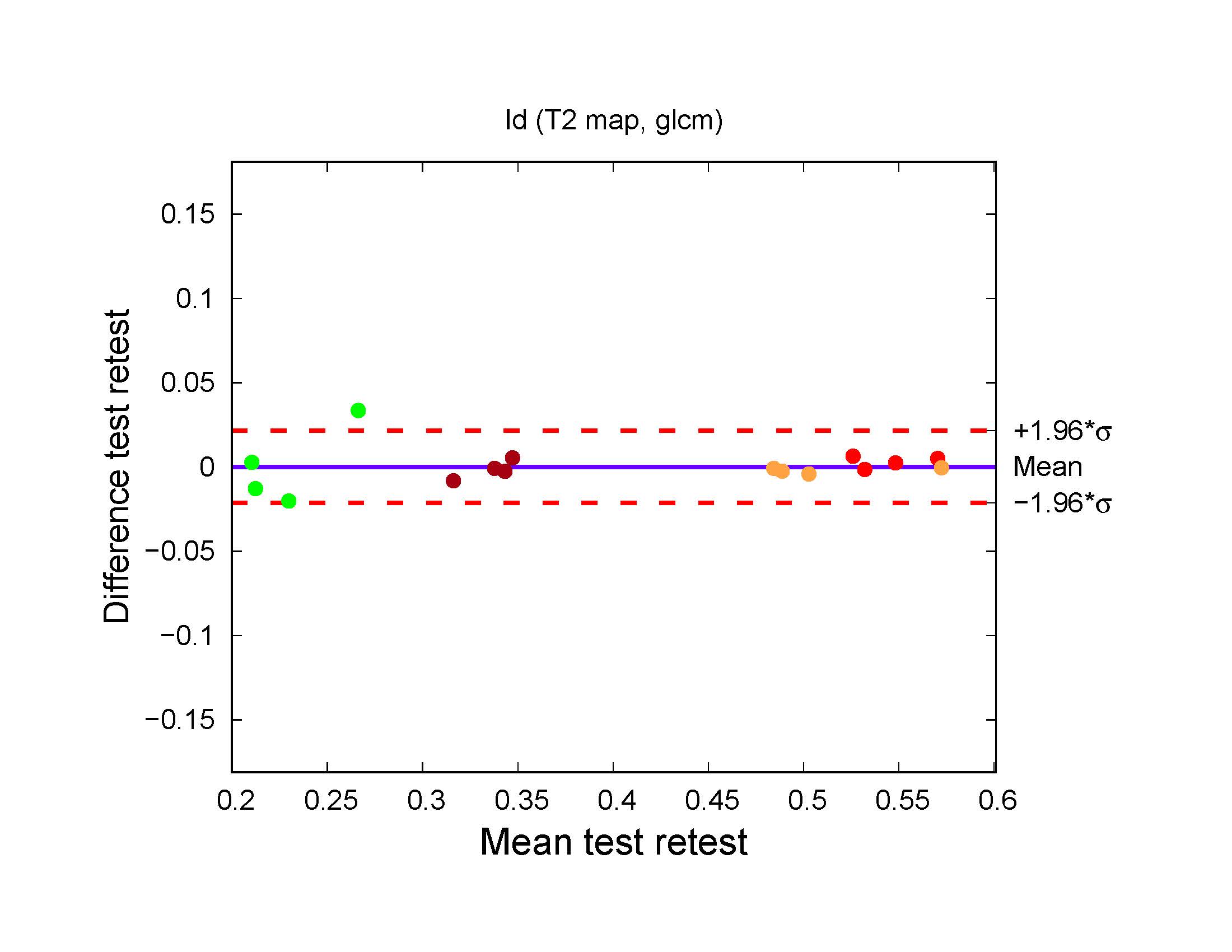

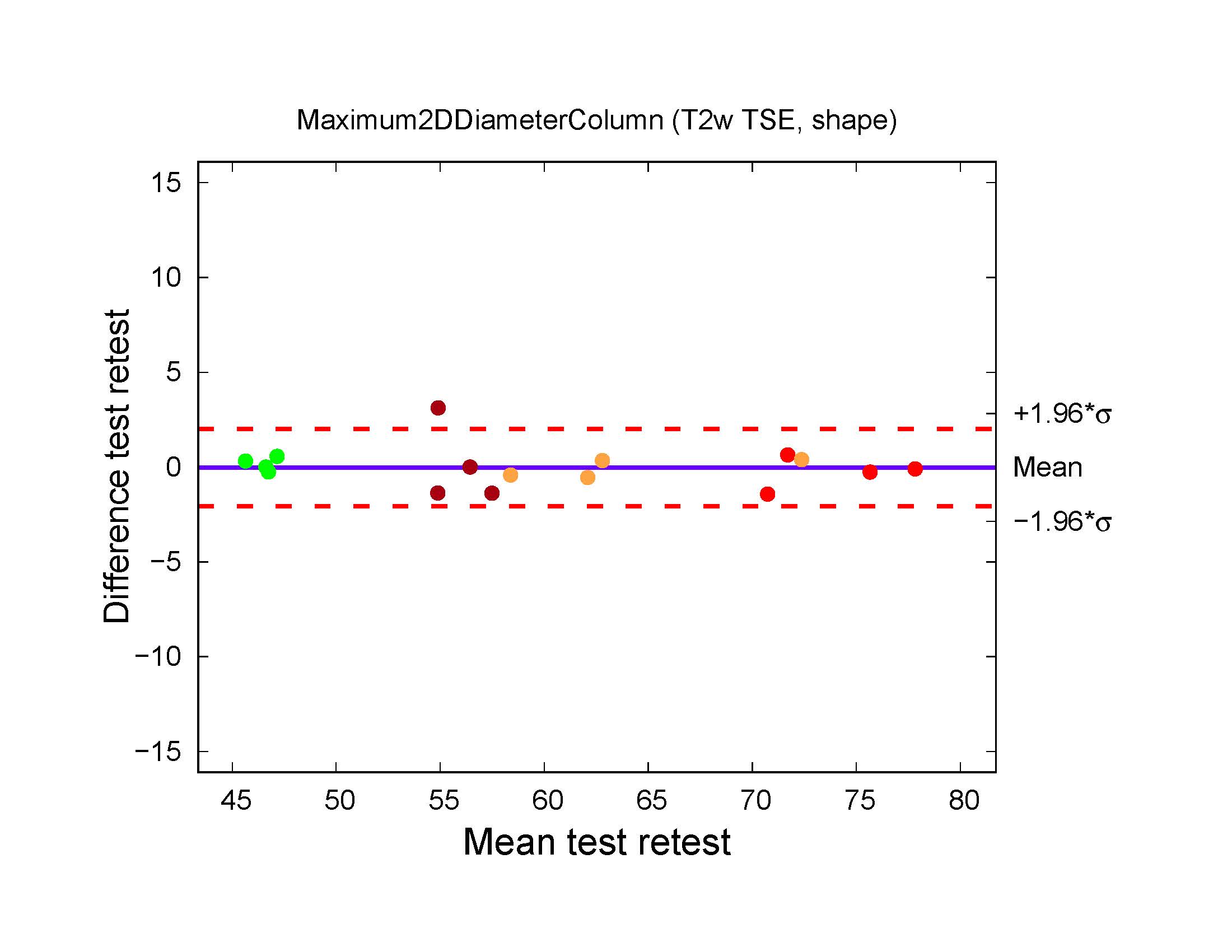

Supplement: Supplementary file 3 — Supplementary Figure 3. [file 41598_2021_93756_MOESM3_ESM.docx]
