# Supplementary material for: Impact of rescanning and repositioning on radiomic features employing a multi-object phantom in magnetic resonance imaging
Source: Sci Rep. 2021 Jul 9;11:14248. doi: 10.1038/s41598-021-93756-x (PMC8271025; doi:10.1038/s41598-021-93756-x)
Supplement: Supplementary file 6 — Supplementary Table Legends. [file 41598_2021_93756_MOESM6_ESM.docx]

**Supplementary Table Legends**

**Table A.1 .xls**

**Supplementary Table 1:** *Raw data on analysis of feature robustness and reproducibility*

Comprehensive tabular listing of all measurements of feature robustness and reproducibility for each feature, feature class and MRI sequence.

**Table D.1 .xls**

**Supplementary Table 2:**  *Features ranked by number of pairwise discriminative successes based on Gini score analysis*

Comprehensive tabular listing of all features of all feature classes and MRI sequences ranked according to the amount of successful pairwise discriminations of fruits. Columns depict discriminators for equal fruits, unequal fruits and all fruits in total.
